# Supplementary material for: A phosphorylation-regulated NPF transporter determines salt tolerance by mediating chloride uptake in soybean plants
Source: EMBO J. 2025 Jan 3;44(3):923–46. doi: 10.1038/s44318-024-00357-1 (PMC11790925; doi:10.1038/s44318-024-00357-1)
Supplement: Supplementary file 1 — Appendix [file 44318_2024_357_MOESM1_ESM.pdf]

Appendix for A phosphorylation-regulated NPF transporter determines salt tolerance by mediating chloride uptake in soybean plants

Yunzhen Wu, Jingya Yuan, Like Shen, Qinxue Li, Zhuomeng Li, Hongwei Cao, Lin Zhu, Dan Liu, Yalu Sun, Qianru Jia, Huatao Chen, Wubin Wang, Jörg Kudla, Wenhua Zhang, Junyi Gai, Qun Zhang

Table of contents

Appendix Figure S1………Pages 2
Appendix Figure S2………Pages 3
Appendix Figure S3………Pages 4
Appendix Figure S4………Pages 5–6
Appendix Figure S5………Pages 6
Appendix Figure S6………Pages 7
Appendix Figure S7………Pages 8
Appendix Figure S8………Pages 9–10
Appendix Table S1………Pages 11–13

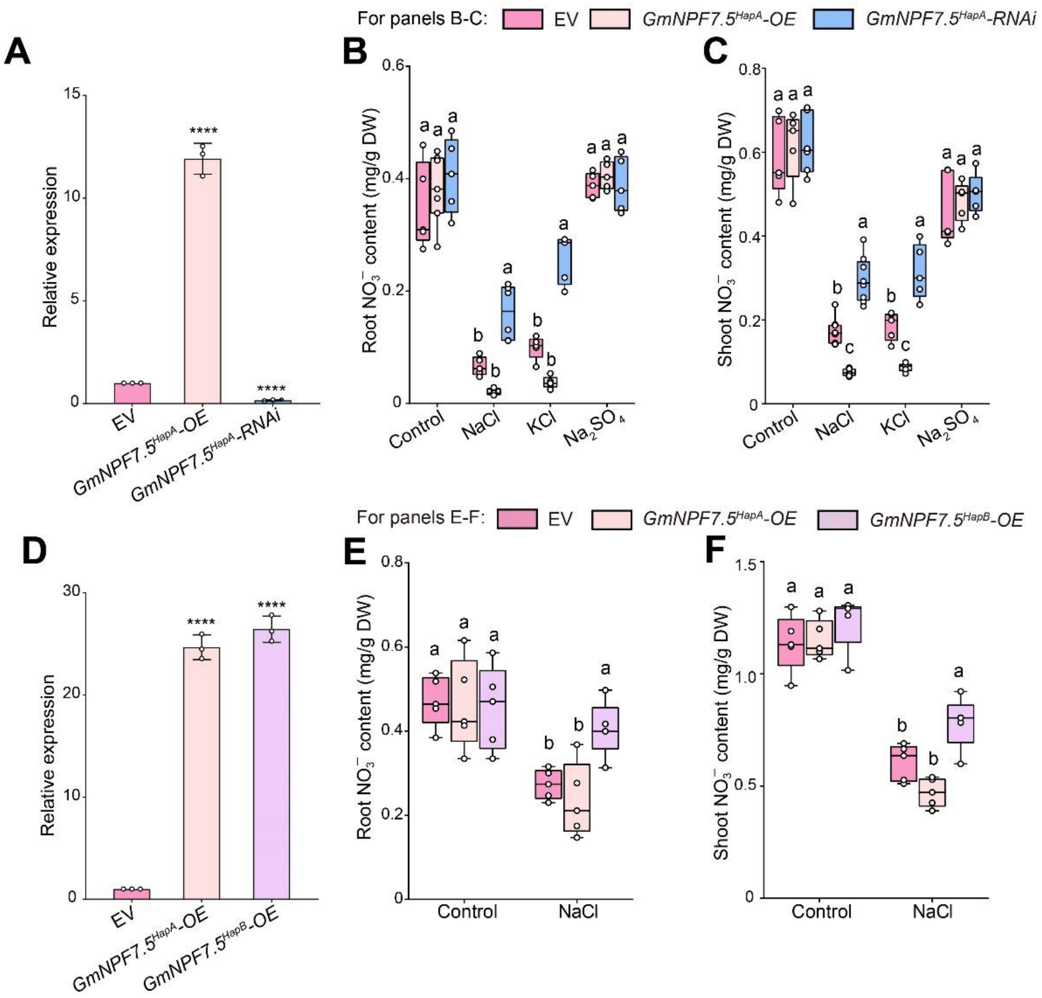

Appendix Figure S1. Comparison of NO_3^- content levels among soybean plants with transgenic hairy roots harboring the indicated constructs. (**A**) qRT-PCR analysis results for the expression in hairy roots of *GmNPF7.5* in *GmNPF7.5*^{HapA} overexpression and *GmNPF7.5*^{HapA} RNA interference transgenic soybean lines. *GmELF* was used as an internal control. Significance was determined using Student's two-sided *t*-test (****$P < 0.0001$; $n = 3$). (**B, C**) Root NO_3^- content (**B**) and shoot NO_3^- content (**C**) of soybean plants under the indicated conditions. (**D**) qRT-PCR analysis results for the expression in hairy roots of *GmNPF7.5* in *GmNPF7.5*^{HapA} overexpression and *GmNPF7.5*^{HapB} overexpression transgenic soybean lines. *GmELF* was used as an internal control. Significance was determined using a two-sided Student's *t*-test (****$P < 0.0001$; $n = 3$). (**E, F**) Root NO_3^- content (**E**) and shoot NO_3^- content (**F**) of transgenic soybean plants with hairy roots in *GmNPF7.5*^{HapA} overexpression or *GmNPF7.5*^{HapB} overexpression lines. Data in (**B, C**) and (**E, F**) are means ± SEM (n = 5–8 independent biological replicates). Significance was determined using one-way ANOVA, followed by Tukey's test. Different letters indicate significant differences ($P < 0.05$).

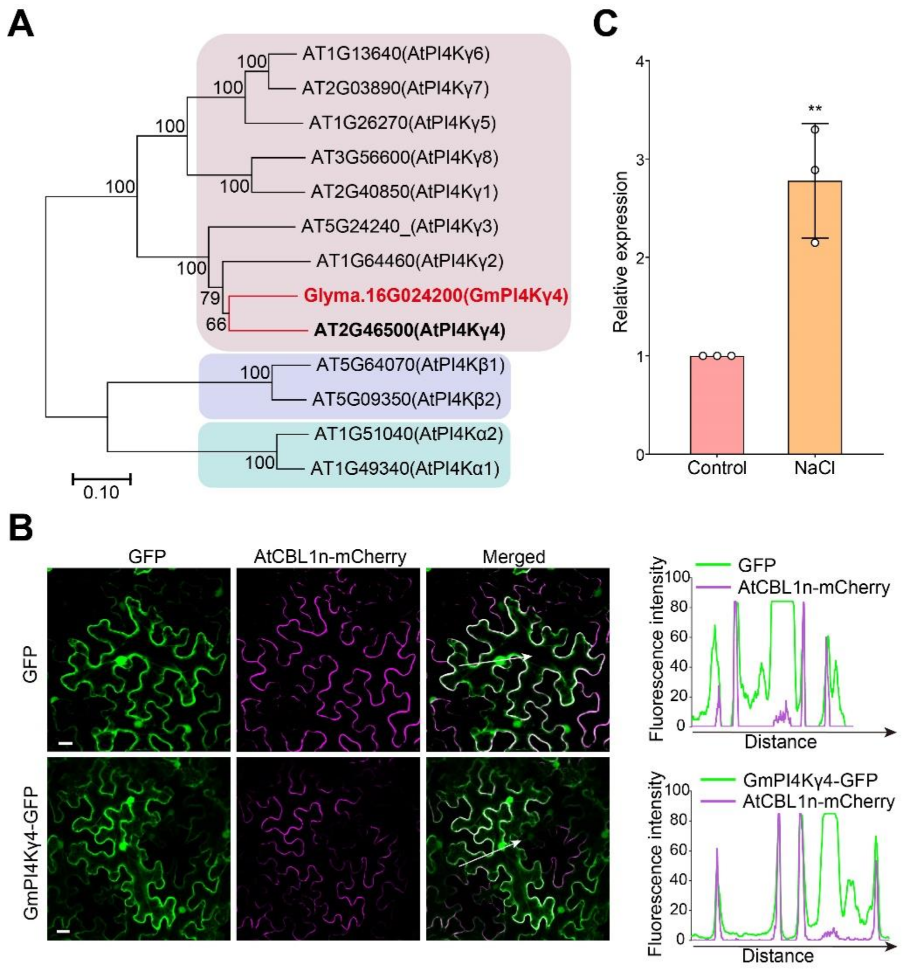

Appendix Figure S2. Expression pattern and subcellular localization of GmPI4Kγ4. (**A**) Phylogenetic analysis of *Arabidopsis* PI4K proteins and Glyma.16G02400 (GmPI4Kγ4). (**B**) Subcellular localization of GmPI4Kγ4–GFP in *N. benthamiana* leaves, shown by confocal images (left) and fluorescence intensity (right, arrows). Scale bars, 20 µm. (**C**) Transcript levels of *GmPI4Kγ4* in soybean plants under control and NaCl treatment. *GmELF* was used as an internal control. Significance was determined using a two-sided Student's *t*-test (***P* < 0.01; *n* = 3). The experiment was repeated three times, with similar results.

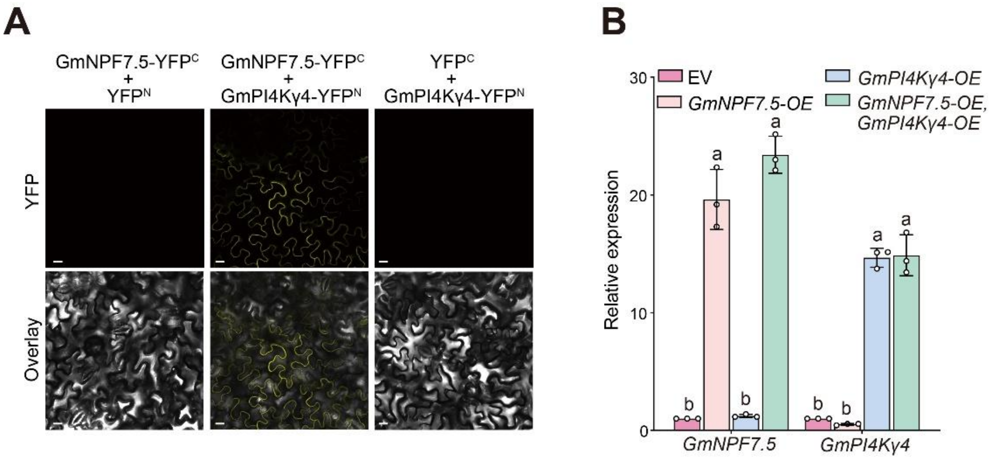

Appendix Figure S3. GmPI4Kγ4 interaction with GmNPF7.5 *in vivo* and *GmNPF7.5* and *GmPI4Kγ4* expression levels among transgenic soybean plants with hairy roots harboring indicated constructs. (**A**) Bimolecular fluorescence complementation (BiFC) assays of the interaction of GmNPF7.5 and GmPI4Kγ4 in *N. benthamiana* leaves. Scale bars, 20 μm. (**B**) qRT-PCR analysis of *GmNPF7.5* and *GmPI4Kγ4* expression levels in *GmNPF7.5* overexpression, *GmPI4Kγ4* overexpression, and *GmNPF7.5–GmPI4Kγ4* coexpression transgenic soybean plants with hairy roots. Plants harboring EV were used as the background control. Data are means ± SEM (n = 3). Significance was determined using one-way ANOVA, followed by Tukey's test. Different letters indicate significant differences ($P < 0.05$).

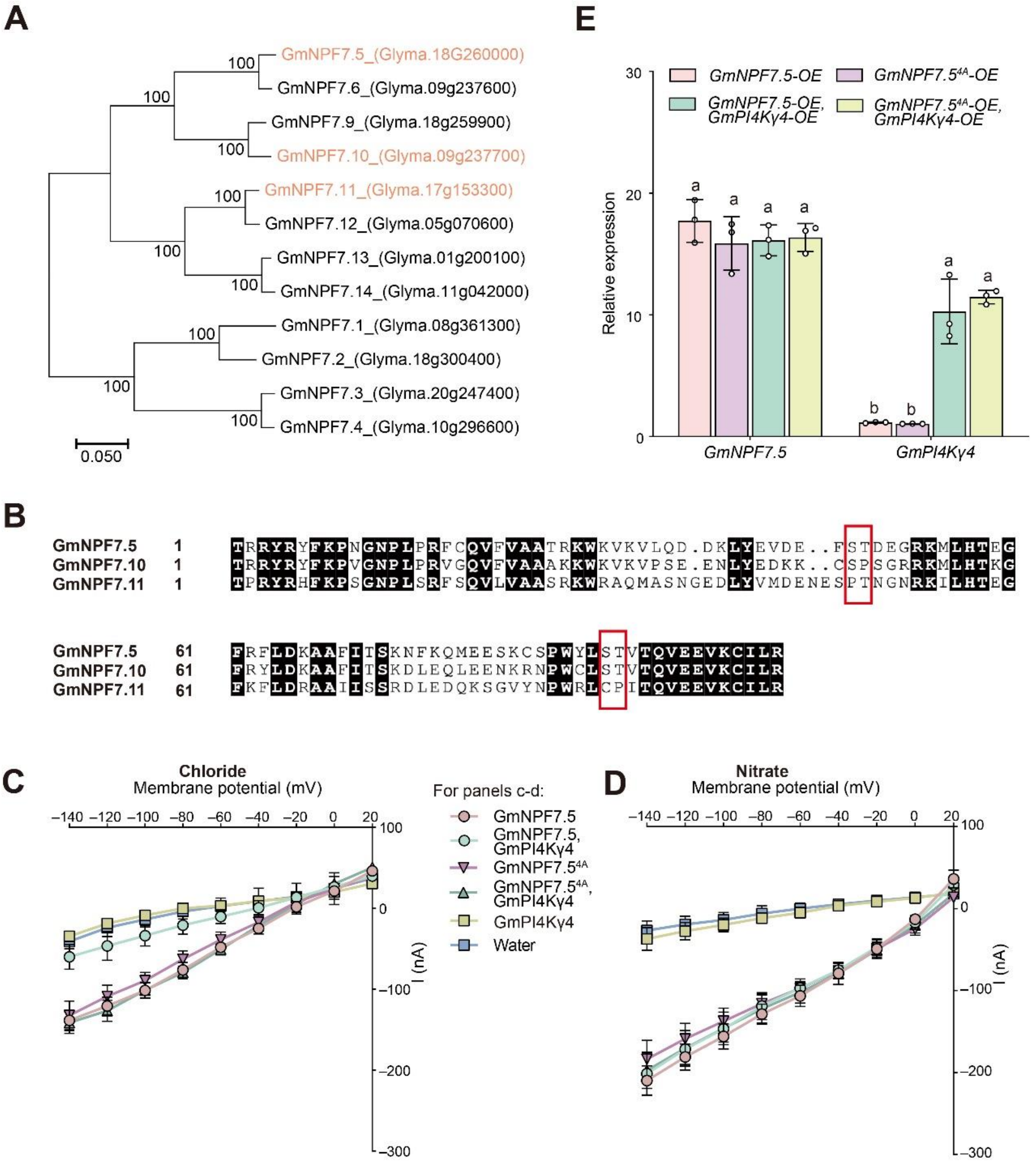

Appendix Figure S4. Identification of the phosphorylation sites of GmNPF7.5 by GmPI4Kγ4. (**A**) Phylogenetic clade of the NPF7 subfamily. The phylogenetic tree was constructed using MEGA7. (**B**) Amino acid sequence alignment among GmNPF7.5, GmNPF7.10, and GmNPF7.11 in the central linker domain. Non-conserved sites containing Ser/Thr in both GmNPF7.5 and GmNPF7.10 are indicated by red box. (**C, D**) I-V relationship for *Xenopus* oocytes expressing GmNPF7.5, GmNPF7.5^{4A}, GmPI4Kγ4, GmNPF7.5 + GmPI4Kγ4, or GmNPF7.5^{4A} + GmPI4Kγ4 in basal solution containing 10 mM Cl^- (**C**) or 10 mM NO_3^- (**D**) at pH 5.5 (n = 8–11). (**E**) *GmNPF7.5* and *GmPI4Kγ4* expression patterns in soybean plants harboring the indicated constructs. *GmELF* was used as an internal control. Data are means ± SEM (n = 3). Significance

was determined using one-way ANOVA, followed by Tukey’s test. Different letters indicate significant differences ($P < 0.05$).

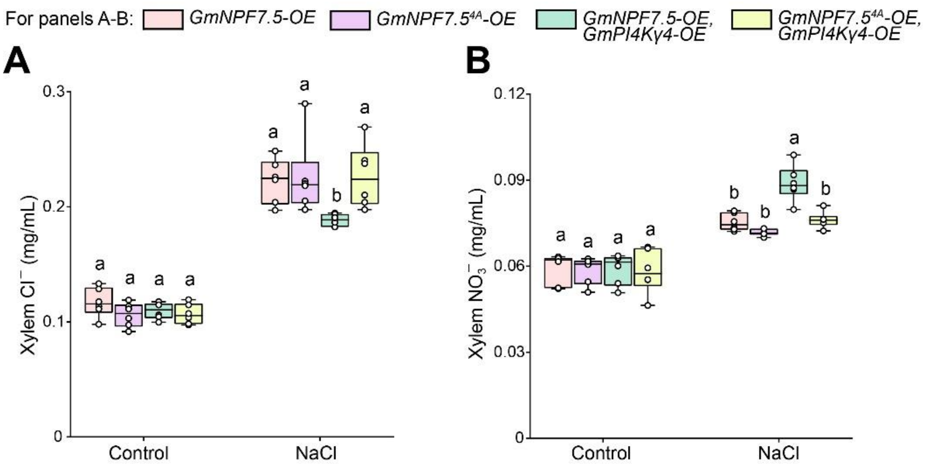

Appendix Figure S5. Comparison of Cl^- and NO_3^- concentrations in the xylem sap of transgenic soybean plants with hairy roots harboring the indicated constructs. (**A, B**) Concentrations of Cl^- (**A**) and concentrations of NO_3^- (**B**) in xylem sap of *GmNPF7.5* overexpression, *GmPI4Kγ4* overexpression, and *GmNPF7.5*–*GmPI4Kγ4* coexpression transgenic soybean plants with hairy roots. Data are means ± SEM (n = 4–6 independent biological replicates). Significance was determined using one-way ANOVA, followed by Tukey’s test. Different letters indicate significant differences ($P < 0.05$).

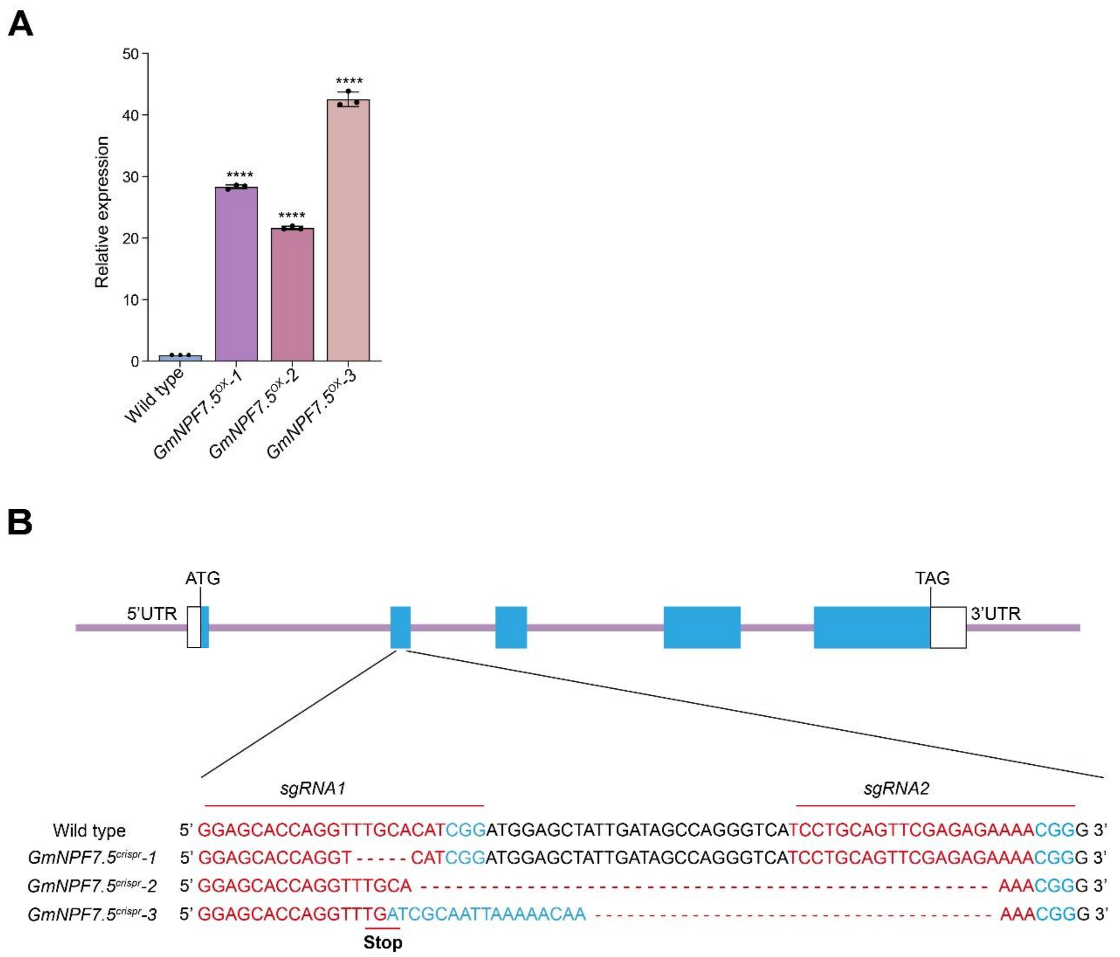

Appendix Figure S6. Identification of *GmNPF7.5* overexpression and *GmNPF7.5* crispr soybean lines. (**A**) *GmNPF7.5* transcript levels in WT and overexpression lines. *GmELF* was used as an internal control. Statistical significance was determined using a two-sided Student's *t*-test (****$P < 0.0001$; $n = 3$). (**B**) Schematic diagram of the gene structure of *GmNPF7.5* and the target sites of sgRNA for CRISPR lines. Blue and white boxes indicate exons and UTRs, respectively. Red line indicates target sites and mutations caused by CRISPR/Cas9 editing in three independent mutant lines. The protospacer adjacent motif (PAM) sequence is highlighted in blue.

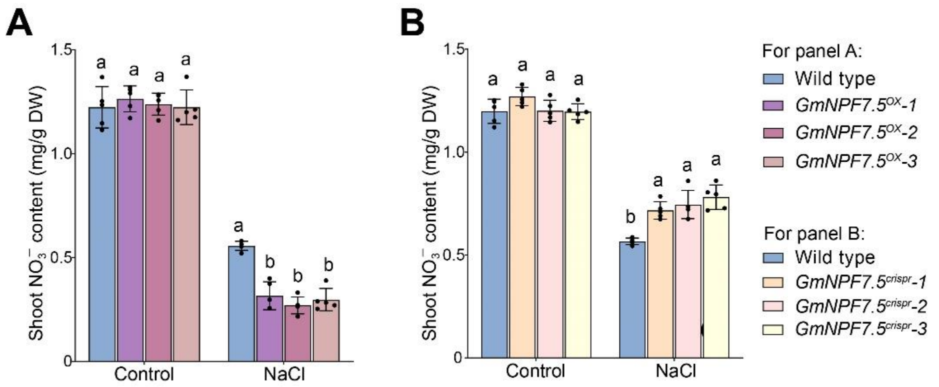

Appendix Figure S7. NO_3^- content levels of GmNPF7.5 stable transgenic soybean plants with indicated treatments. NO_3^- content levels of *GmNPF7.5^{OX}* and *GmNPF7.5^{crispr}* plants under control and NaCl treatment. Data are means ± SEM (n = 5 independent biological replicates). Significance was determined using one-way ANOVA, followed by Tukey's test. Different letters indicate significant differences ($P < 0.05$).

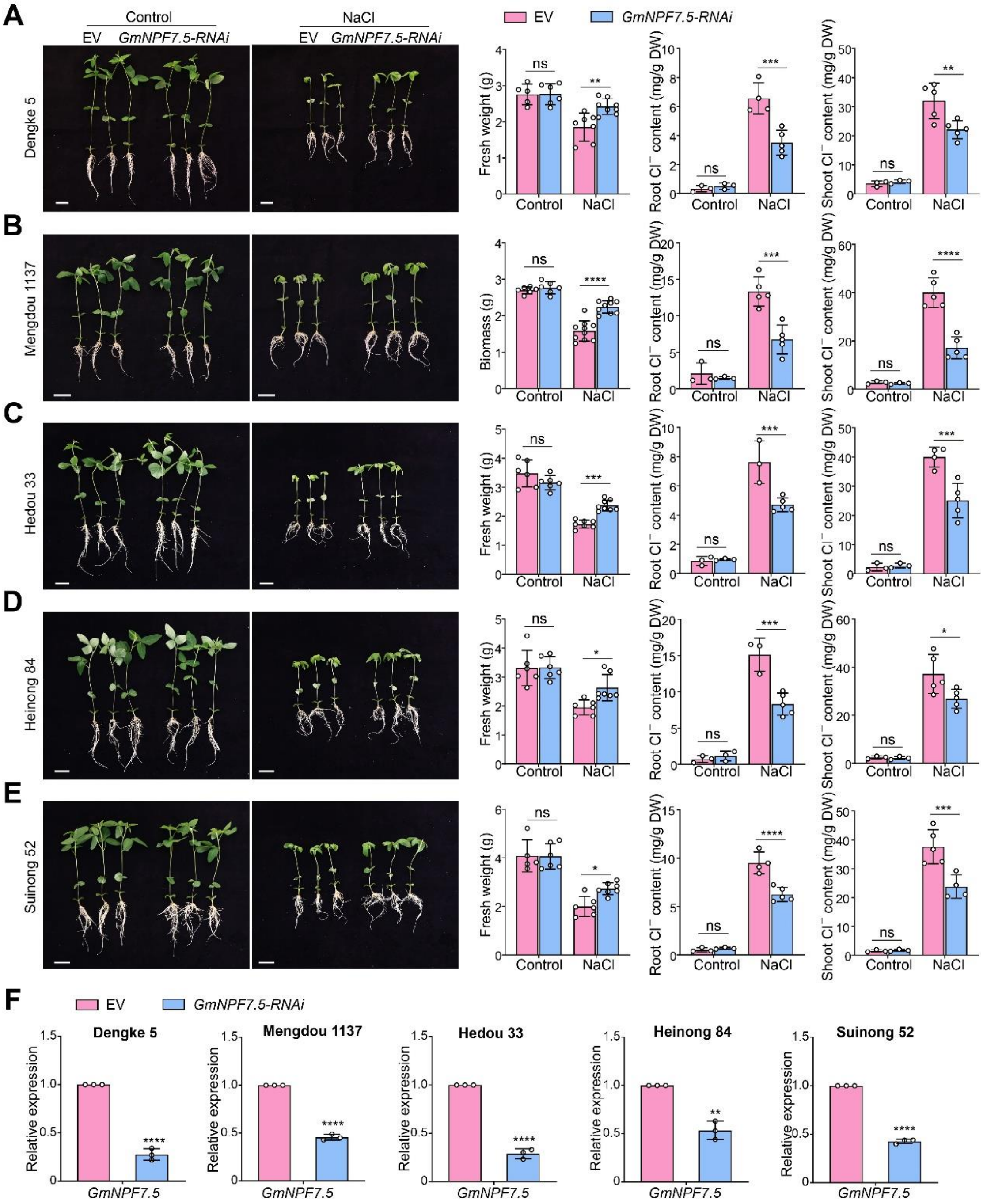

Appendix Figure S8. Functional analysis of GmNPF7.5 in five soybean cultivars (‘Dengke 5,’ ‘Mengdou 1137,’ ‘Hedou 33,’ ‘Heinong 84,’ and ‘Suinong 52’) under salt stress. (A–E) Phenotype, biomass, and root and shoot Cl^- contents were examined in hairy root transgenic soybean plants derived from five cultivars harboring *GmNPF7.5*-RNAi or EV. Scale bars, 5 cm. Significance was determined using a two-sided Student’s *t*-test (*$P < 0.05$, **$P < 0.01$, ***$P < 0.001$, ****$P < 0.0001$; n = 3–10). ns, nonsignificant. **(F)** qRT-PCR analysis of *GmNPF7.5* expression in *GmNPF7.5*–RNAi hairy root transgenic soybean plants derived from different cultivars. *GmELF* was used as an internal control. Significance was determined using a two-sided

Student's *t*-test (***P* < 0.01, *****P* < 0.0001; n = 3).

Appendix Table S1. Nucleotide sequences of primers used for construction and quantitative qRT-PCR. Cutting sites of restriction enzymes are underlined.

Primer name	Sequence (5′ to 3′)
GmNPF7.5-F	ATGGGTTGTTTGTATTTTCT
GmNPF7.5-R	CTACACTACTTCAGGGTCTT
GmNPF7.5-promoter-F	CATGTGGGGATTTTTGTTGG
GmNPF7.5-promoter-R	TACTGGGACTAATCTTGCAA
GmNPF7.5-GFP-F	TAGTGGATCCATGGGTTGTTTGTATTTTCT
GmNPF7.5-GFP-R	CGGGGGATCCCTACACTACTTCAGGGTCTT
GmNPF7.5-pBA002-F	GGACTCTAGAATGGGTTGTTTGTATTTTCT
GmNPF7.5-pBA002-R	ATCCTCTAGACTACACTACTTCAGGGTCTT
GmNPF7.5-RNAi-F	ATGGGTTGTTTGTATTTTCT
GmNPF7.5-RNAi-R	CTAAAGGAAGGCTCCAAGAA
GmNPF7.5-pGEMHE-F	CCGGGGATCCATGGGTTGTTTGTATTTTCT
GmNPF7.5-pGEMHE-R	TTGCTCTAGACTACACTACTTCAGGGTCTT
ZmNPF6.6-pGEMHE-F	CCGGGGATCCATGGCCTCCGTCCTGCCGGA
ZmNPF6.6-pGEMHE-R	TTGCTCTAGATCAGTGGCCGACGGCAATAG
AtNPF6.3-pGEMHE-F	CCGGGGATCCATGTCTCTTCCTGAAACTAA
AtNPF6.3-pGEMHE-R	TTGCTCTAGATCAATGACCCATTGGAATAC
GmPI4Kγ4-GFP-F	TAGTGGATCCATGTCGTCTGCTGGTGTCAC
GmPI4Kγ4-GFP-R	CGGGGGATCCCTAATCAAGGTGCTGGTCCA
GmNPF7.5CL-BD-F	GGCCGAATTCACACGAAGGTATAGATACTT
GmNPF7.5CL-BD-R	CCGGGAATTCCTATCTTAGAATGCATTTCA
GmPI4Kγ4-AD-F	CAGTGAATTCATGTCGTCTGCTGGTGTCAC
GmPI4Kγ4-AD-R	GGTGGAATTCCTAATCAAGGTGCTGGTCCA
GmNPF7.5CL-GST-F	CCCGGAATTCACACGAAGGTATAGATACTT
GmNPF7.5CL-GST-R	CCGGGAATTCCTATCTTAGAATGCATTTCA
GmPI4Kγ4-His-F	ATCCGAATTC ATGTCGTCTGCTGGTGTCAC
GmPI4Kγ4-His-R	GCTCGAATTCATCAAGGTGCTGGTCCATGA
GmNPF7.5CL-GFP-F	TAGTGGATCCACACGAAGGTATAGATACTT
GmNPF7.5CL-GFP-R	CGGGGGATCCCTATCTTAGAATGCATTTCA
GmPI4Kγ4-Flag-F	TAGTGGATCCATGTCGTCTGCTGGTGTCAC
GmPI4Kγ4-Flag-R	CGGGGGATCCATCAAGGTGCTGGTCCATGA

GmNPF7.5-pGmUbi-F	ACAGTCTAGAATGGGTTGTTTGTATTTTCT
GmNPF7.5-pGmUbi-R	CCATGGTACCCACTACTTCAGGGTCTTCTT
GmPI4Kγ4- pUB-GFP-F	ACAGTCTAGAATGTCGTCTGCTGGTGTCAC
GmPI4Kγ4- pUB-GFP-R	CCATGGTACCATCAAGGTGCTGGTCCATGA
GmNPF7.5-cYFP-F	ACAGGGTACCATGGGTTGTTTGTATTTTCT
GmNPF7.5-cYFP-R	CTGGGGATCCCACTACTTCAGGGTCTTCTT
GmPI4Kγ4-nYFP-F	ACAGGGTACCATGTCGTCTGCTGGTGTCAC
GmPI4Kγ4-nYFP-R	CTGGGGATCCATCAAGGTGCTGGTCCATGA
GmPI4Kγ4-pGEMHE-F	CCGGGGATCCATGTCGTCTGCTGGTGTCAC
GmPI4Kγ4-pGEMHE-R	TTGCTCTAGACTAATCAAGGTGCTGGTCCA
GmPI4Kγ4-GST-F	CCCGGAATTCATGTCGTCTGCTGGTGTCAC
GmPI4Kγ4-GST-R	CCGGGAATTCCTAATCAAGGTGCTGGTCCA
GmNPF7.10CL-GST-F	CCCGGAATTCATGGGTTGTTTGTATTTTAT
GmNPF7.10CL-GST-R	CCGGGAATTCCTATCTTAGTATGCACTTCA
GmNPF7.11CL-GST-F	CCGGGAATTCATGGCCTGCTTAGAAGTCAG
GmNPF7.11CL-GST-R	CCGGGAATTCCTATCTTAGTATGCACTTCA
Crispr-GmNPF7.5-F	ACTCTATGTTGTAACTTAGCAAG
Crispr-GmNPF7.5-R	TGAGAGTTTCACTCTCTAGCTA
**Primers for qRT-PCR**	
RT-GmELF-F	GTTGAAAAGCCAGGGGACA
RT-GmELF-R	TCTTACCCCTTGAGCGTGG
RT-GmNPF7.5-F	GCCACAGATGAGATGCCAGG
RT-GmNPF7.5-R	TACAAGATCAGCTGCGGTGAG
RT-Glyma.03G135800-F	ACATGGAAGGCATGGACCAAA
RT-Glyma.03G135800-R	TTGCTCACTCTTGCACTTCTCT
RT-Glyma.02G108000-F	TCCTAAGACTGGGAAACCTGCT
RT-Glyma.02G108000-R	GGCCATGAGCCATCCTTGA
RT-Glyma.03G135800-F	ATATGCTCGTGGACGATCCG
RT-Glyma.03G135800-R	GATCACGAGCAAACTCCGCT
RT-Glyma.09G014900-F	GGTCTGCAGTAAGTGGCTGT
RT-Glyma.09G014900-R	ATTCTCACAGCAGGACTCGC
RT-Glyma.13G053900-F	CACATCTGCATTGGTGTGGC

RT-Glyma.13G053900-R	GGTTTTGGCCTAGACTCCCC
RT-Glyma.13G323800-F	ATGTTGGGTCCACGCTATGG
RT-Glyma.13G323800-R	CCAATCAAGAACCGAACCGC
RT-Glyma.09G050200-F	TGCCAAGAGTTTGGGGTGTT
RT-Glyma.09G050200-R	ATGAAGCCCAAAAAGAAGGTAGT
RT-Glyma.15G172300-F	TGGAGAAAGAGGAGGATCATGGA
RT-Glyma.15G172300-R	CTTGCGTTCTTGGATGTGCTT
RT-Glyma.16G059900-F	TGGTTCAGAGCTCAAAGGGT
RT-Glyma.16G059900-R	GTGAATGGAACCGTCCCTGG
RT-Glyma.18G231500-F	TTCTCCCCAATGCTGAAGCC
RT-Glyma.18G231500-R	TGTGGAGGAATTGAGCGAGT
RT-Glyma.18G260700-F	TTCTCCCTTGTTTGGTCTGGT
RT-Glyma.18G260700-R	TGGTGACTAAACTCCCCTGG
RT-GmPI4Kγ4-F	CCATCCTGGAGACTTGACTGC
RT-GmPI4Kγ4-R	CCCAGCATGTCTATCTGCGT
