# Supplementary figures and images for: A phosphorylation-regulated NPF transporter determines salt tolerance by mediating chloride uptake in soybean plants
Source: EMBO J. 2025 Jan 3;44(3):923–46. doi: 10.1038/s44318-024-00357-1 (PMC11790925; doi:10.1038/s44318-024-00357-1)

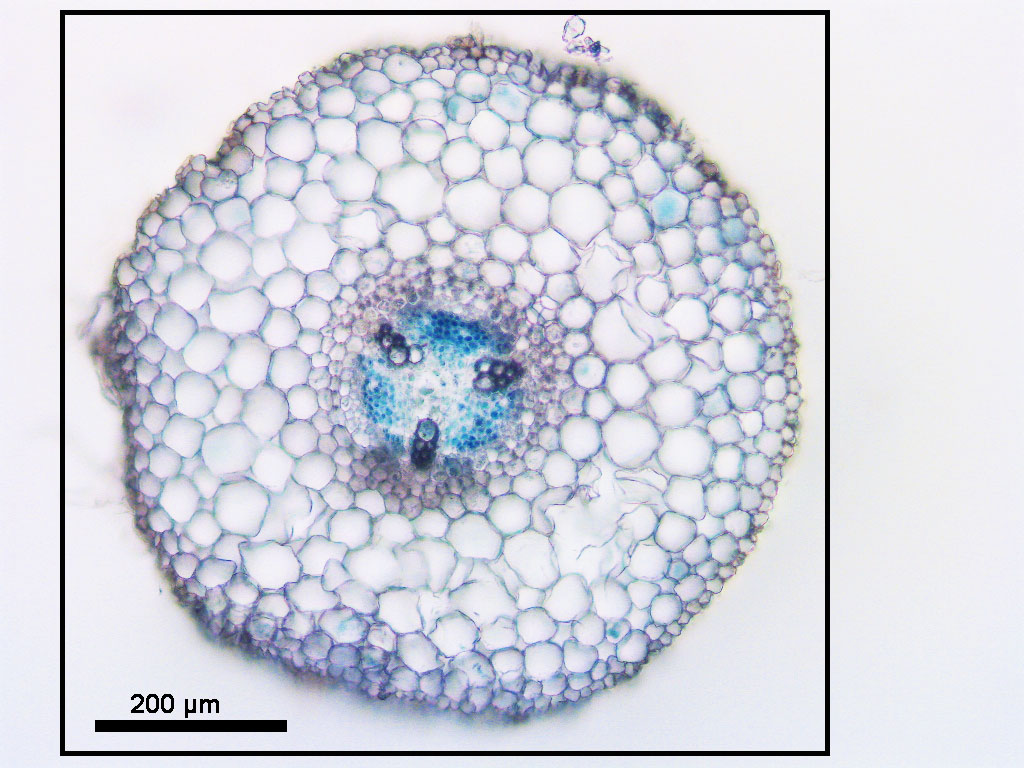

Supplement: Supplementary file 3 — Source data Fig. 1 [file 44318_2024_357_MOESM3_ESM.zip › Figure 1/Fig.1E/Control section.jpg]

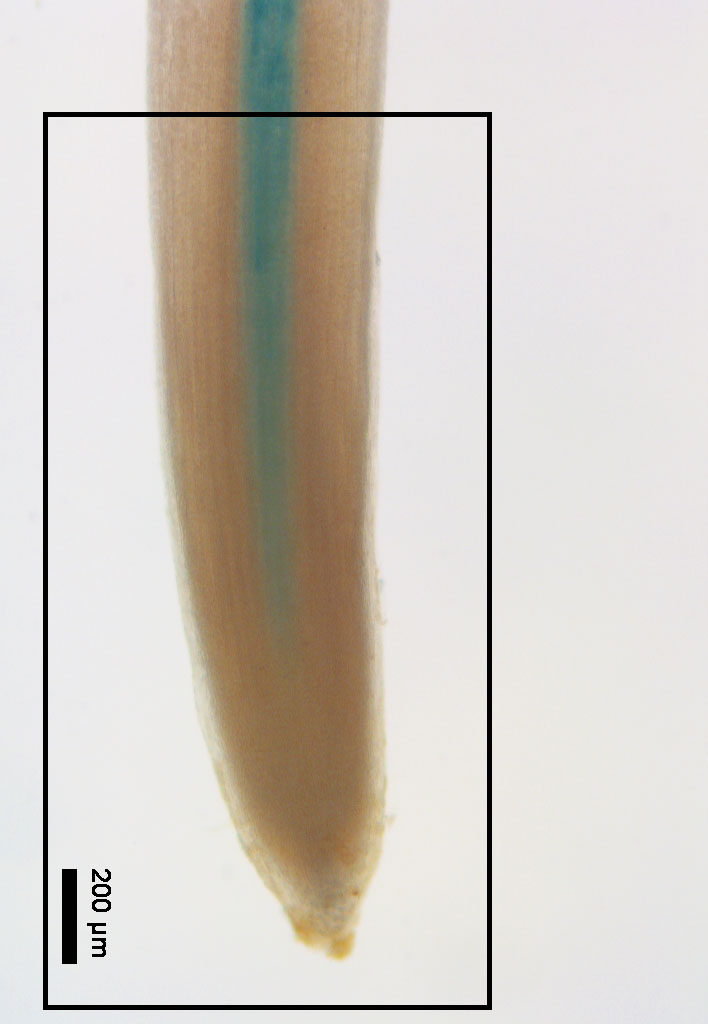

Supplement: Supplementary file 3 — Source data Fig. 1 [file 44318_2024_357_MOESM3_ESM.zip › Figure 1/Fig.1E/Control-1.jpg]

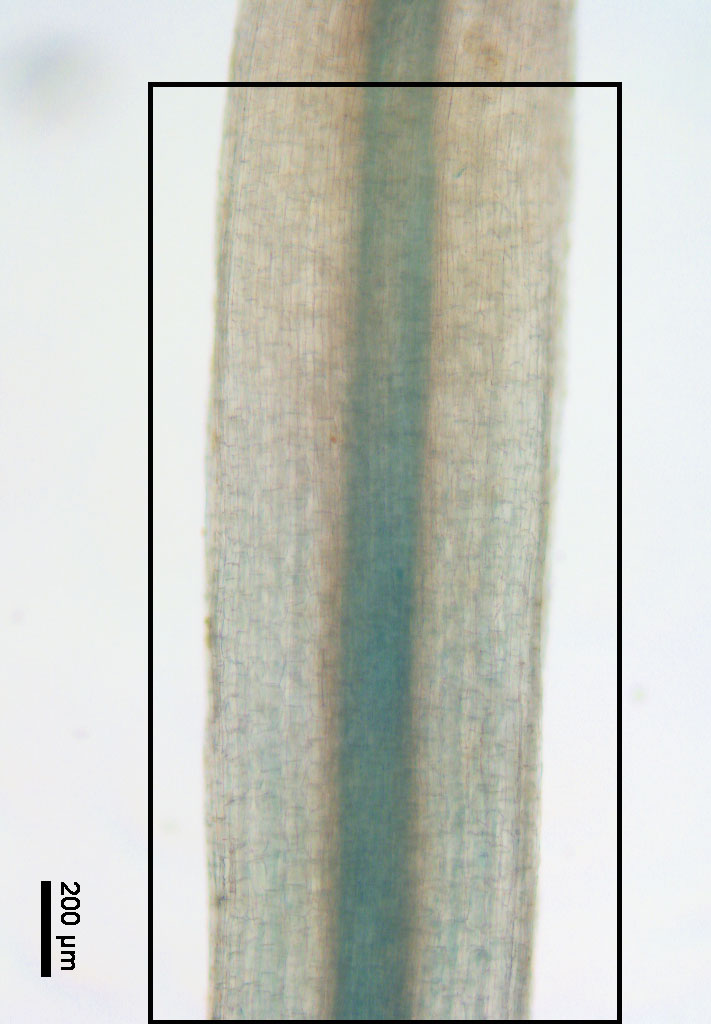

Supplement: Supplementary file 3 — Source data Fig. 1 [file 44318_2024_357_MOESM3_ESM.zip › Figure 1/Fig.1E/Control-2.jpg]

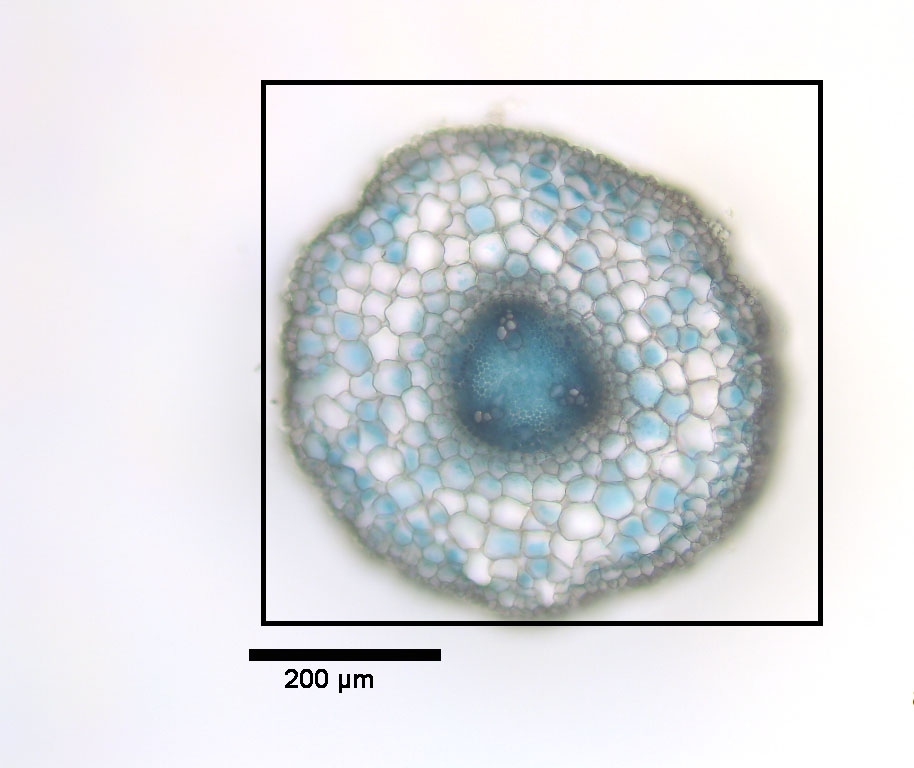

Supplement: Supplementary file 3 — Source data Fig. 1 [file 44318_2024_357_MOESM3_ESM.zip › Figure 1/Fig.1E/NaCl section.jpg]

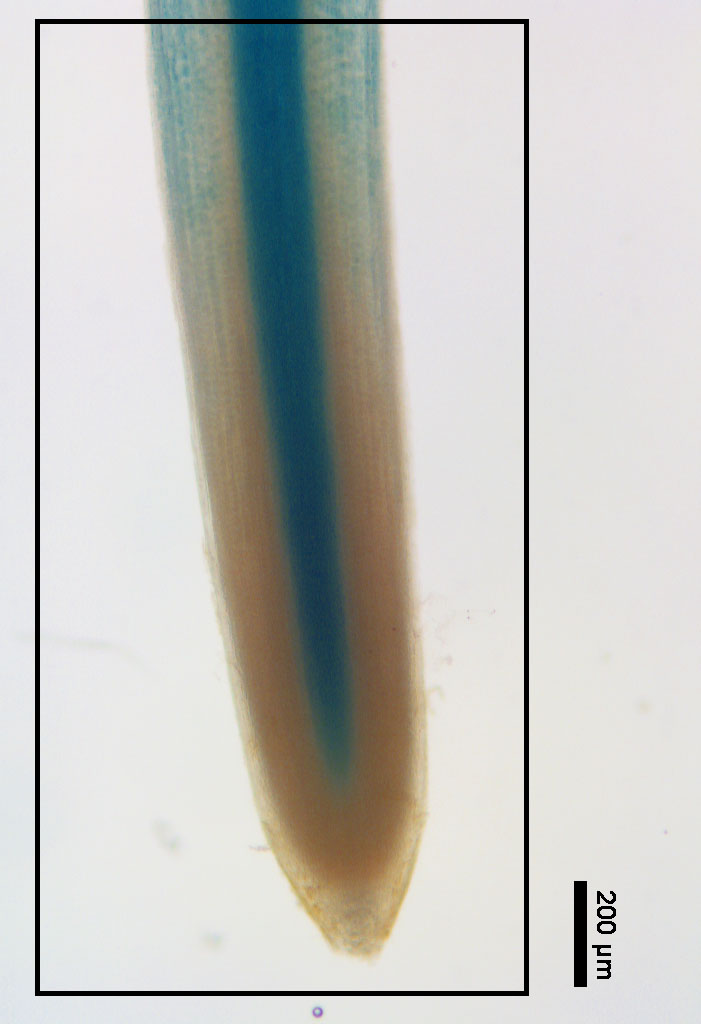

Supplement: Supplementary file 3 — Source data Fig. 1 [file 44318_2024_357_MOESM3_ESM.zip › Figure 1/Fig.1E/NaCl-1.jpg]

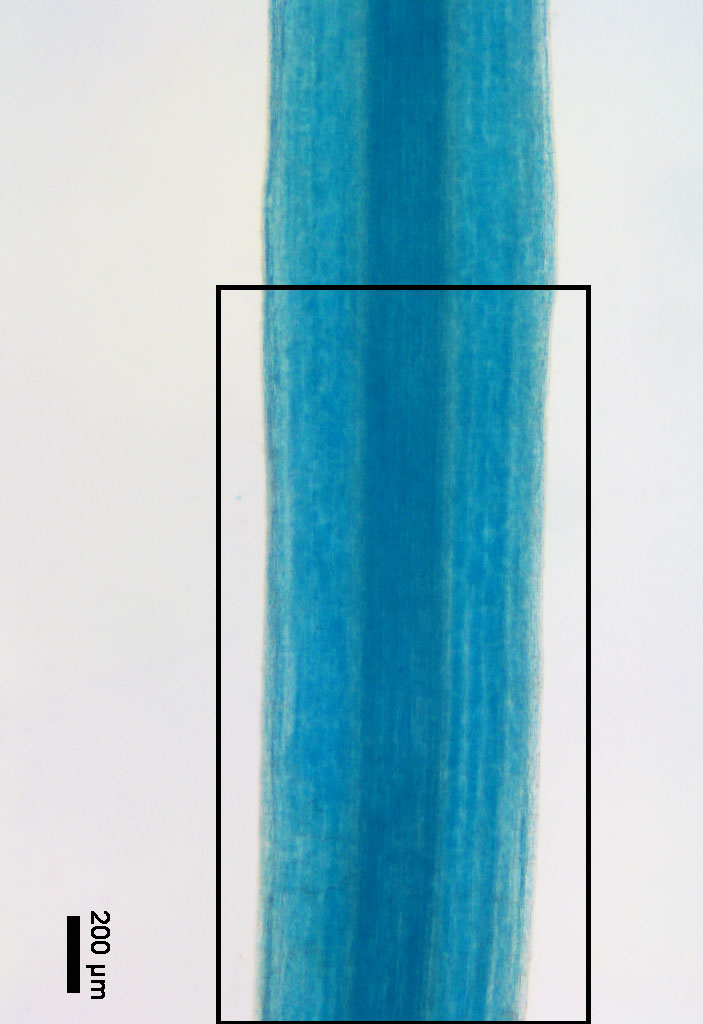

Supplement: Supplementary file 3 — Source data Fig. 1 [file 44318_2024_357_MOESM3_ESM.zip › Figure 1/Fig.1E/NaCl-2.jpg]

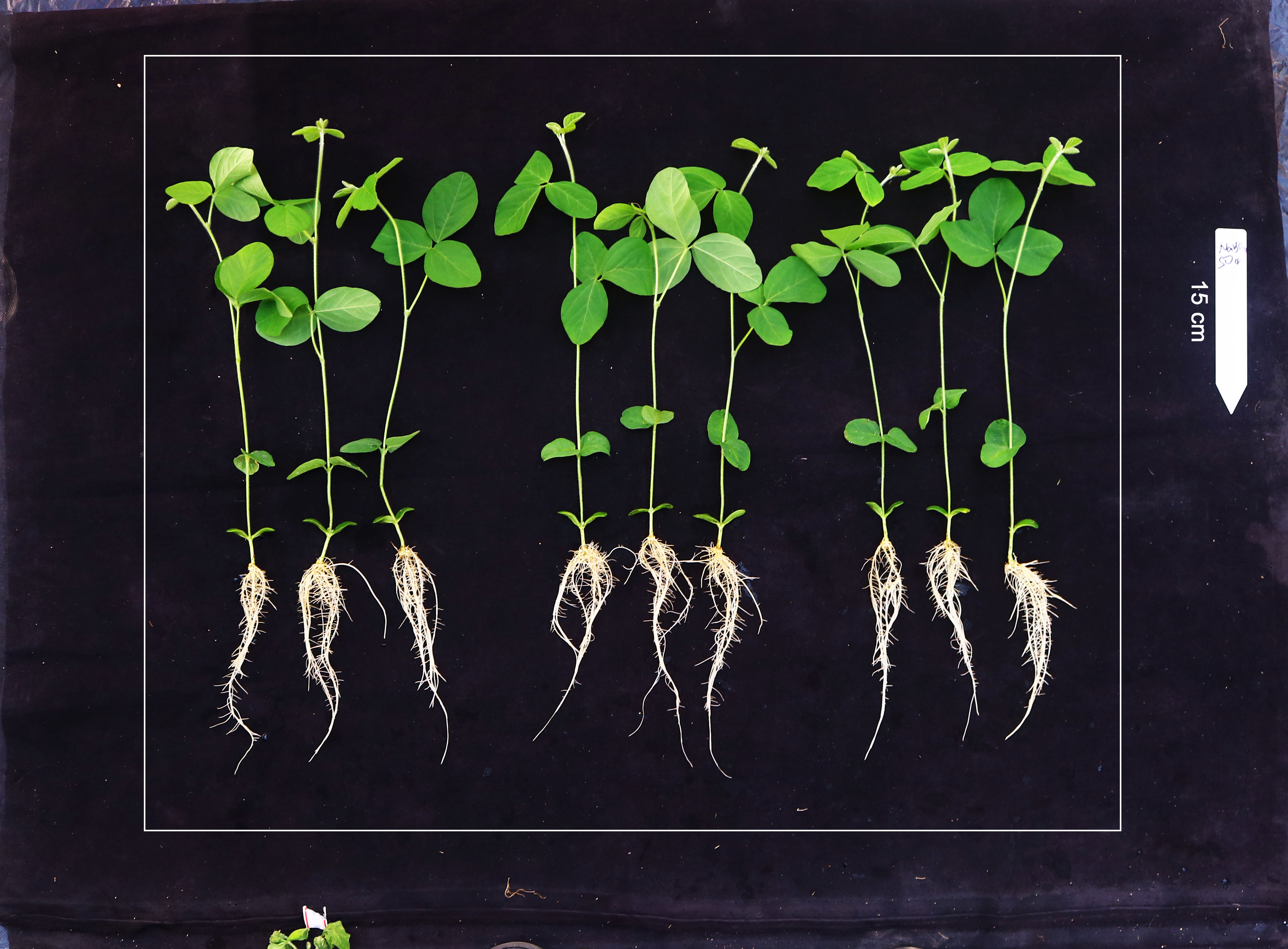

Supplement: Supplementary file 4 — Source data Fig. 2 [file 44318_2024_357_MOESM4_ESM.zip › Figure 2/Fig.2A/Control.jpg]

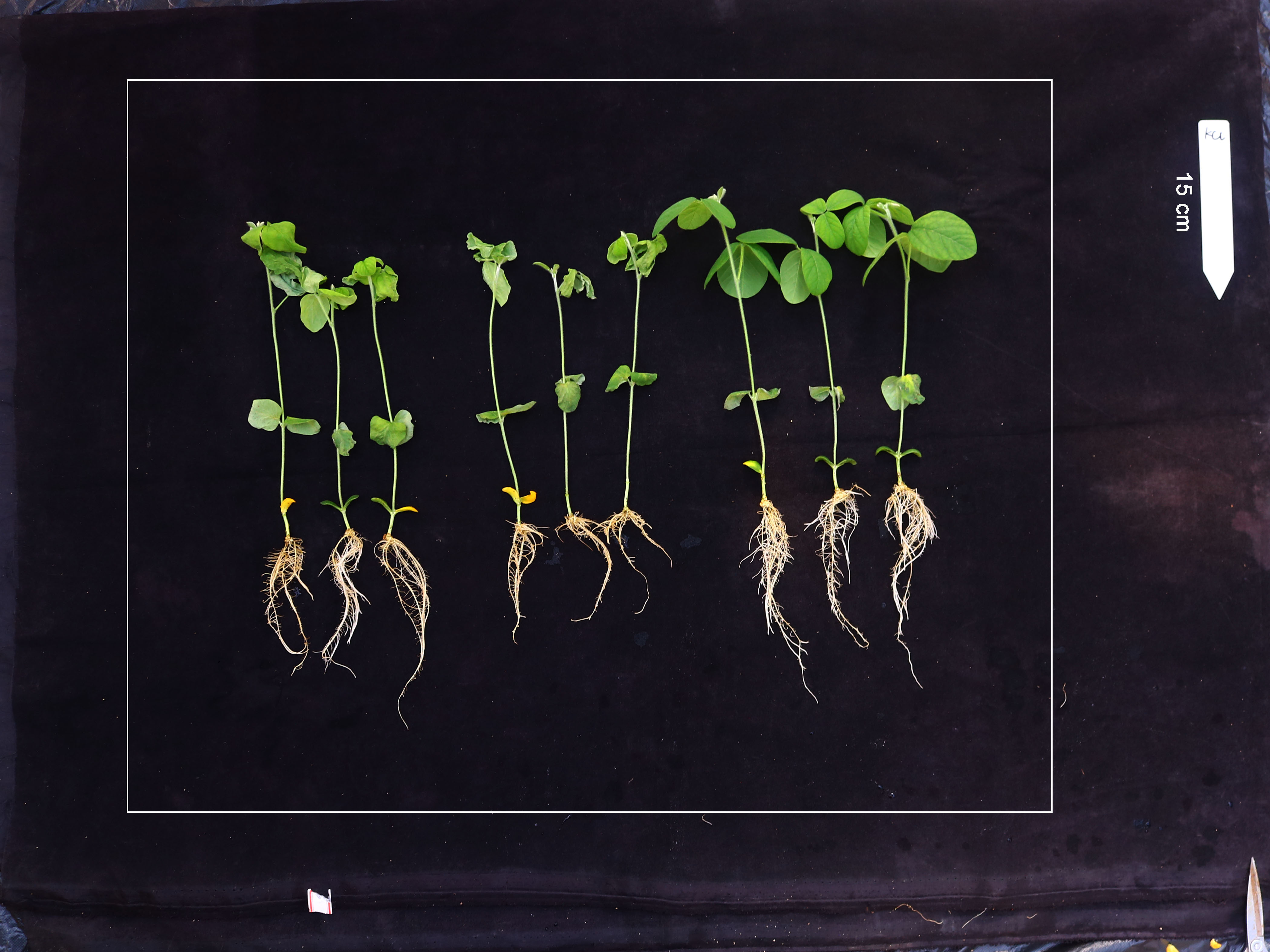

Supplement: Supplementary file 4 — Source data Fig. 2 [file 44318_2024_357_MOESM4_ESM.zip › Figure 2/Fig.2A/KCl.jpg]

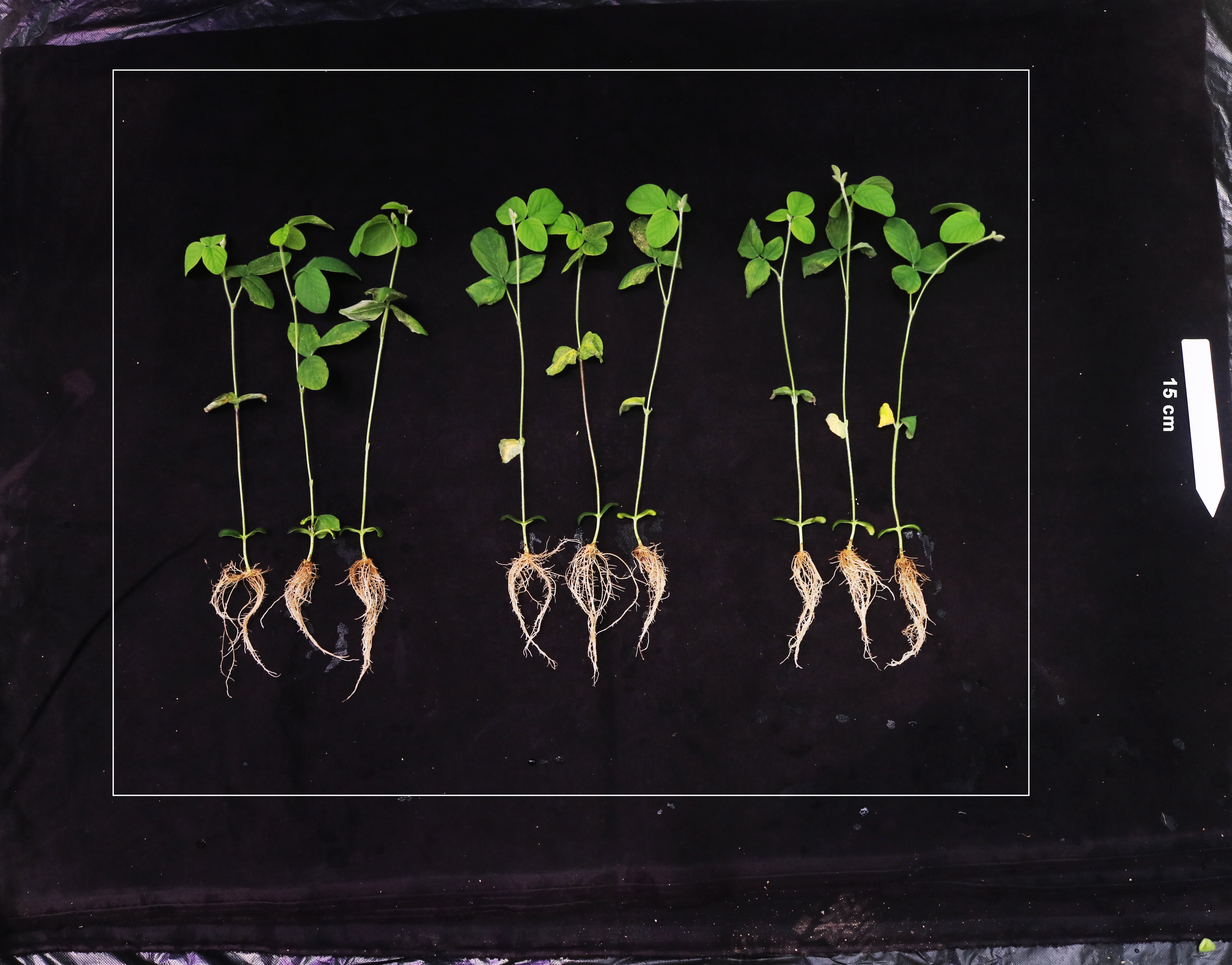

Supplement: Supplementary file 4 — Source data Fig. 2 [file 44318_2024_357_MOESM4_ESM.zip › Figure 2/Fig.2A/Na2SO4.jpg]

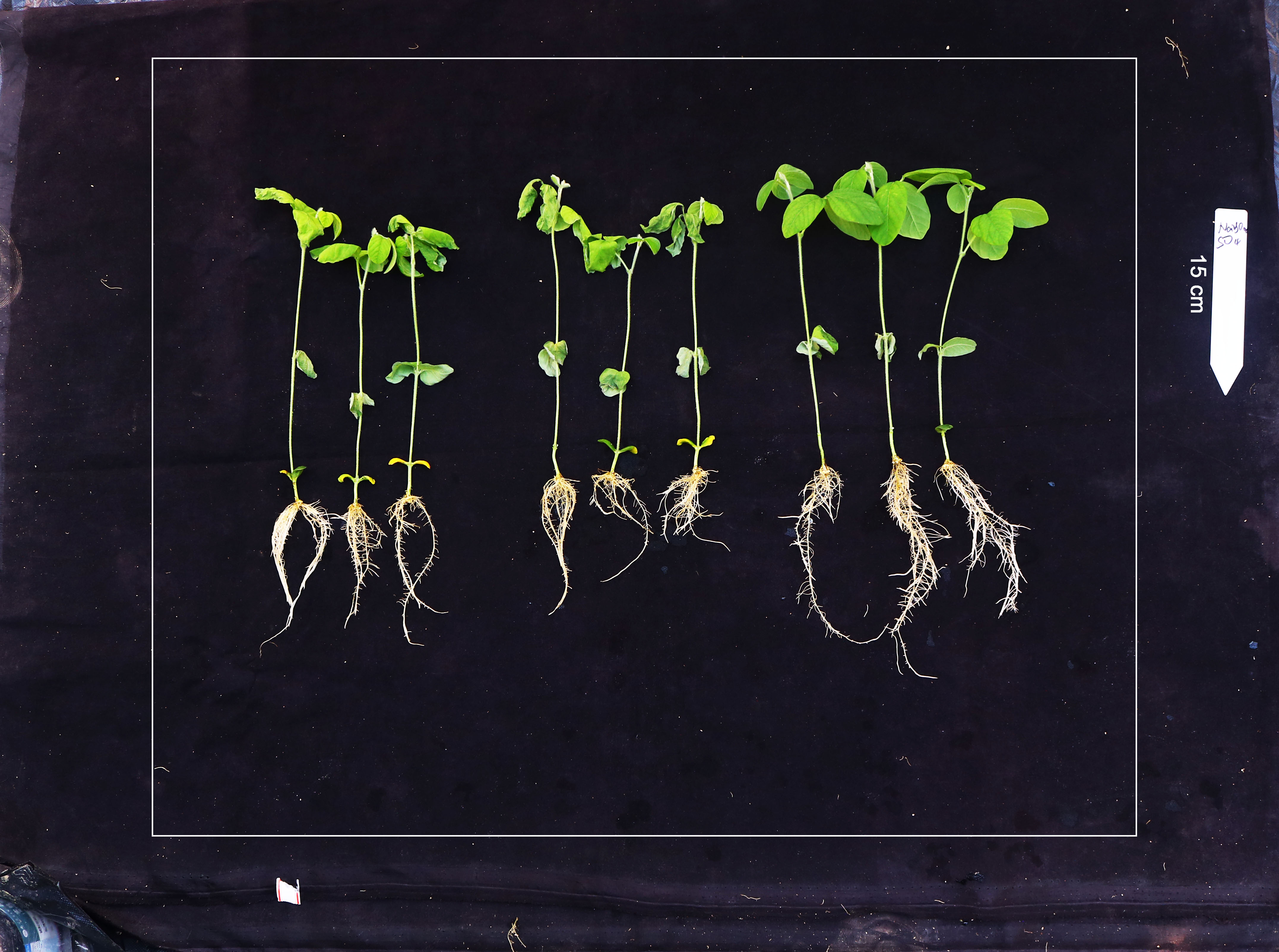

Supplement: Supplementary file 4 — Source data Fig. 2 [file 44318_2024_357_MOESM4_ESM.zip › Figure 2/Fig.2A/NaCl.jpg]

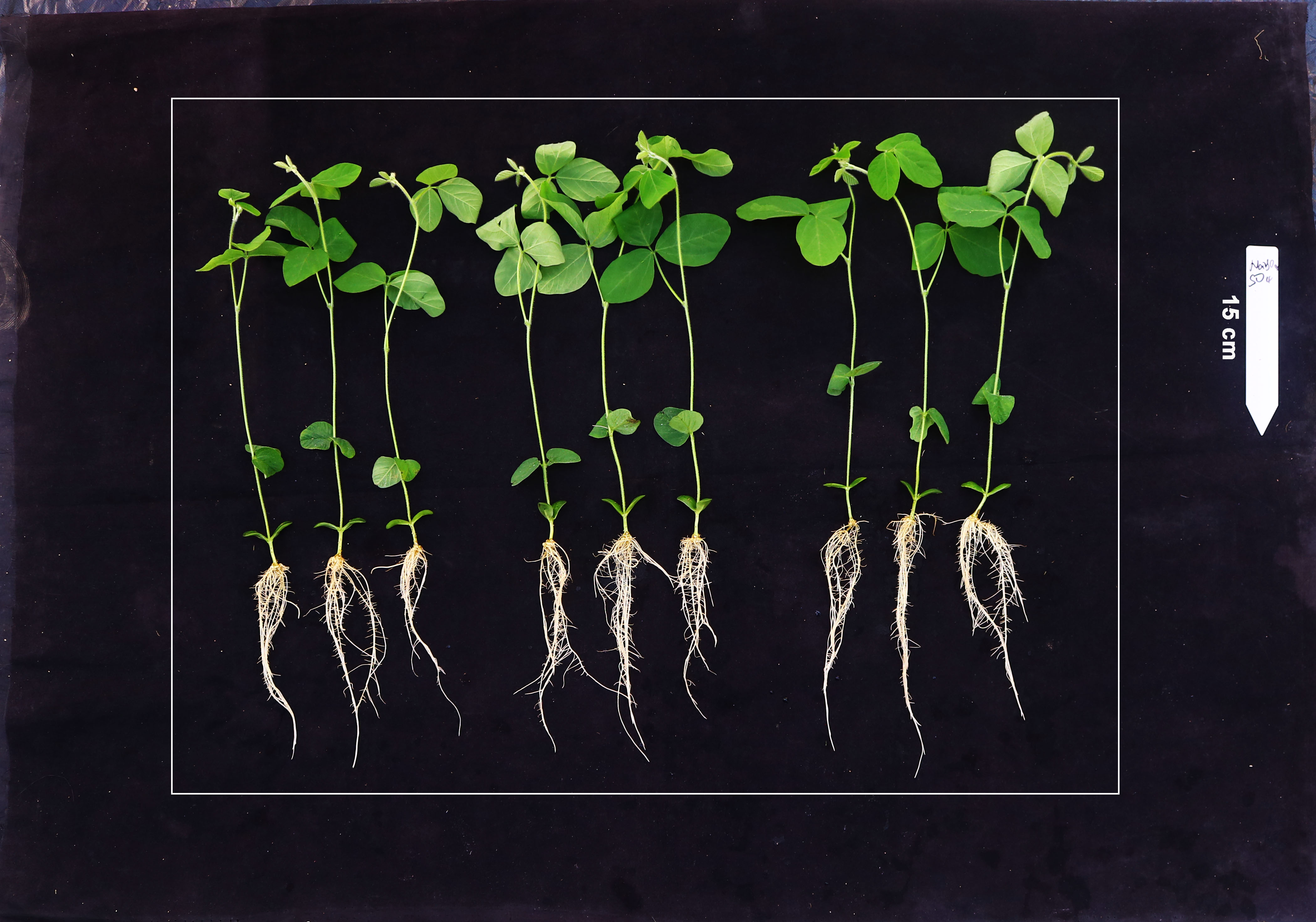

Supplement: Supplementary file 4 — Source data Fig. 2 [file 44318_2024_357_MOESM4_ESM.zip › Figure 2/Fig.2E/Control.jpg]

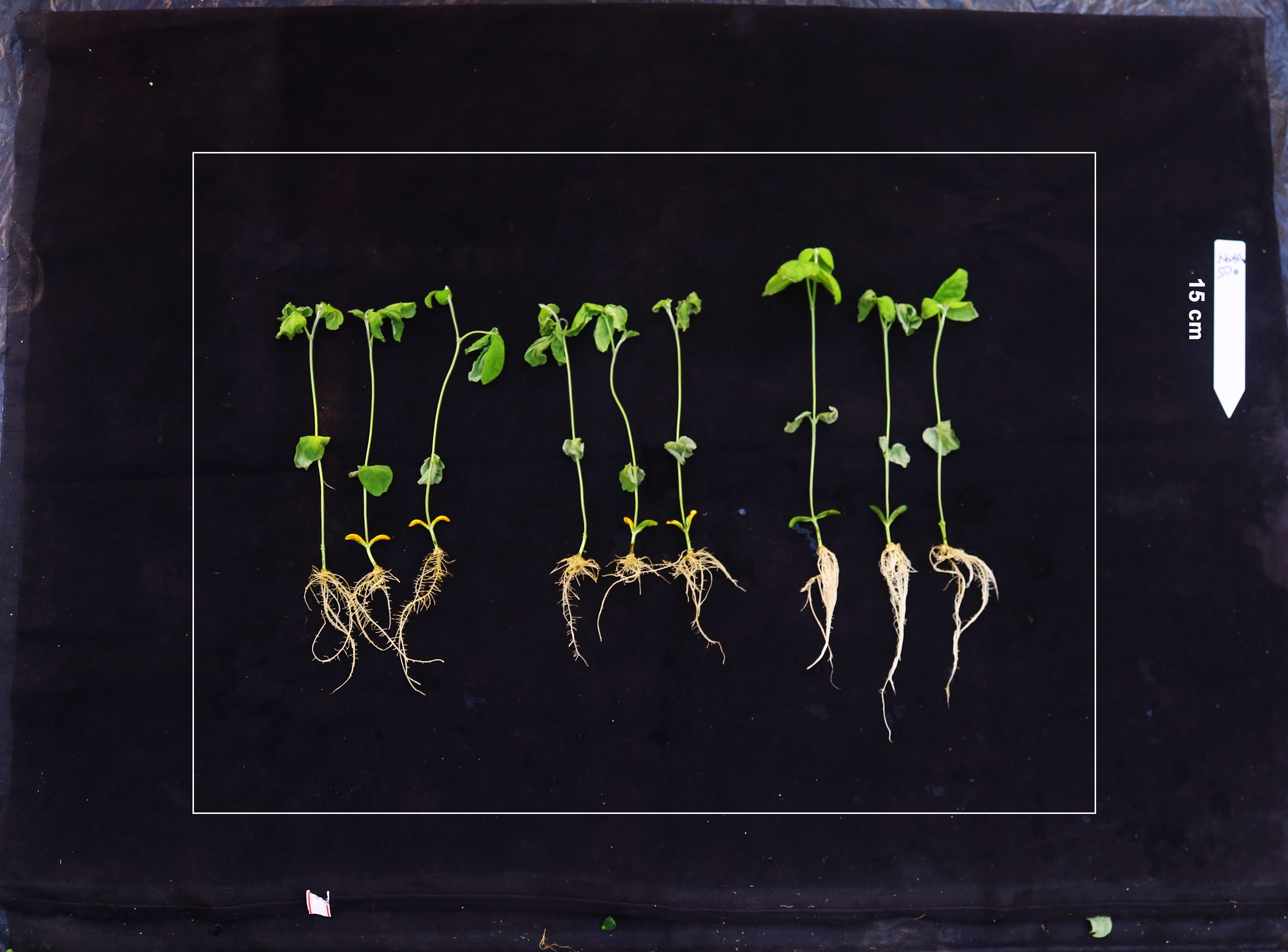

Supplement: Supplementary file 4 — Source data Fig. 2 [file 44318_2024_357_MOESM4_ESM.zip › Figure 2/Fig.2E/NaCl.jpg]

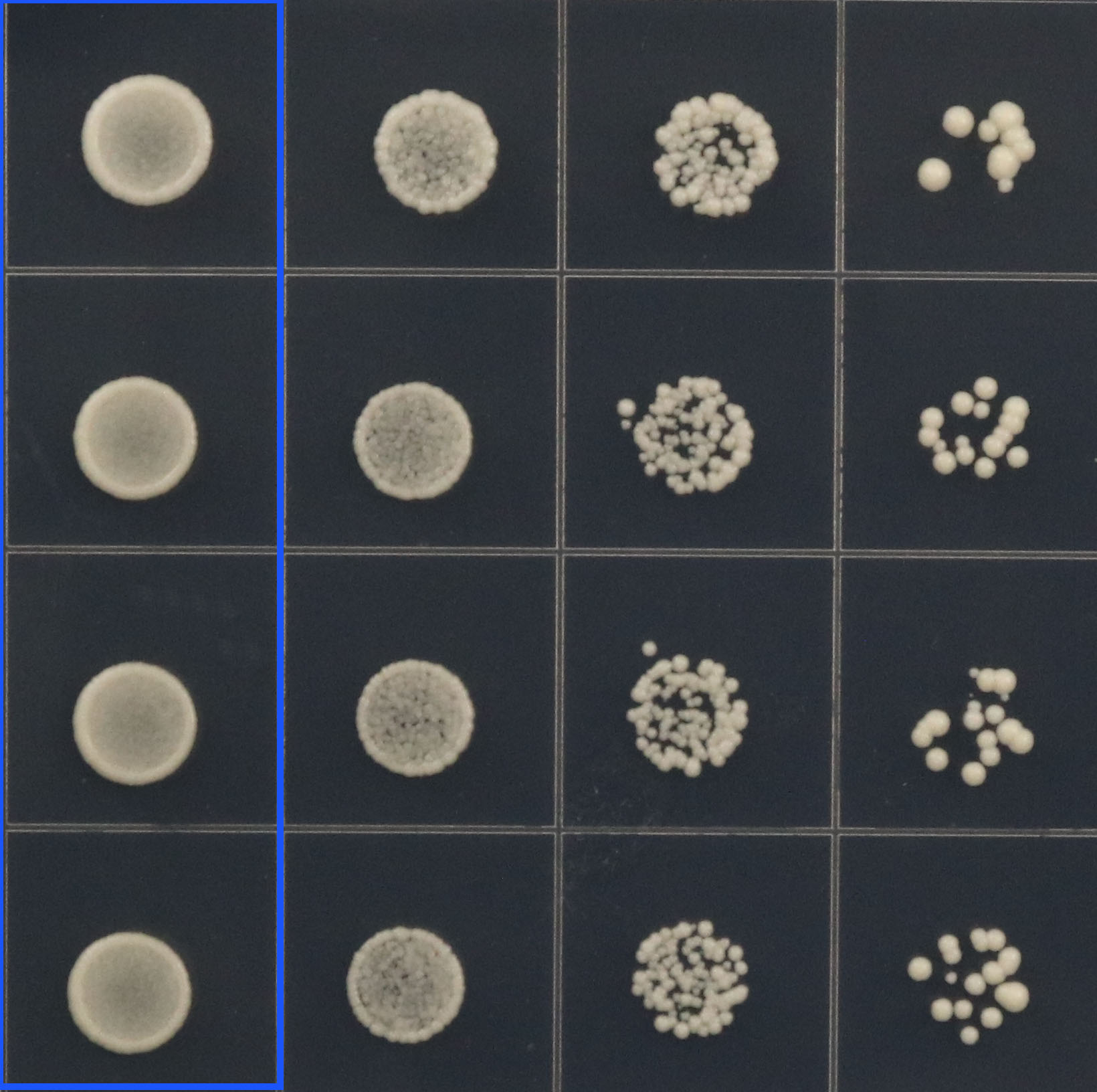

Supplement: Supplementary file 6 — Source data Fig. 4 [file 44318_2024_357_MOESM6_ESM.zip › Figure 4/Fig.4A/SD-LeuTrp.jpg]

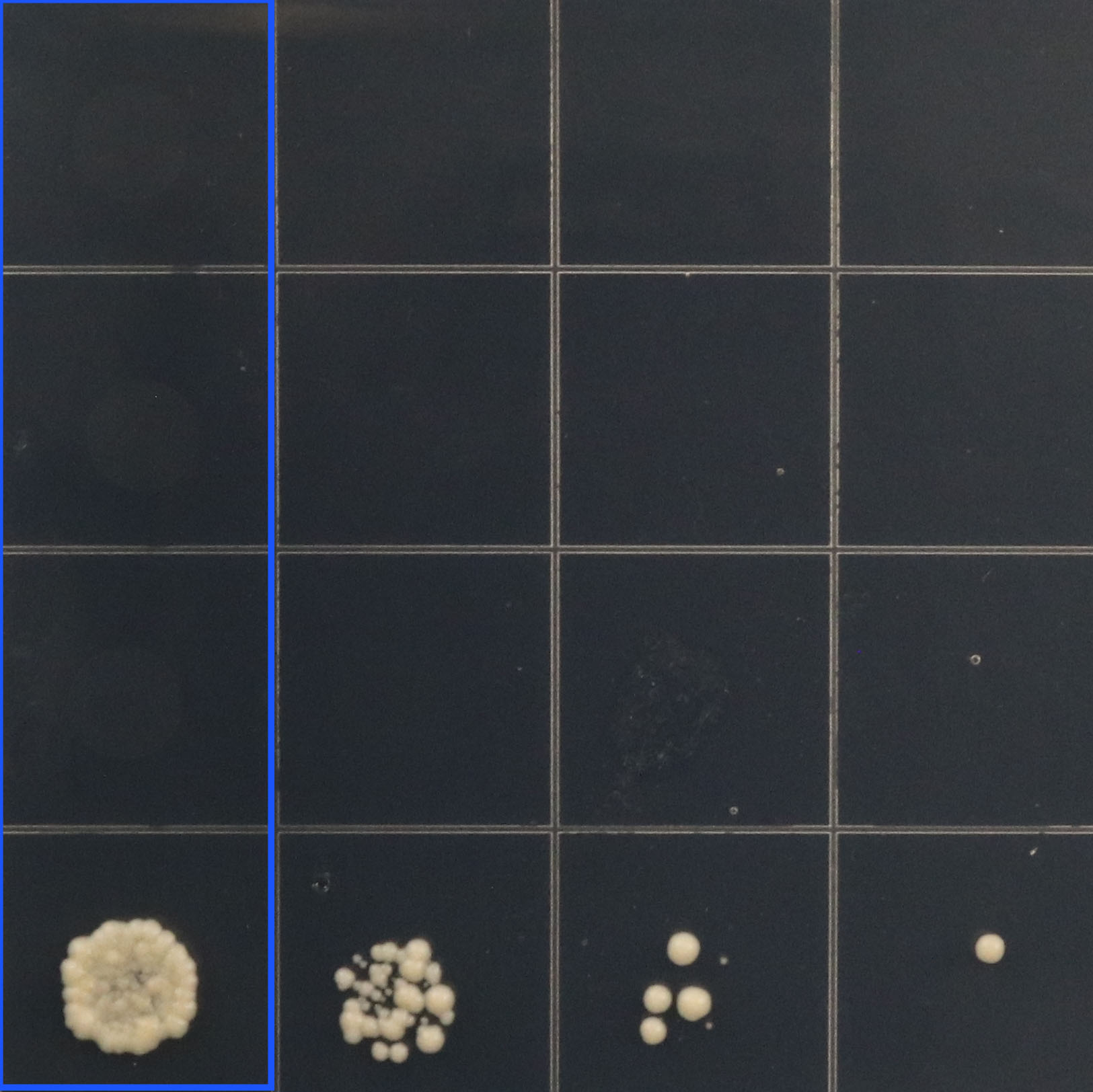

Supplement: Supplementary file 6 — Source data Fig. 4 [file 44318_2024_357_MOESM6_ESM.zip › Figure 4/Fig.4A/SD-LeuTrpHisAde.jpg]

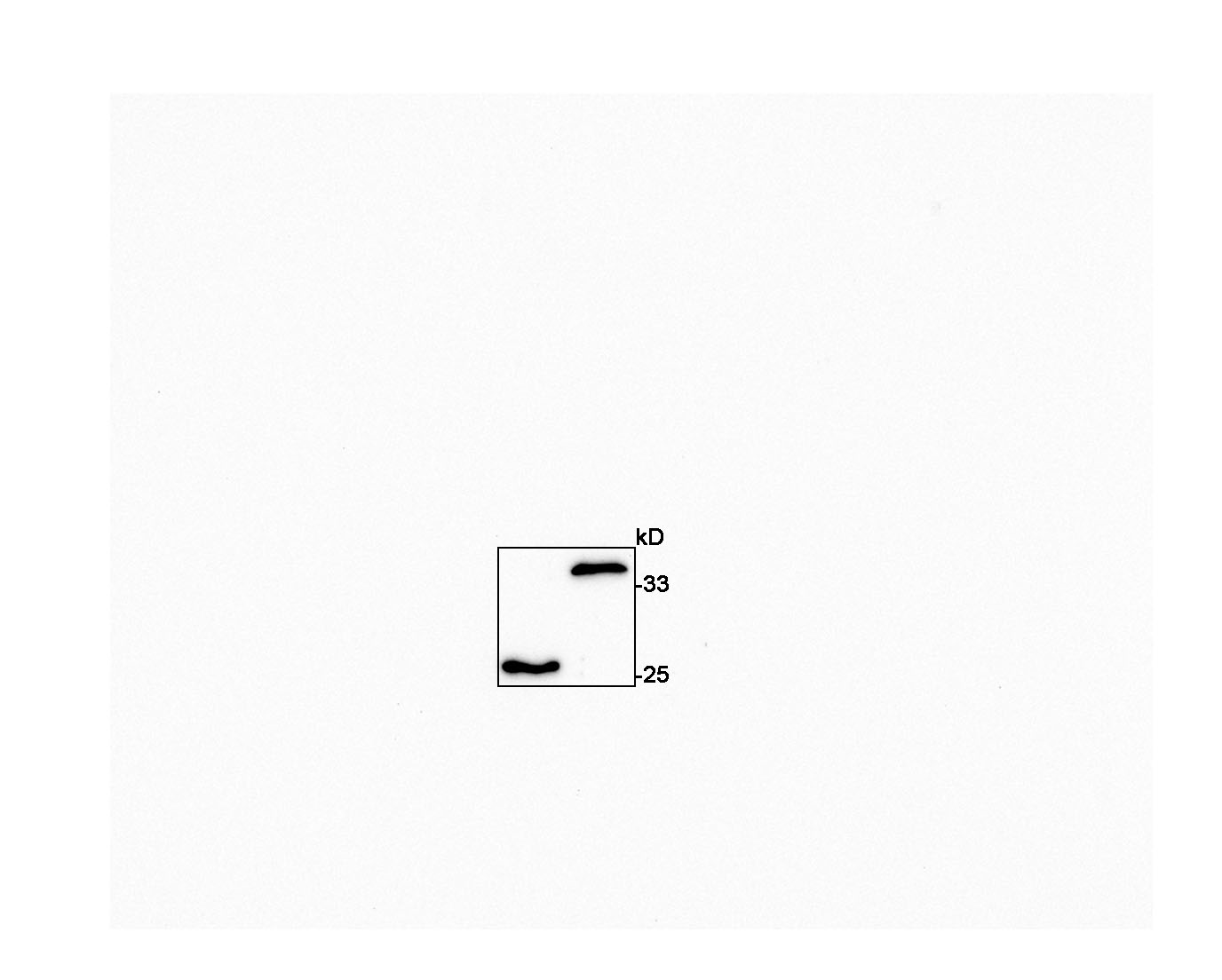

Supplement: Supplementary file 6 — Source data Fig. 4 [file 44318_2024_357_MOESM6_ESM.zip › Figure 4/Fig.4B/Input-Anti-GST.jpg]

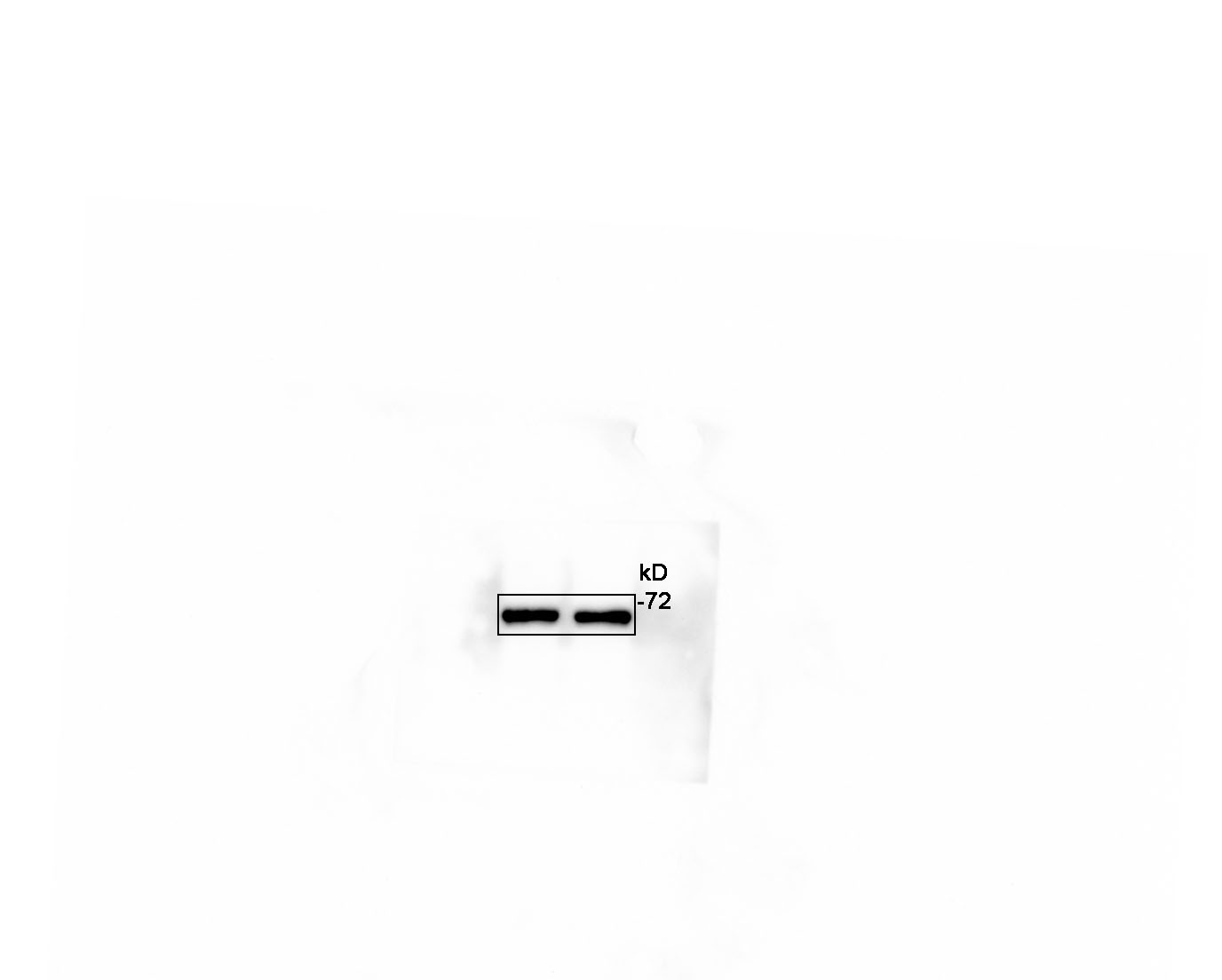

Supplement: Supplementary file 6 — Source data Fig. 4 [file 44318_2024_357_MOESM6_ESM.zip › Figure 4/Fig.4B/Input-Anti-His.jpg]

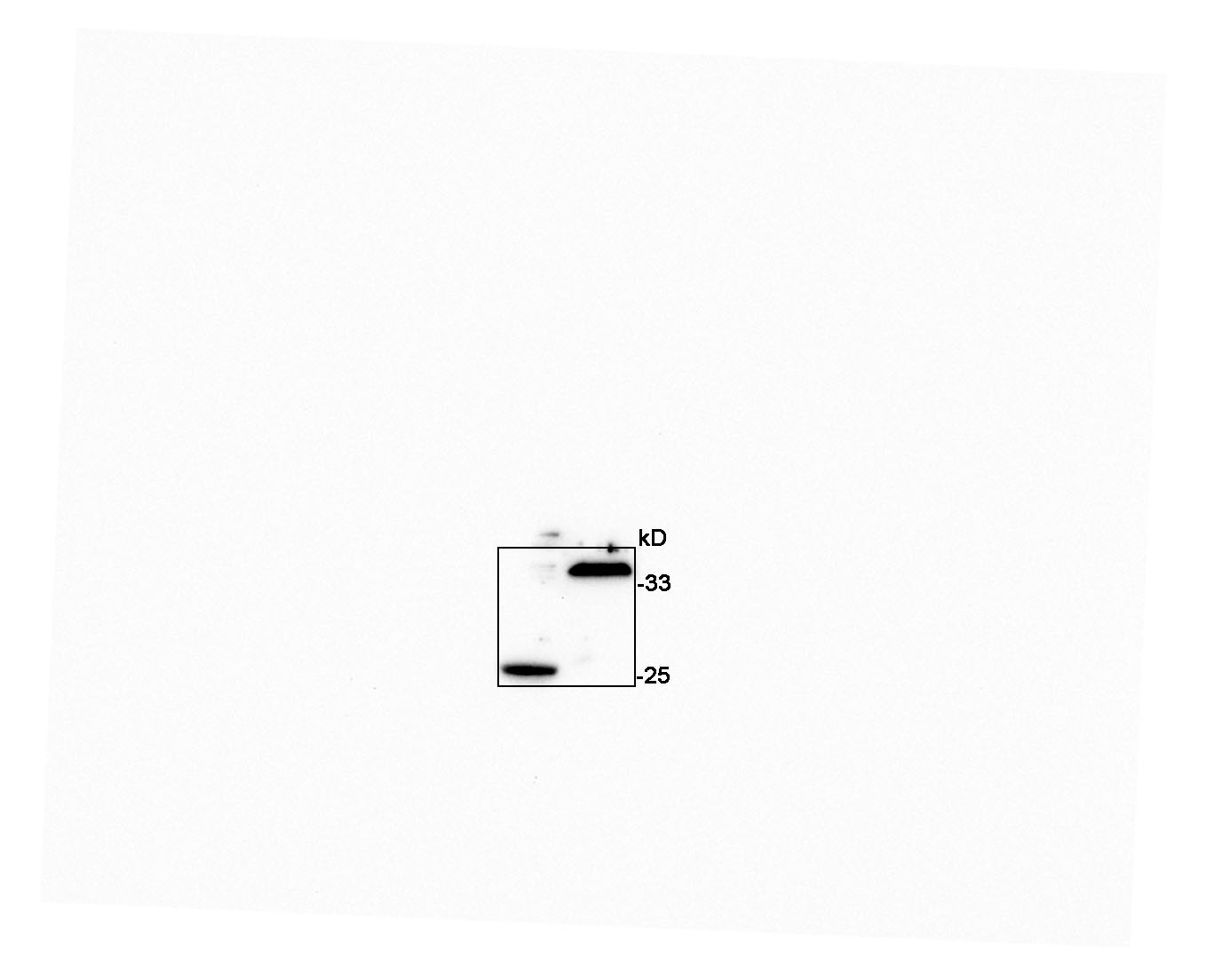

Supplement: Supplementary file 6 — Source data Fig. 4 [file 44318_2024_357_MOESM6_ESM.zip › Figure 4/Fig.4B/Pull down Anti-GST.jpg]

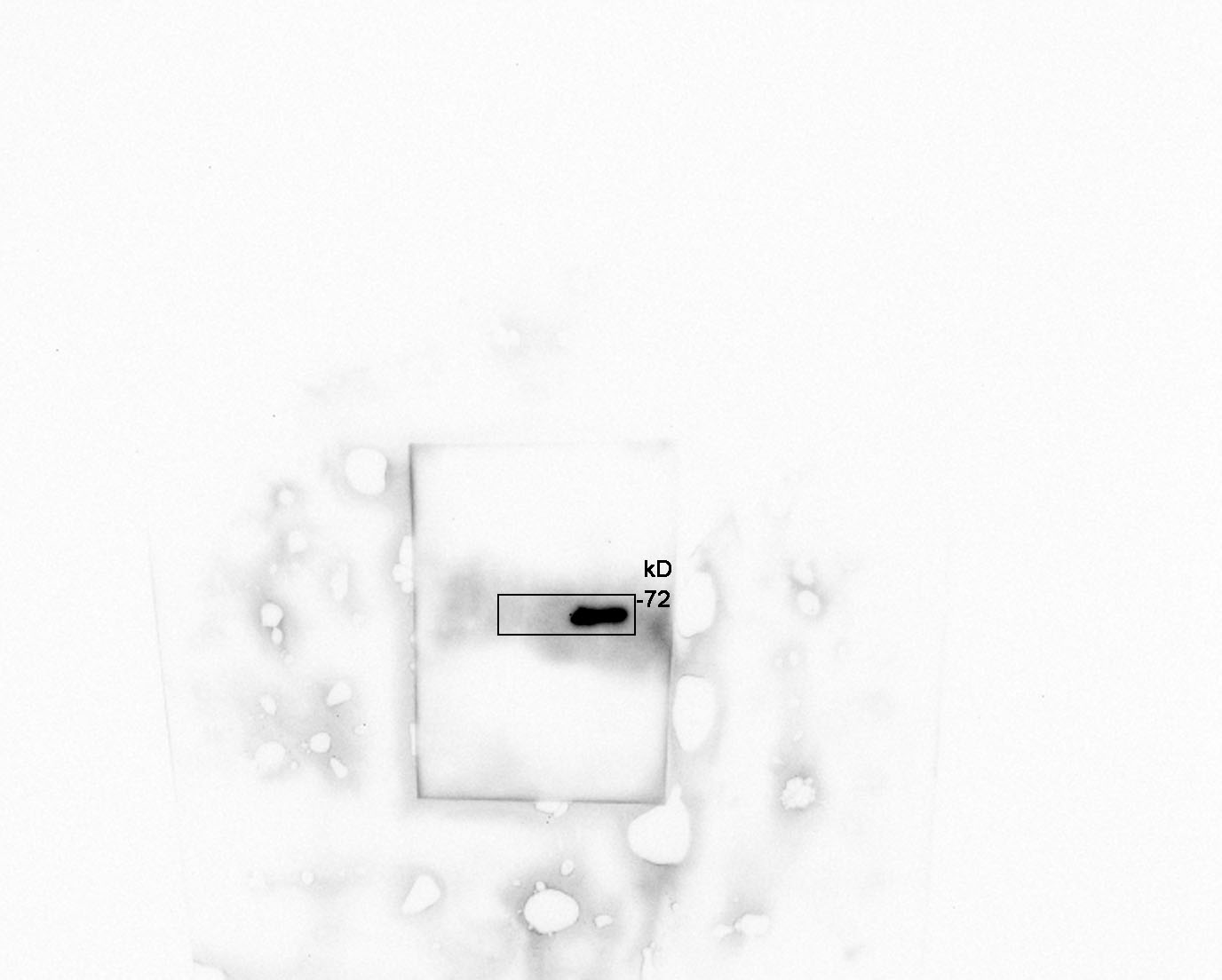

Supplement: Supplementary file 6 — Source data Fig. 4 [file 44318_2024_357_MOESM6_ESM.zip › Figure 4/Fig.4B/Pull down Anti-His.jpg]

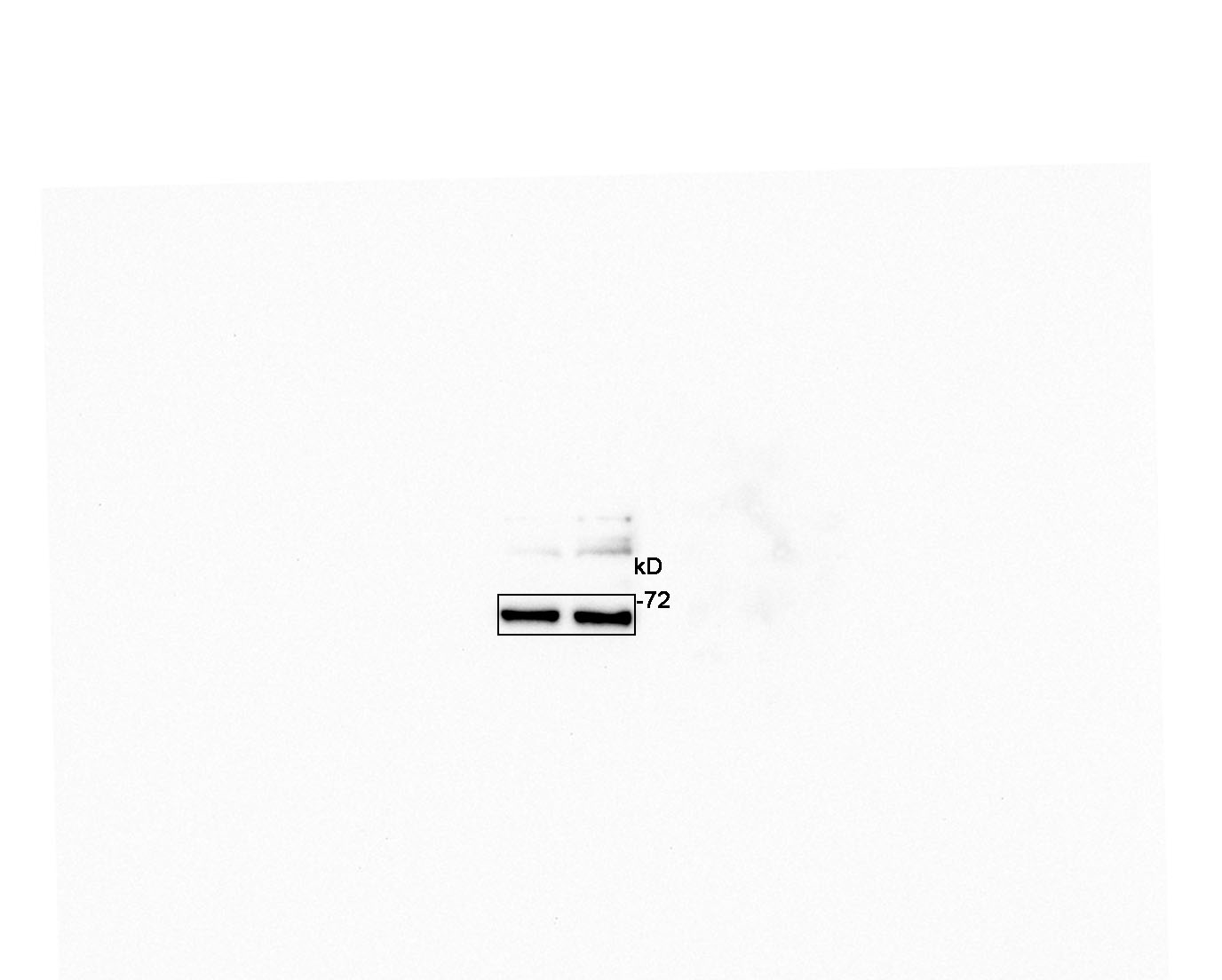

Supplement: Supplementary file 6 — Source data Fig. 4 [file 44318_2024_357_MOESM6_ESM.zip › Figure 4/Fig.4C/Input Anti-flag.jpg]

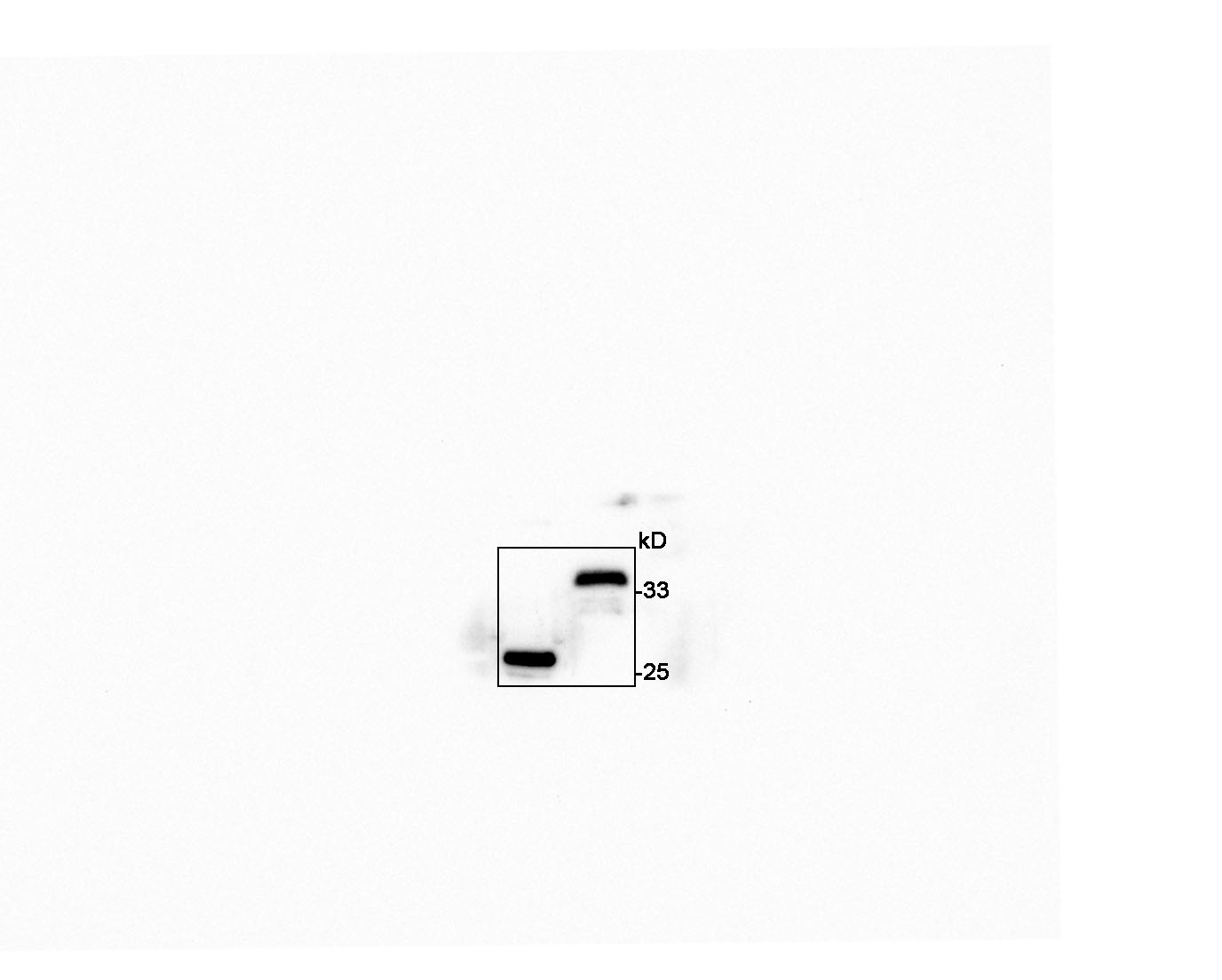

Supplement: Supplementary file 6 — Source data Fig. 4 [file 44318_2024_357_MOESM6_ESM.zip › Figure 4/Fig.4C/Input Anti-GFP.jpg]

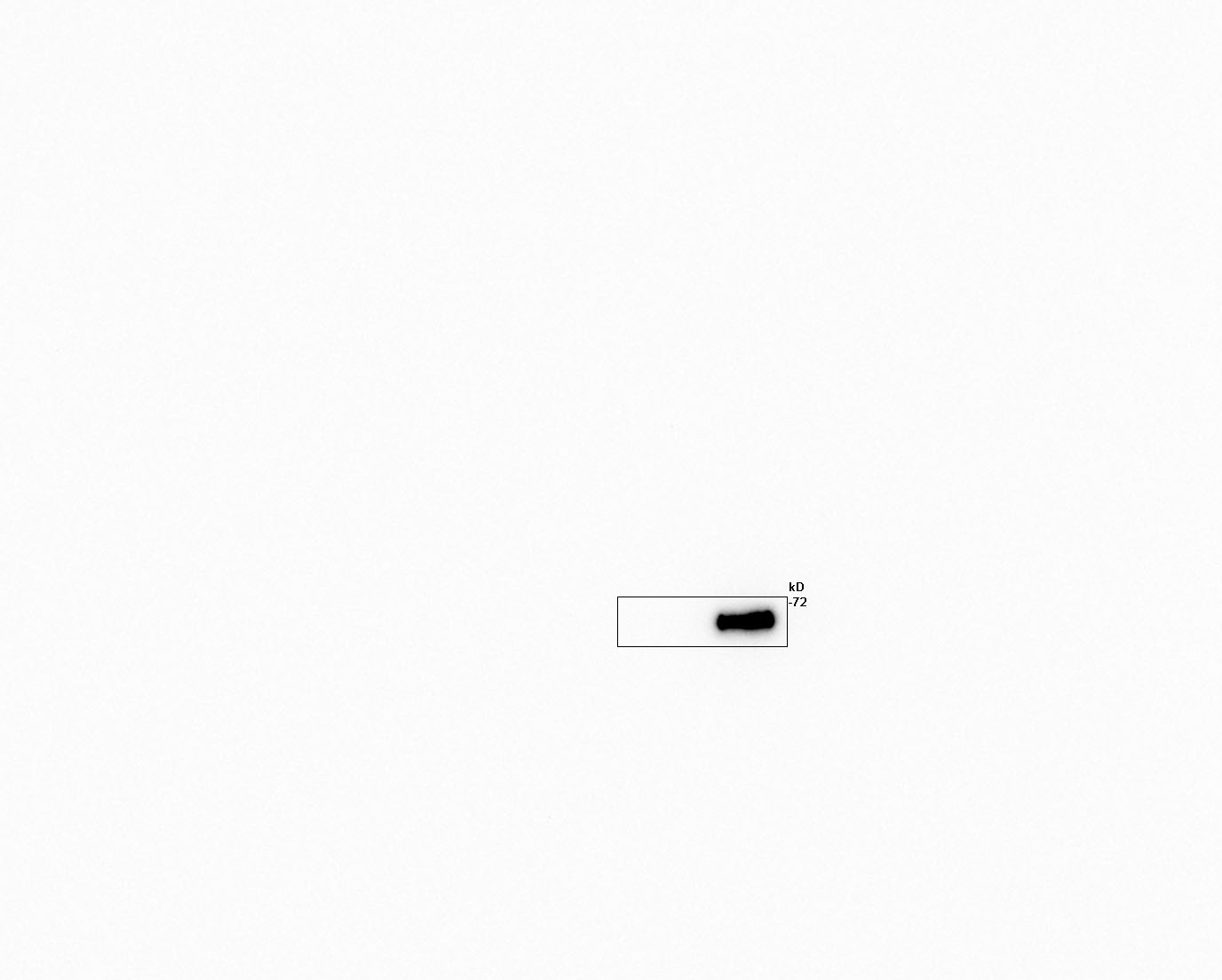

Supplement: Supplementary file 6 — Source data Fig. 4 [file 44318_2024_357_MOESM6_ESM.zip › Figure 4/Fig.4C/IP Anti-flag.jpg]

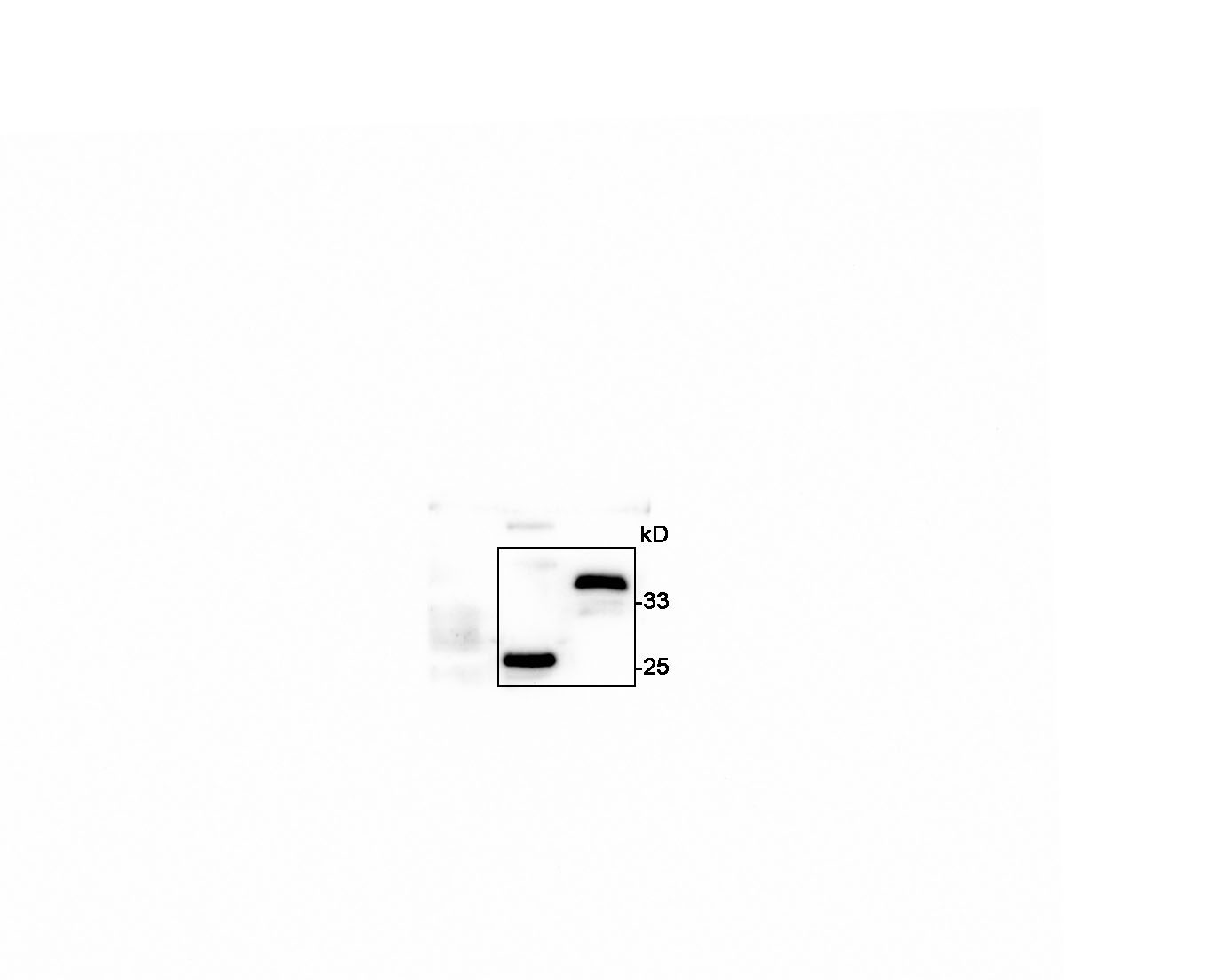

Supplement: Supplementary file 6 — Source data Fig. 4 [file 44318_2024_357_MOESM6_ESM.zip › Figure 4/Fig.4C/IP Anti-GFP.jpg]

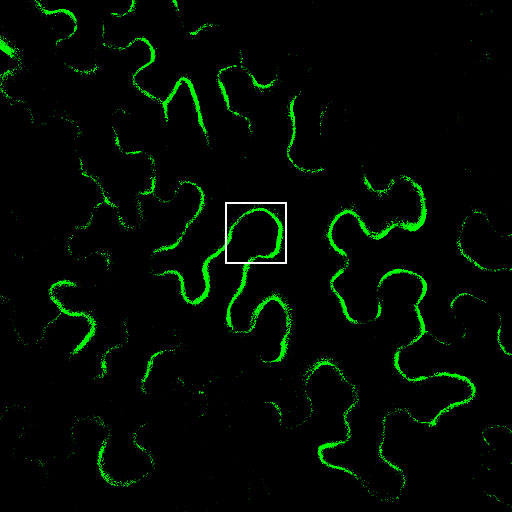

Supplement: Supplementary file 6 — Source data Fig. 4 [file 44318_2024_357_MOESM6_ESM.zip › Figure 4/Fig.4D/Control/GFP---GmNPF7.5 GFP-AtCBL1n mCherry.jpg]

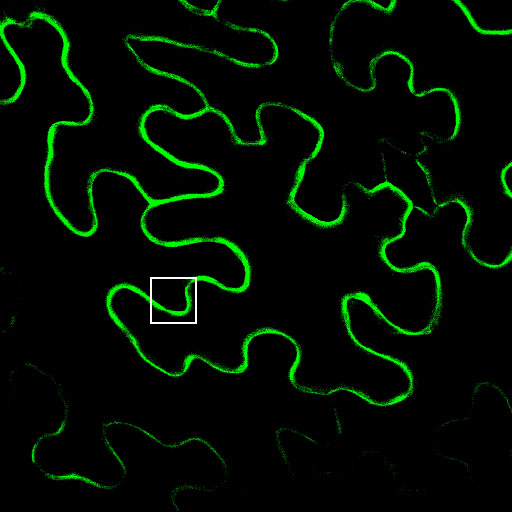

Supplement: Supplementary file 6 — Source data Fig. 4 [file 44318_2024_357_MOESM6_ESM.zip › Figure 4/Fig.4D/Control/GFP---GmNPF7.5 GFP-GmPI4Kγ4 mCherry.jpg]

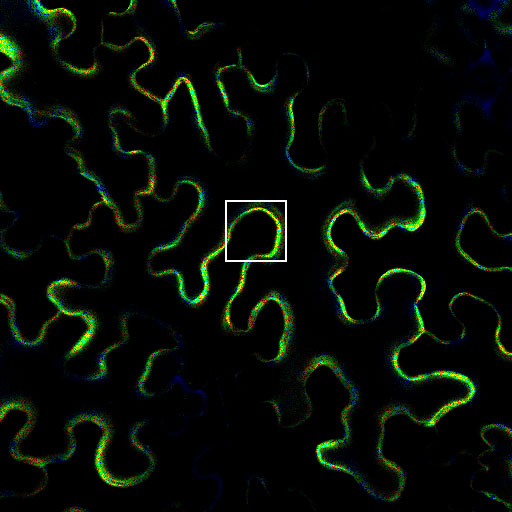

Supplement: Supplementary file 6 — Source data Fig. 4 [file 44318_2024_357_MOESM6_ESM.zip › Figure 4/Fig.4D/Control/Lifetime---GmNPF7.5 GFP-AtCBL1n mCherry.jpg]

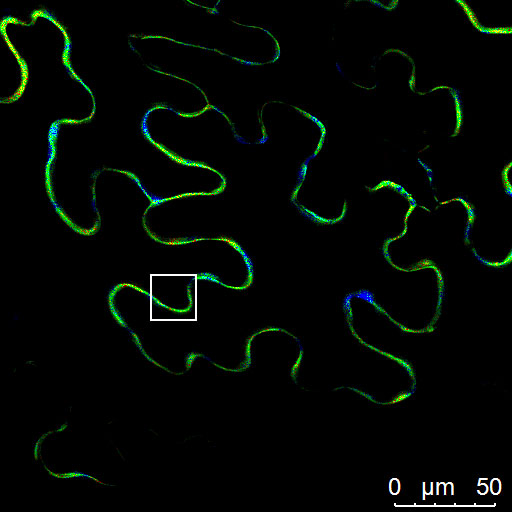

Supplement: Supplementary file 6 — Source data Fig. 4 [file 44318_2024_357_MOESM6_ESM.zip › Figure 4/Fig.4D/Control/Lifetime---GmNPF7.5 GFP-GmPI4Kγ4 mCherry.jpg]

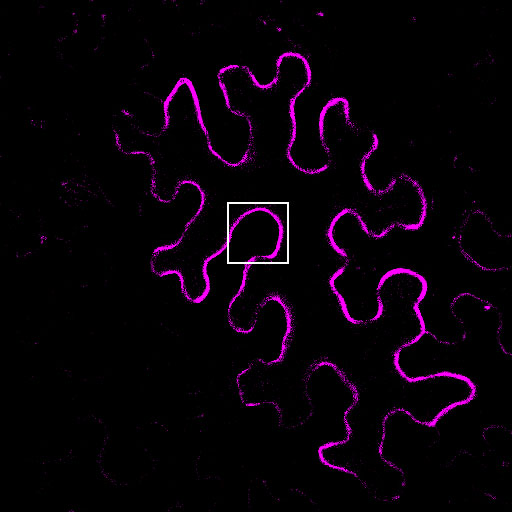

Supplement: Supplementary file 6 — Source data Fig. 4 [file 44318_2024_357_MOESM6_ESM.zip › Figure 4/Fig.4D/Control/mCherry---GmNPF7.5 GFP-AtCBL1n mCherry.jpg]

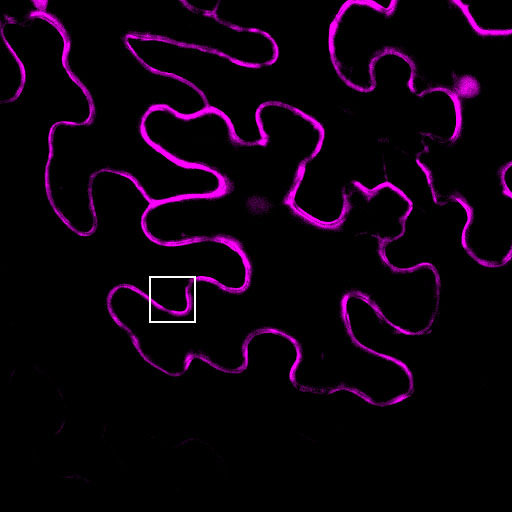

Supplement: Supplementary file 6 — Source data Fig. 4 [file 44318_2024_357_MOESM6_ESM.zip › Figure 4/Fig.4D/Control/mCherry---GmNPF7.5 GFP-GmPI4Kγ4 mCherry.jpg]

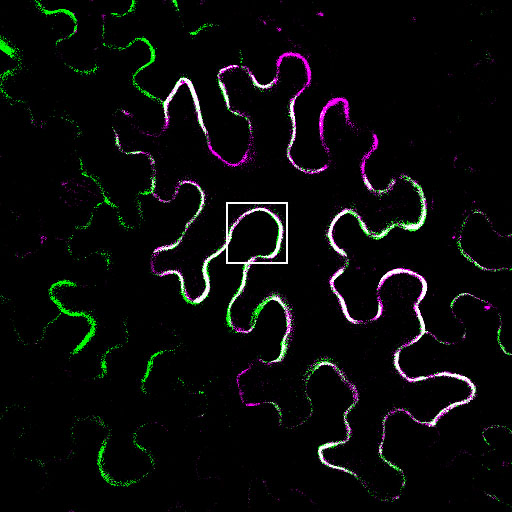

Supplement: Supplementary file 6 — Source data Fig. 4 [file 44318_2024_357_MOESM6_ESM.zip › Figure 4/Fig.4D/Control/Merge---GmNPF7.5 GFP-AtCBL1n mCherry.jpg]

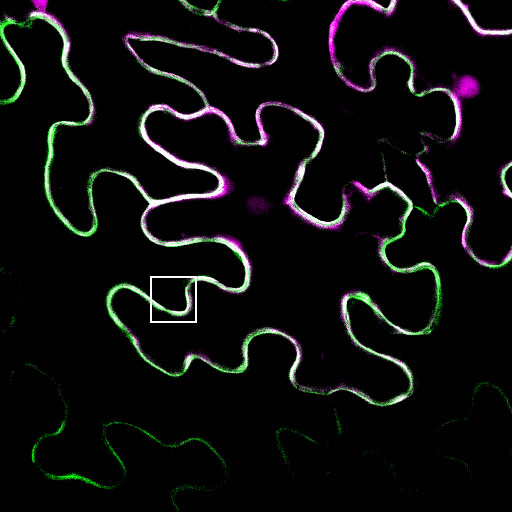

Supplement: Supplementary file 6 — Source data Fig. 4 [file 44318_2024_357_MOESM6_ESM.zip › Figure 4/Fig.4D/Control/Merge---GmNPF7.5 GFP-GmPI4Kγ4 mCherry.jpg]

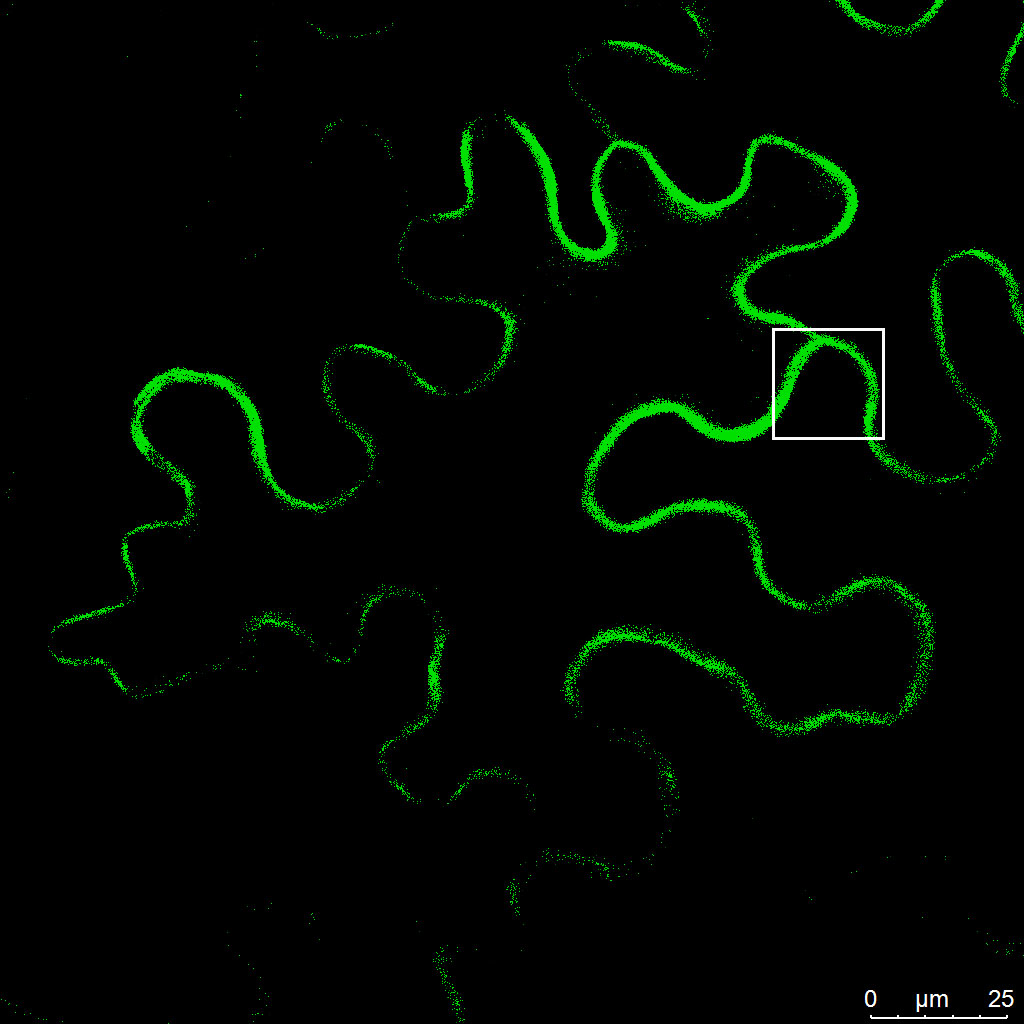

Supplement: Supplementary file 6 — Source data Fig. 4 [file 44318_2024_357_MOESM6_ESM.zip › Figure 4/Fig.4D/NaCl/GFP---GmNPF7.5 GFP-AtCBL1n mCherry.jpg]

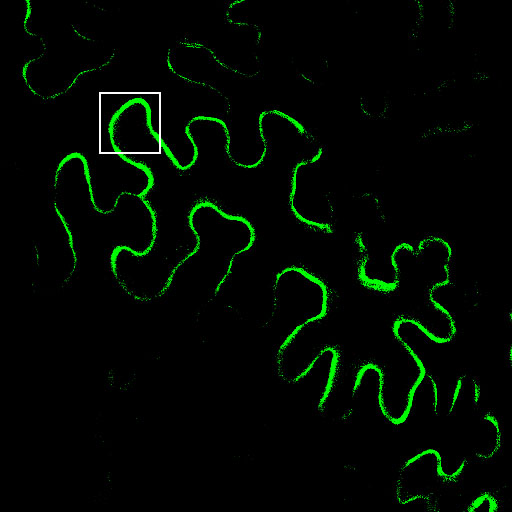

Supplement: Supplementary file 6 — Source data Fig. 4 [file 44318_2024_357_MOESM6_ESM.zip › Figure 4/Fig.4D/NaCl/GFP---GmNPF7.5 GFP-GmPI4Kγ4 mCherry.jpg]

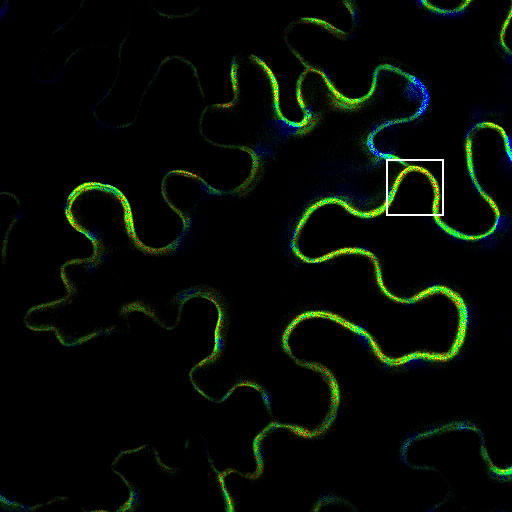

Supplement: Supplementary file 6 — Source data Fig. 4 [file 44318_2024_357_MOESM6_ESM.zip › Figure 4/Fig.4D/NaCl/Lifetime---GmNPF7.5 GFP-AtCBL1n mCherry.jpg]

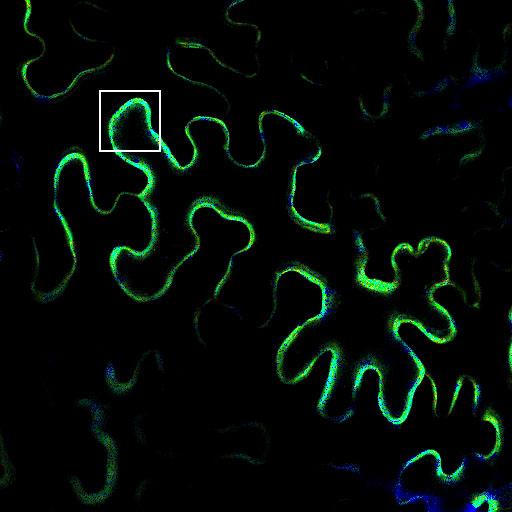

Supplement: Supplementary file 6 — Source data Fig. 4 [file 44318_2024_357_MOESM6_ESM.zip › Figure 4/Fig.4D/NaCl/Lifetime---GmNPF7.5 GFP-GmPI4Kγ4 mCherry.jpg]

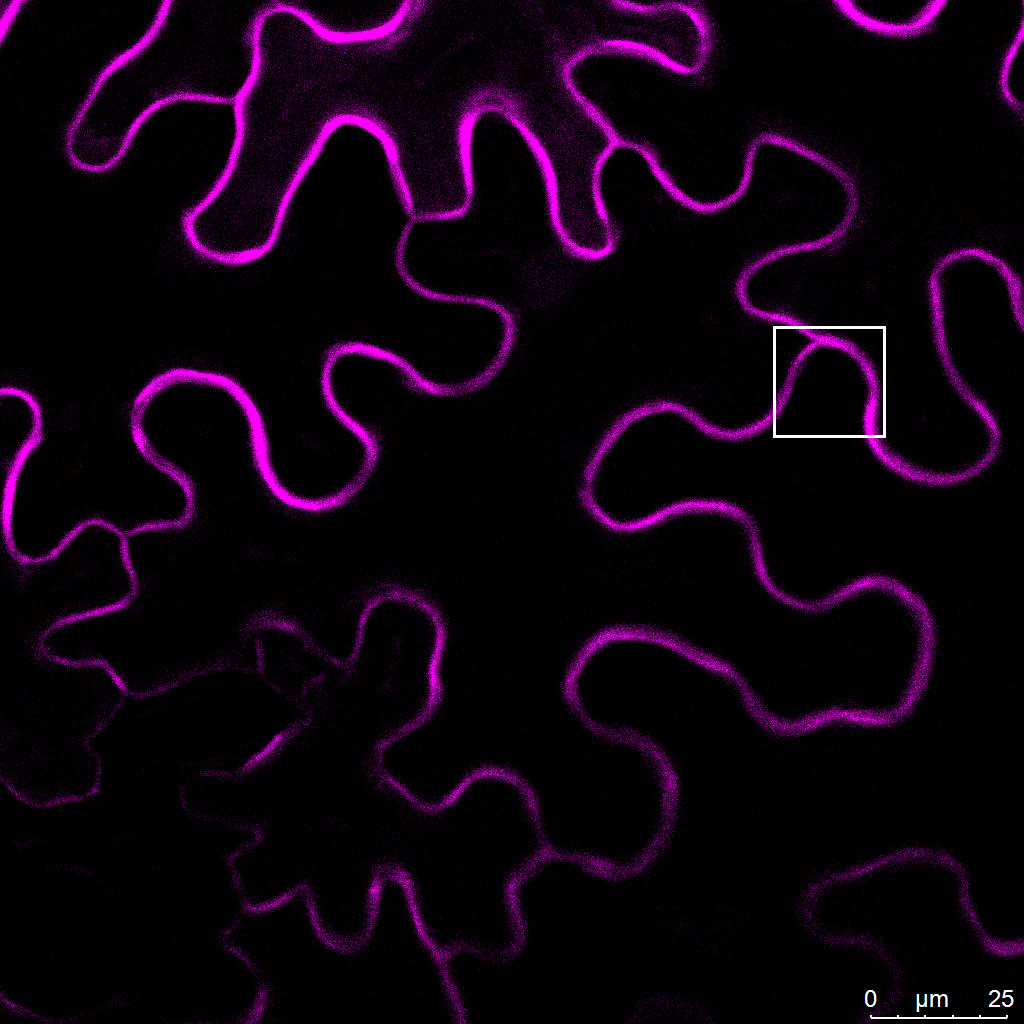

Supplement: Supplementary file 6 — Source data Fig. 4 [file 44318_2024_357_MOESM6_ESM.zip › Figure 4/Fig.4D/NaCl/Mcherry---GmNPF7.5 GFP-AtCBL1n mCherry.jpg]

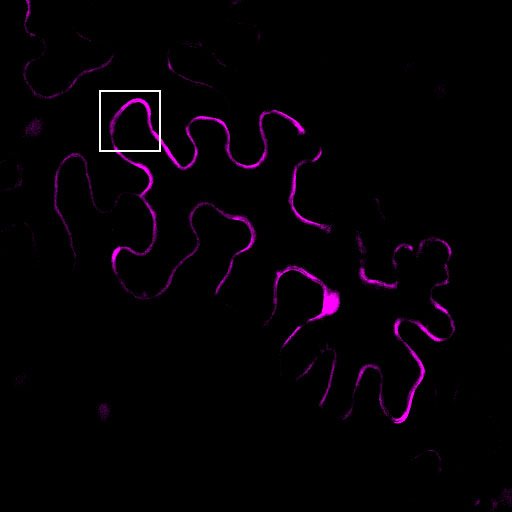

Supplement: Supplementary file 6 — Source data Fig. 4 [file 44318_2024_357_MOESM6_ESM.zip › Figure 4/Fig.4D/NaCl/Mcherry---GmNPF7.5 GFP-GmPI4Kγ4 mCherry.jpg]

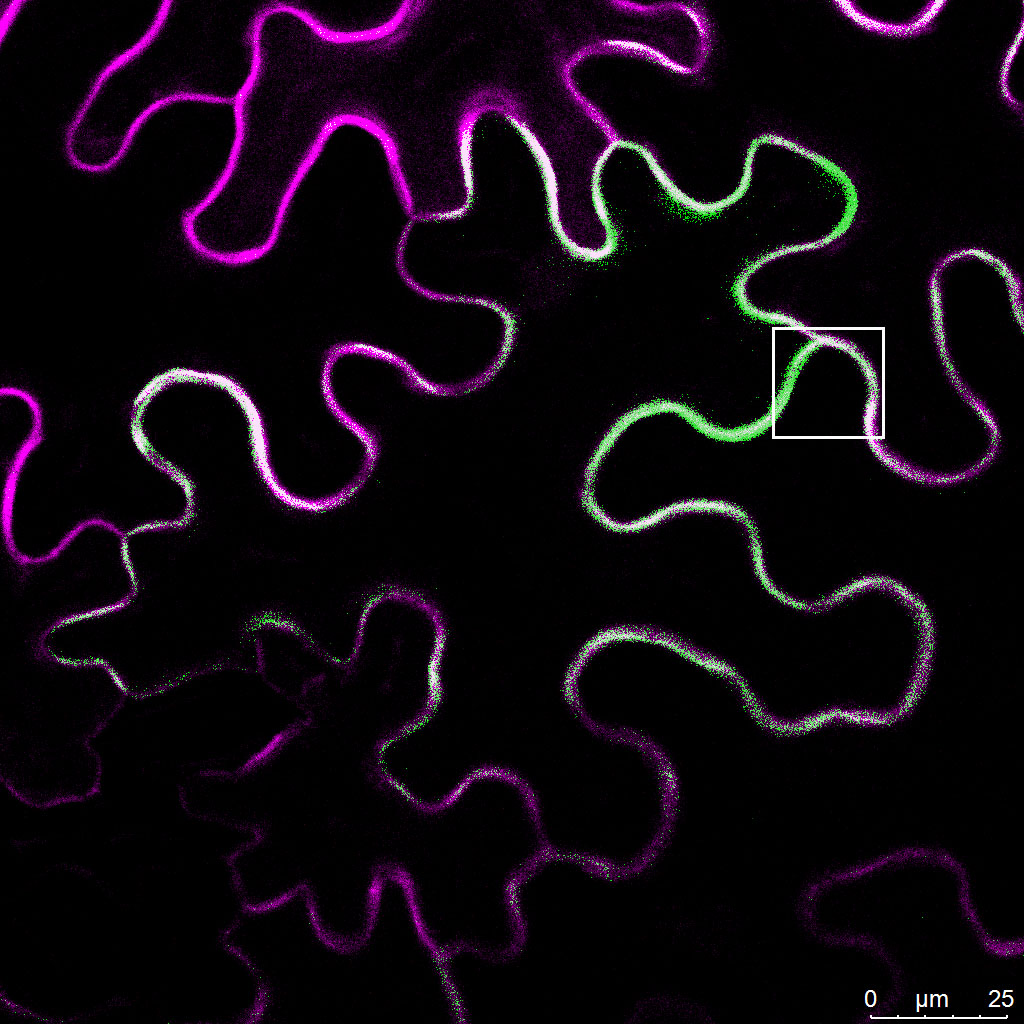

Supplement: Supplementary file 6 — Source data Fig. 4 [file 44318_2024_357_MOESM6_ESM.zip › Figure 4/Fig.4D/NaCl/Merge---GmNPF7.5 GFP-AtCBL1n mCherry.jpg]

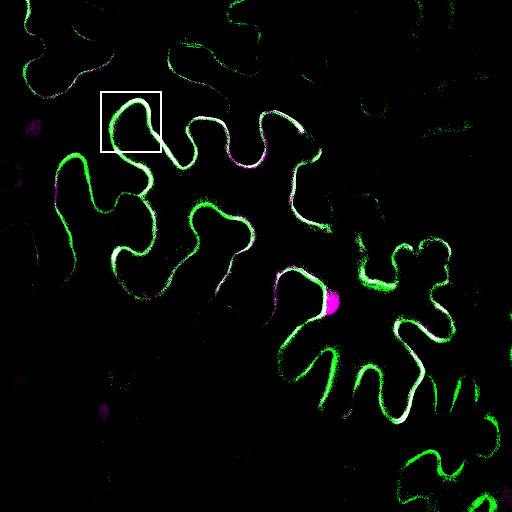

Supplement: Supplementary file 6 — Source data Fig. 4 [file 44318_2024_357_MOESM6_ESM.zip › Figure 4/Fig.4D/NaCl/Merge---GmNPF7.5 GFP-GmPI4Kγ4 mCherry.jpg]

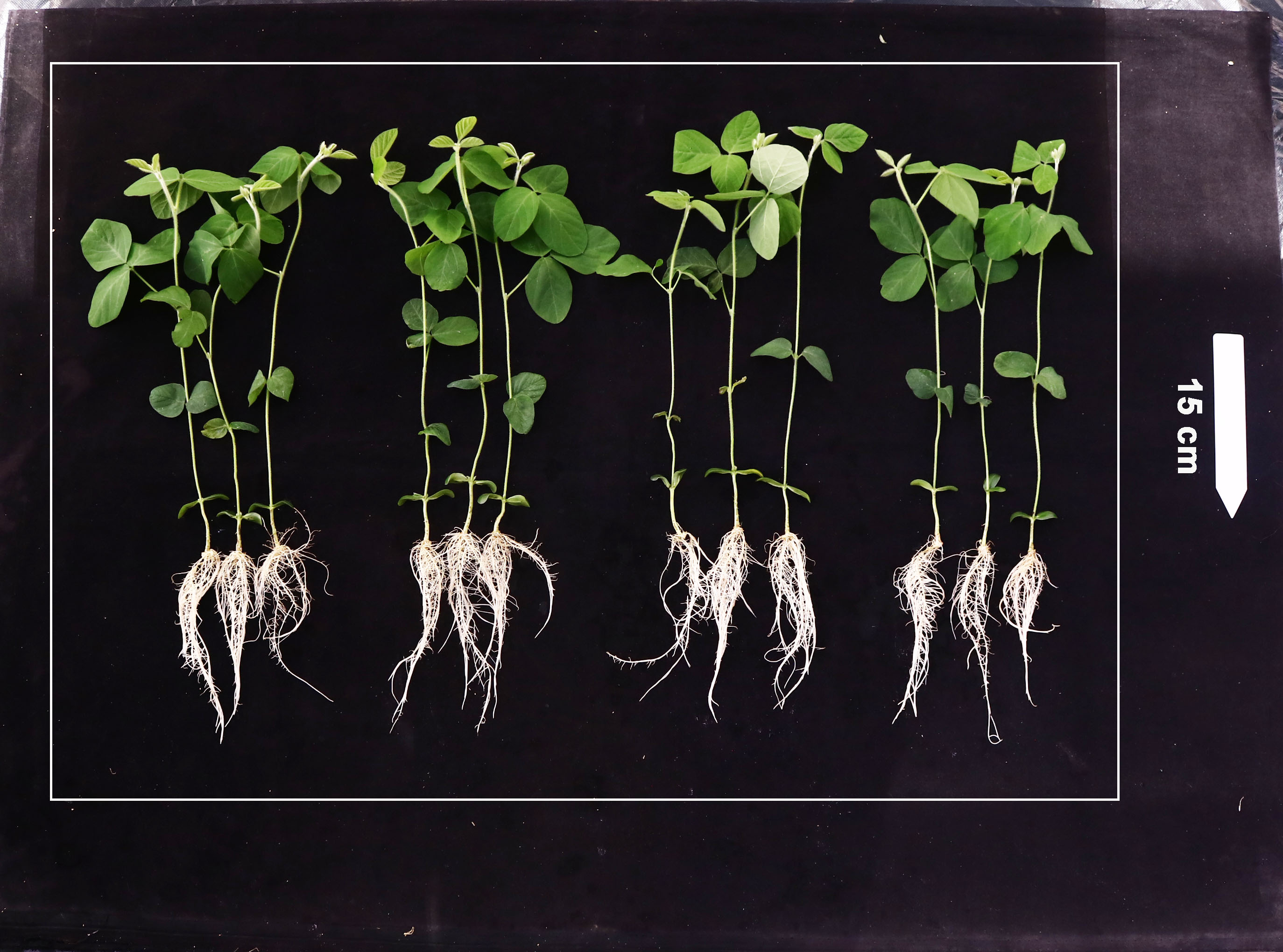

Supplement: Supplementary file 6 — Source data Fig. 4 [file 44318_2024_357_MOESM6_ESM.zip › Figure 4/Fig.4F/Control.jpg]

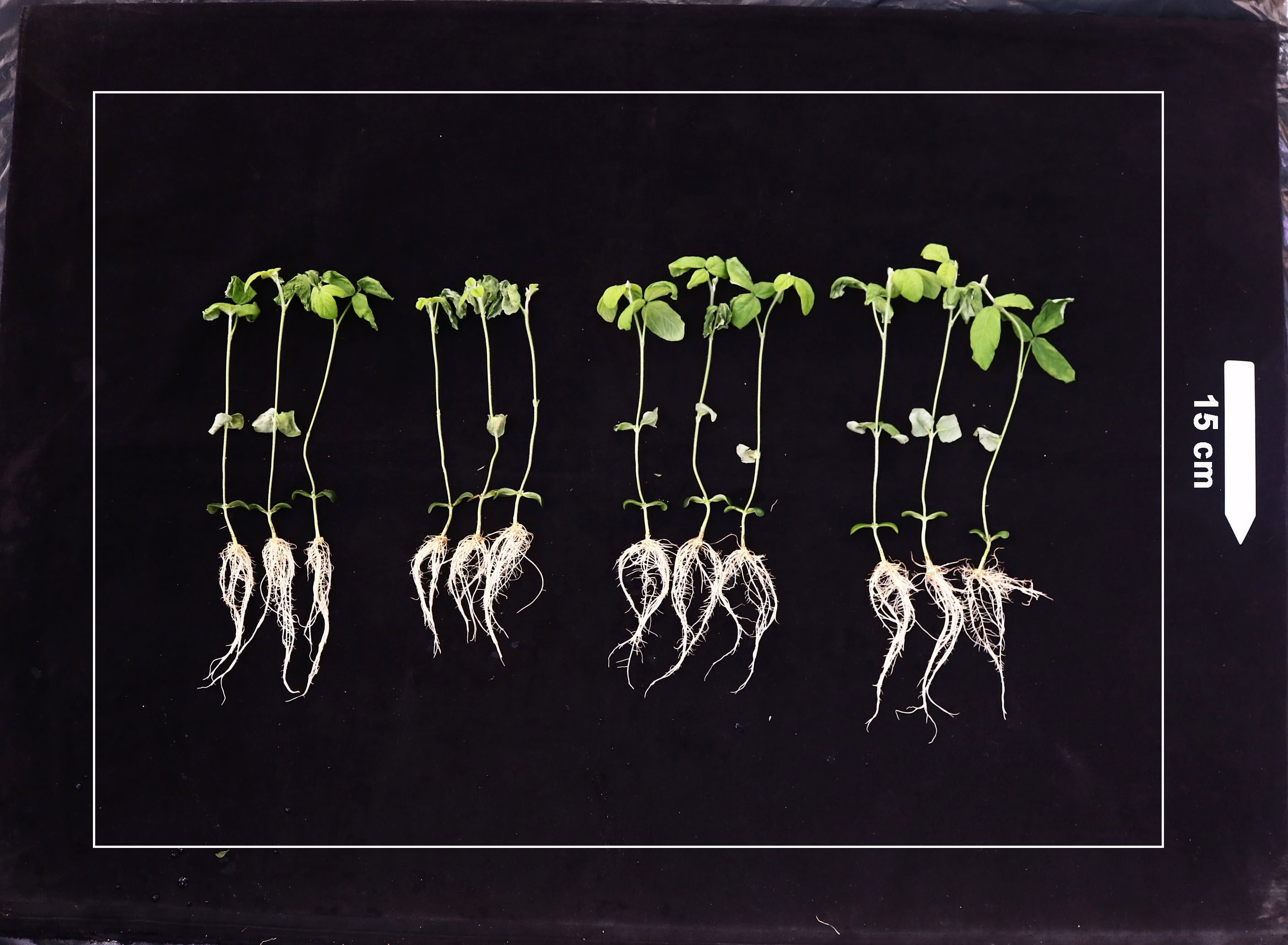

Supplement: Supplementary file 6 — Source data Fig. 4 [file 44318_2024_357_MOESM6_ESM.zip › Figure 4/Fig.4F/NaCl.jpg]

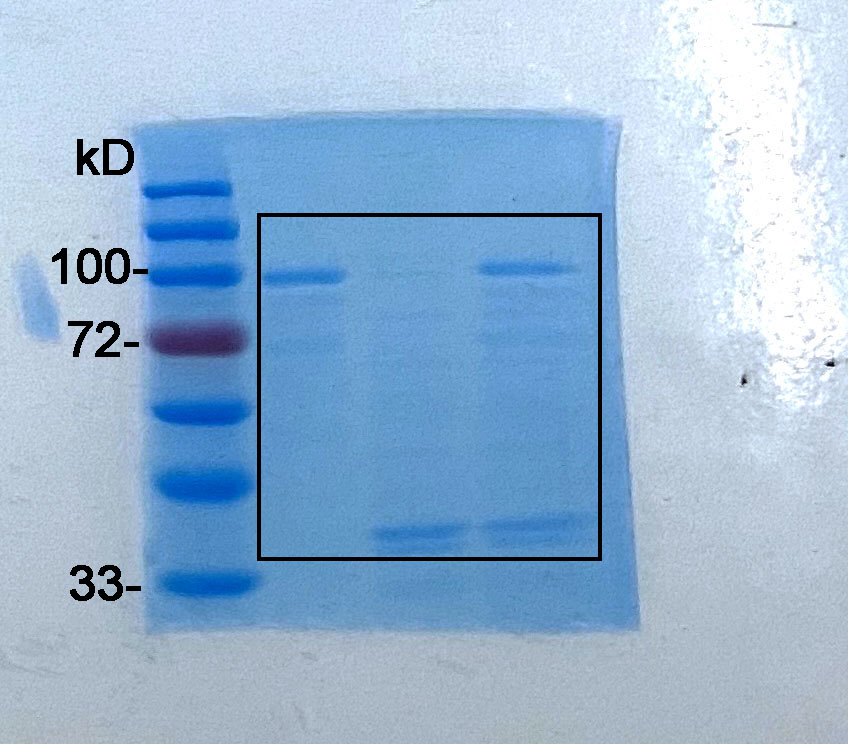

Supplement: Supplementary file 7 — Source data Fig. 5 [file 44318_2024_357_MOESM7_ESM.zip › Figure 5/Fig.5A/A-CBB.jpg]

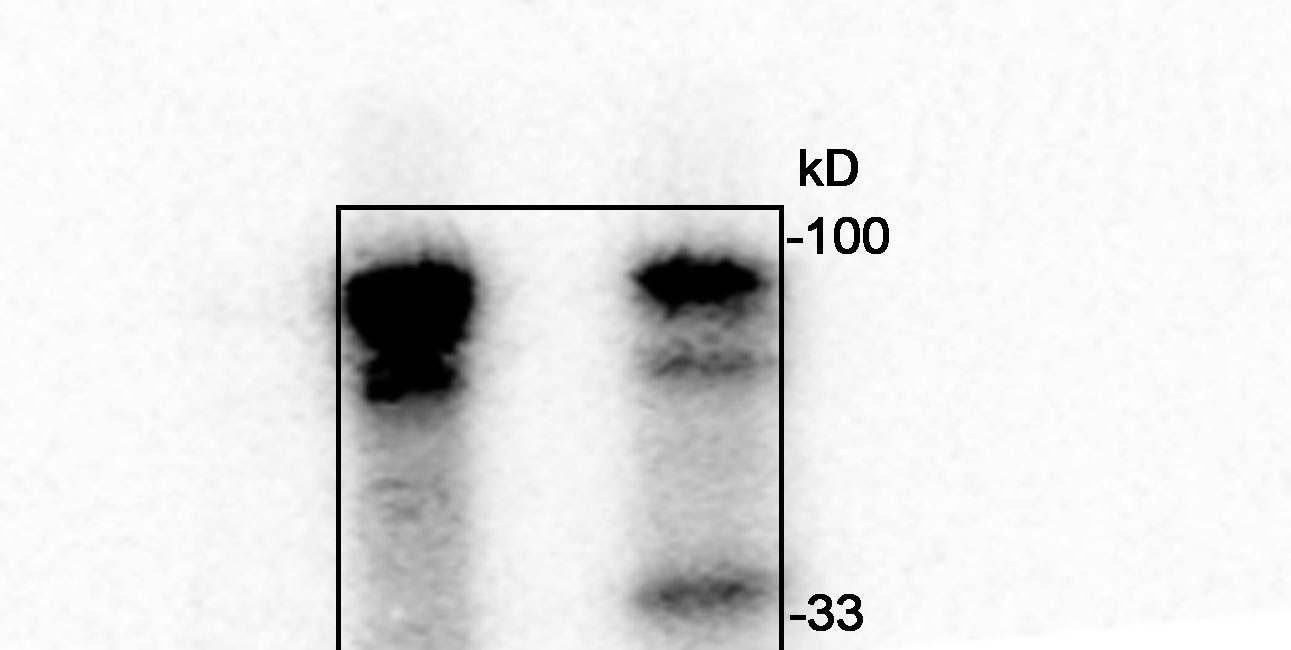

Supplement: Supplementary file 7 — Source data Fig. 5 [file 44318_2024_357_MOESM7_ESM.zip › Figure 5/Fig.5A/A-Phosphorylation.jpg]

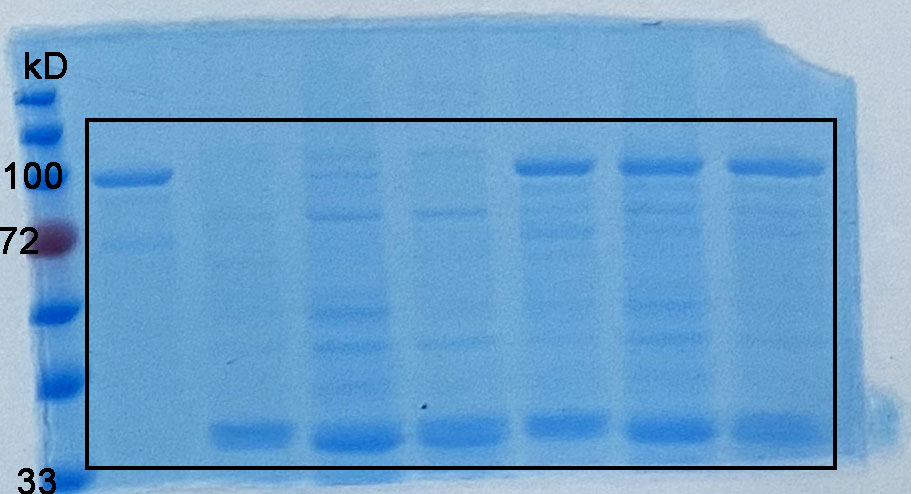

Supplement: Supplementary file 7 — Source data Fig. 5 [file 44318_2024_357_MOESM7_ESM.zip › Figure 5/Fig.5B/B-CBB.jpg]

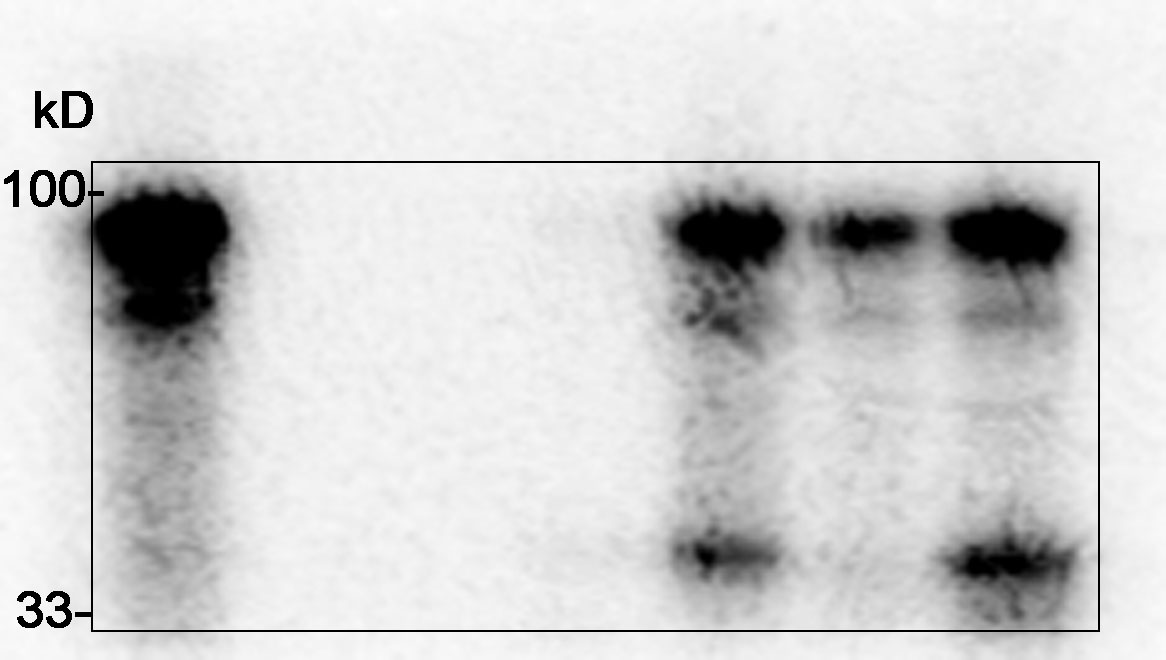

Supplement: Supplementary file 7 — Source data Fig. 5 [file 44318_2024_357_MOESM7_ESM.zip › Figure 5/Fig.5B/B-Phosphorylation.jpg]

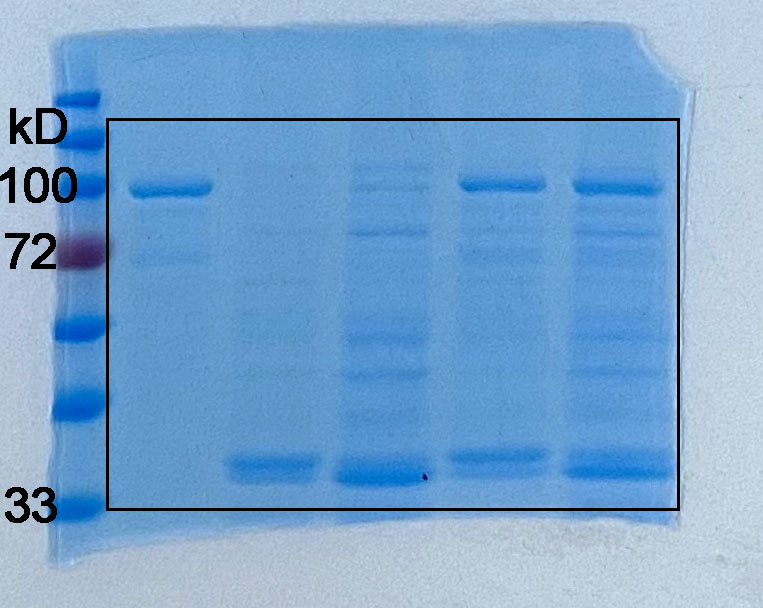

Supplement: Supplementary file 7 — Source data Fig. 5 [file 44318_2024_357_MOESM7_ESM.zip › Figure 5/Fig.5D/D-CBB.jpg]

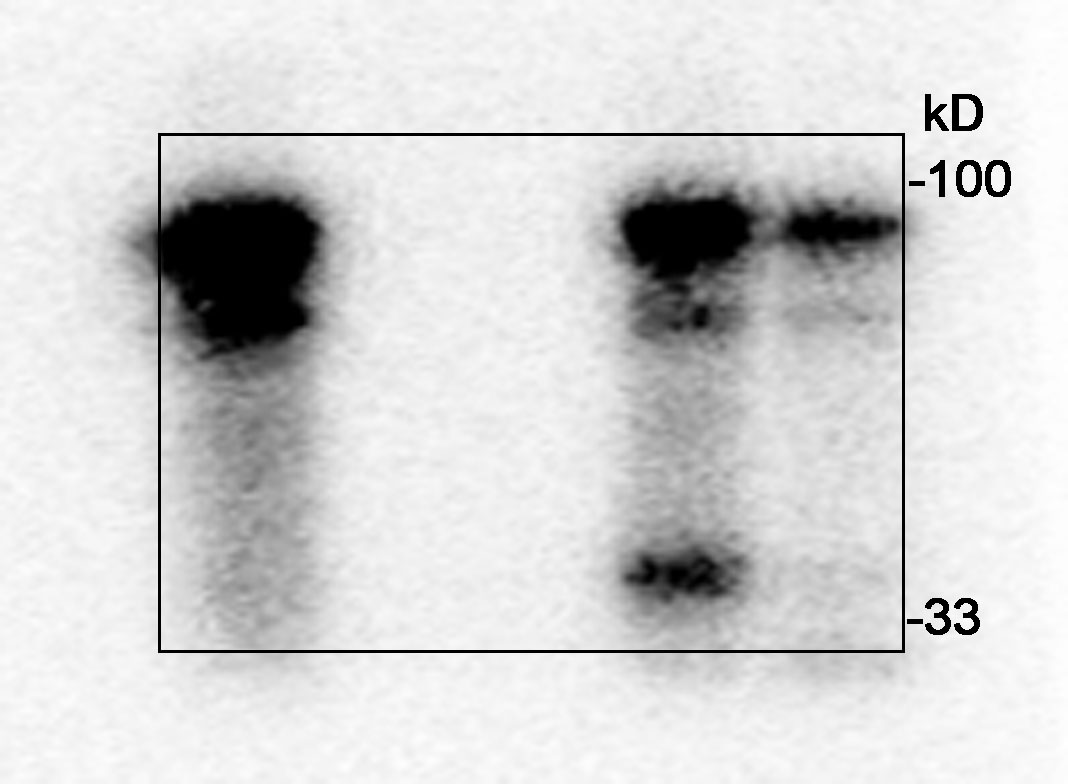

Supplement: Supplementary file 7 — Source data Fig. 5 [file 44318_2024_357_MOESM7_ESM.zip › Figure 5/Fig.5D/D-Phosphorylation.jpg]

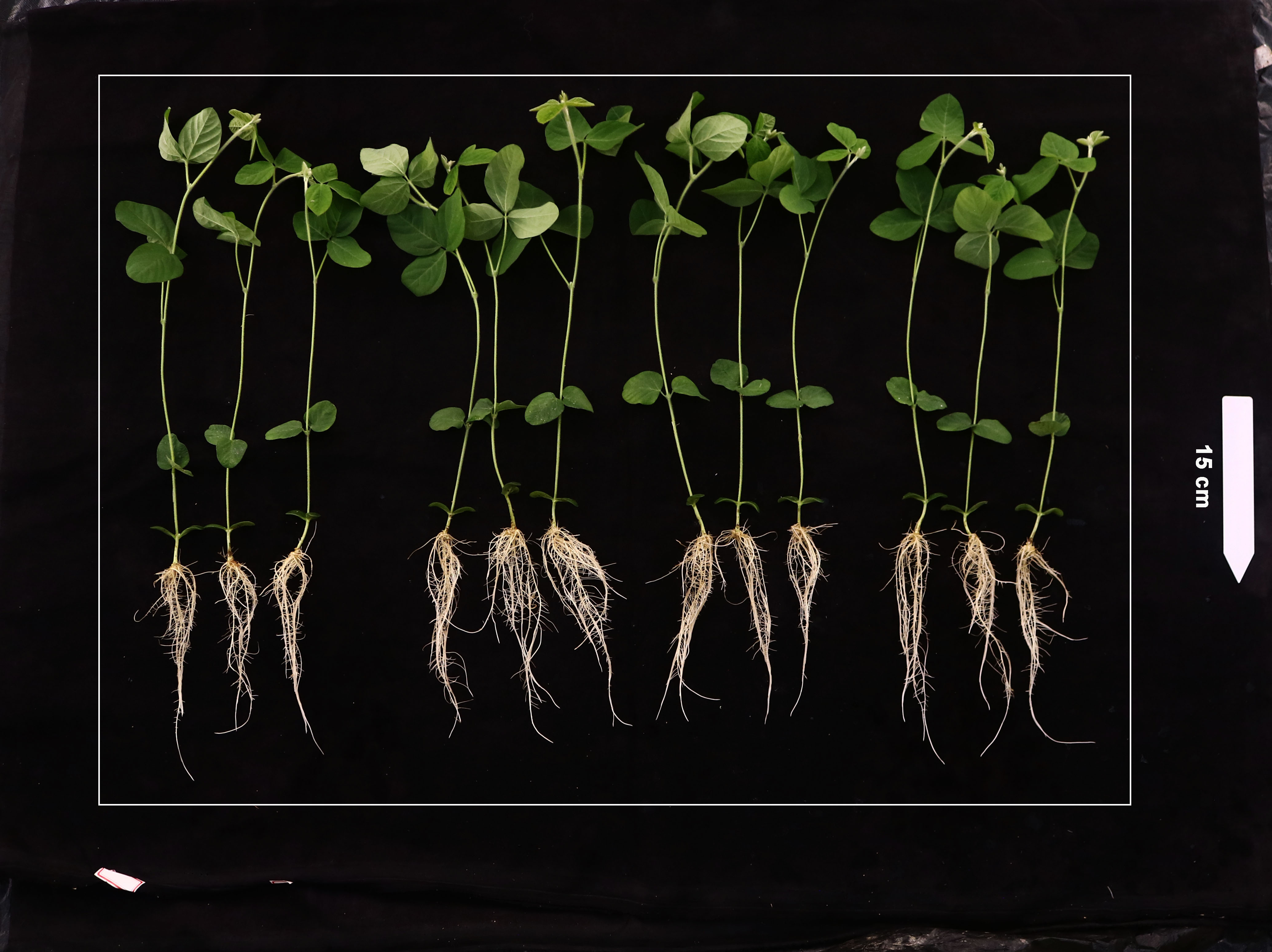

Supplement: Supplementary file 7 — Source data Fig. 5 [file 44318_2024_357_MOESM7_ESM.zip › Figure 5/Fig.5G/Control.jpg]

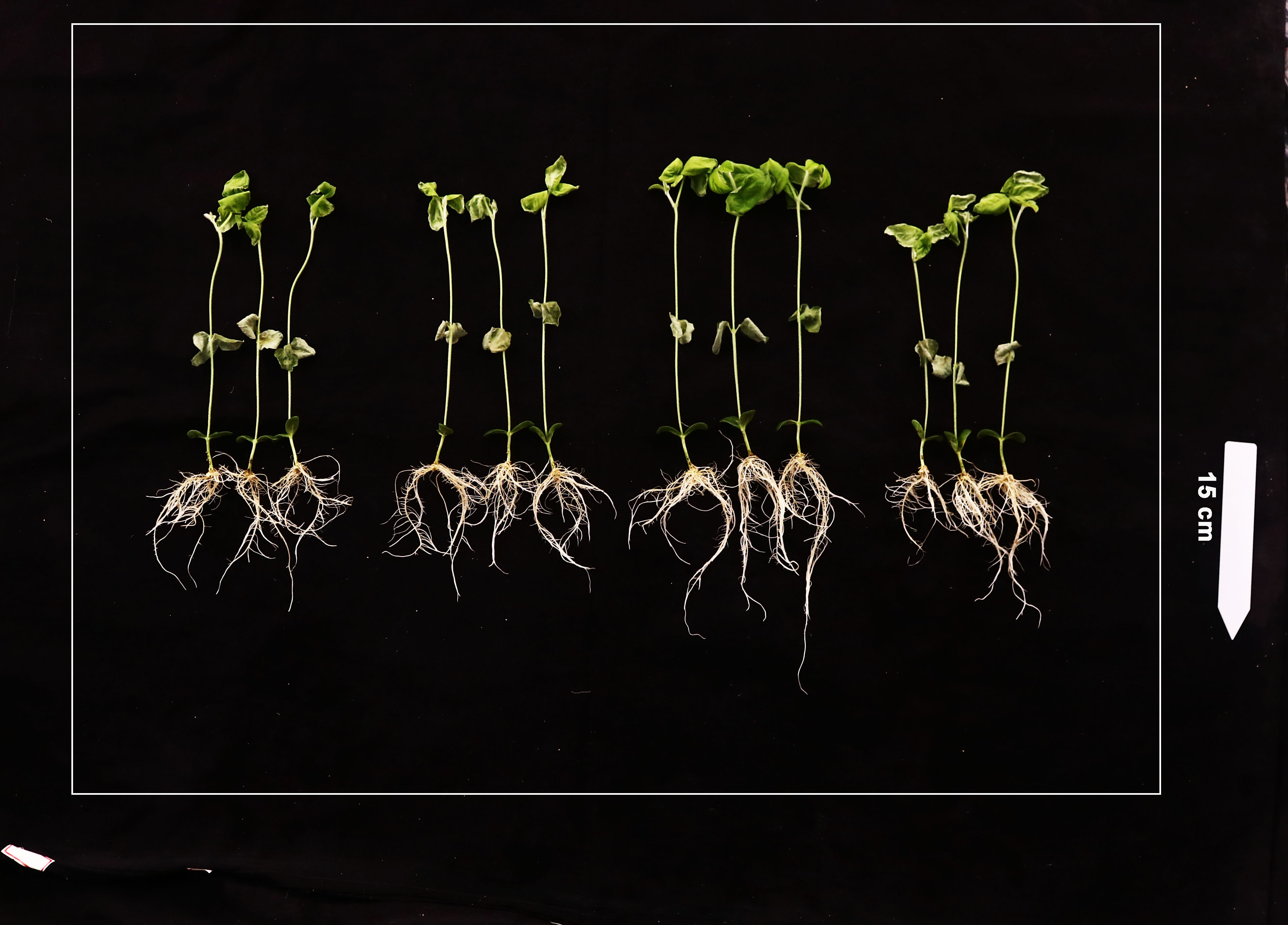

Supplement: Supplementary file 7 — Source data Fig. 5 [file 44318_2024_357_MOESM7_ESM.zip › Figure 5/Fig.5G/NaCl.jpg]

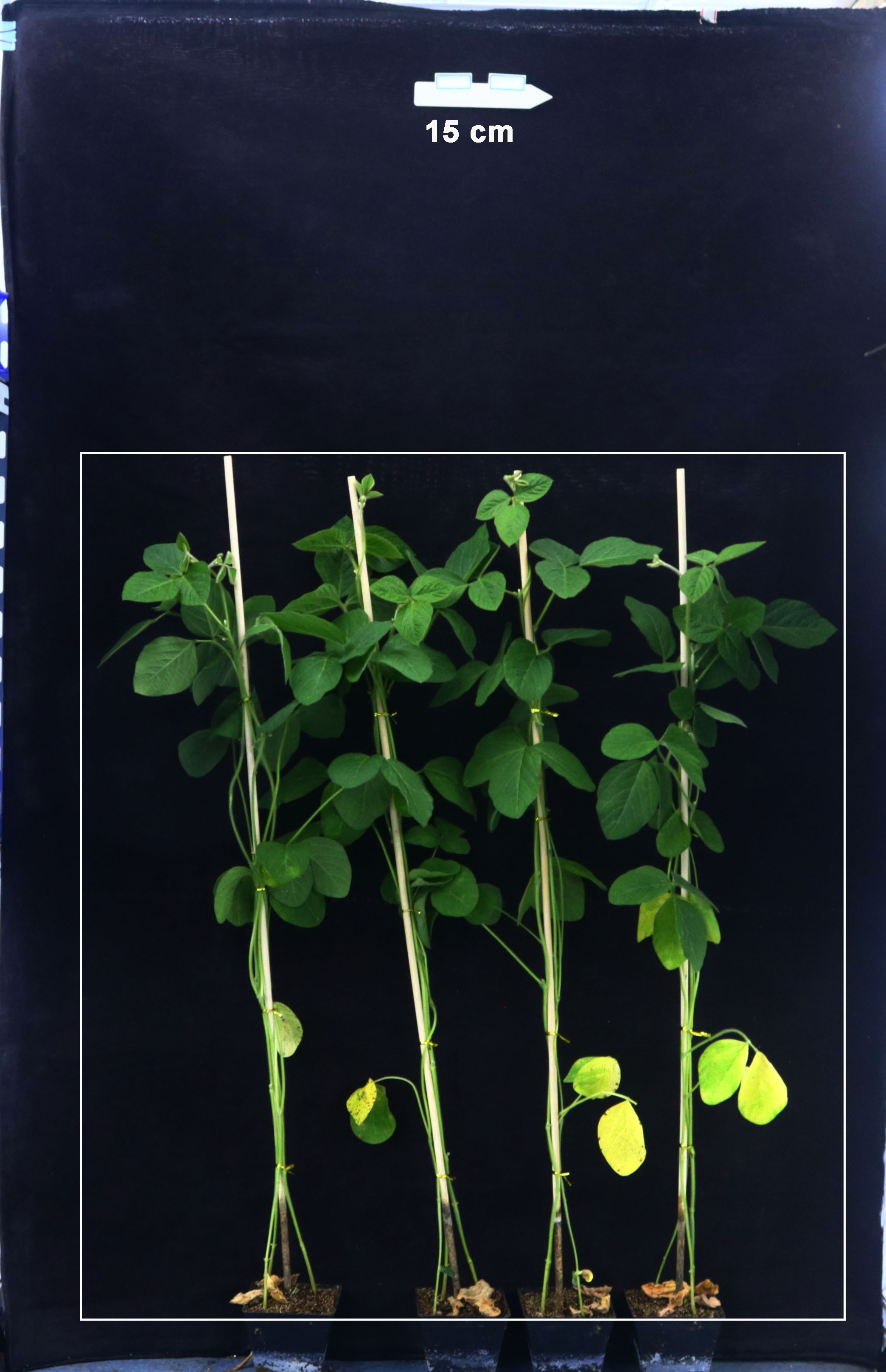

Supplement: Supplementary file 8 — Source data Fig. 6 [file 44318_2024_357_MOESM8_ESM.zip › Figure 6/Fig.6A/Control.jpg]

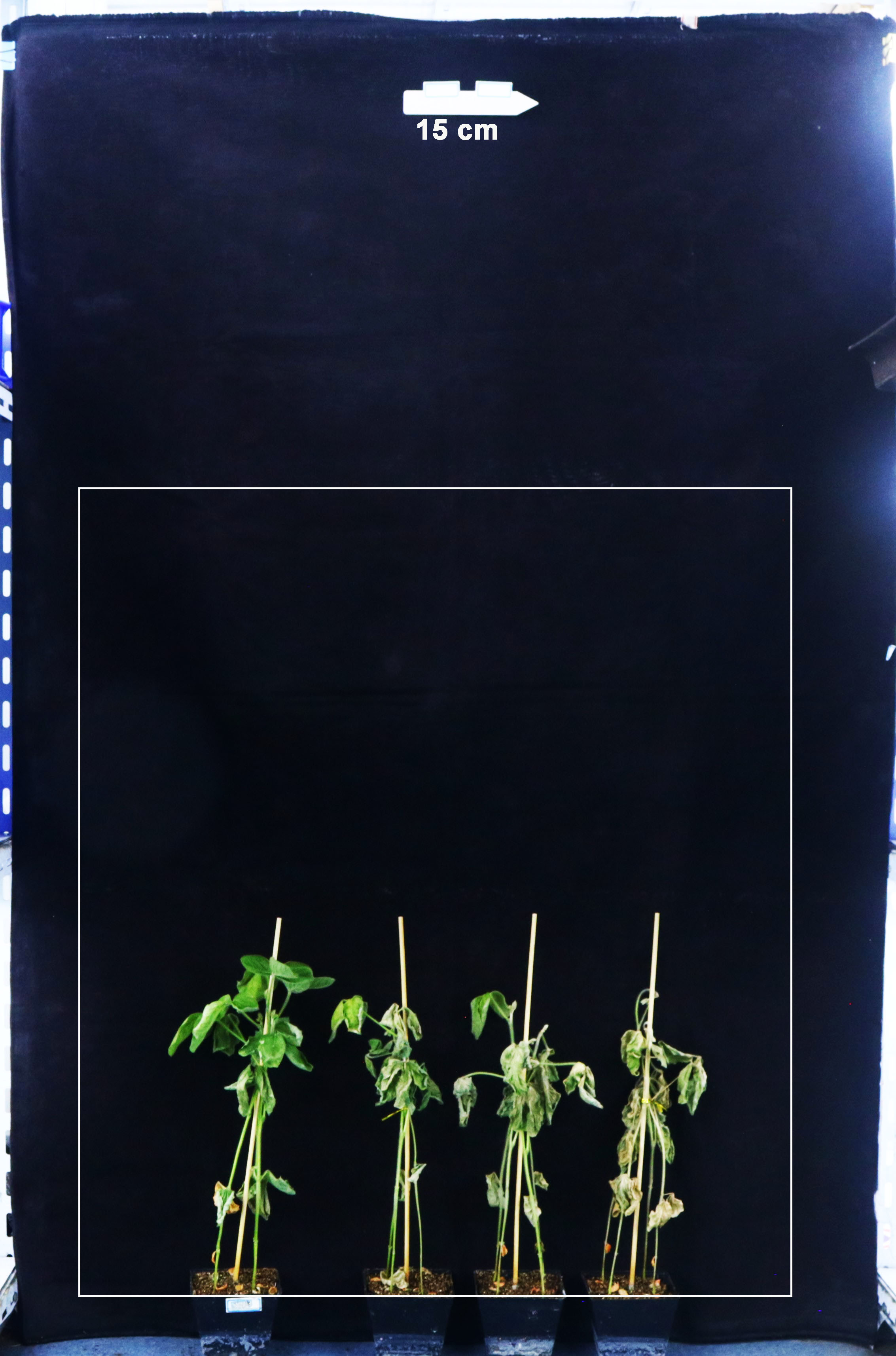

Supplement: Supplementary file 8 — Source data Fig. 6 [file 44318_2024_357_MOESM8_ESM.zip › Figure 6/Fig.6A/NaCl.jpg]

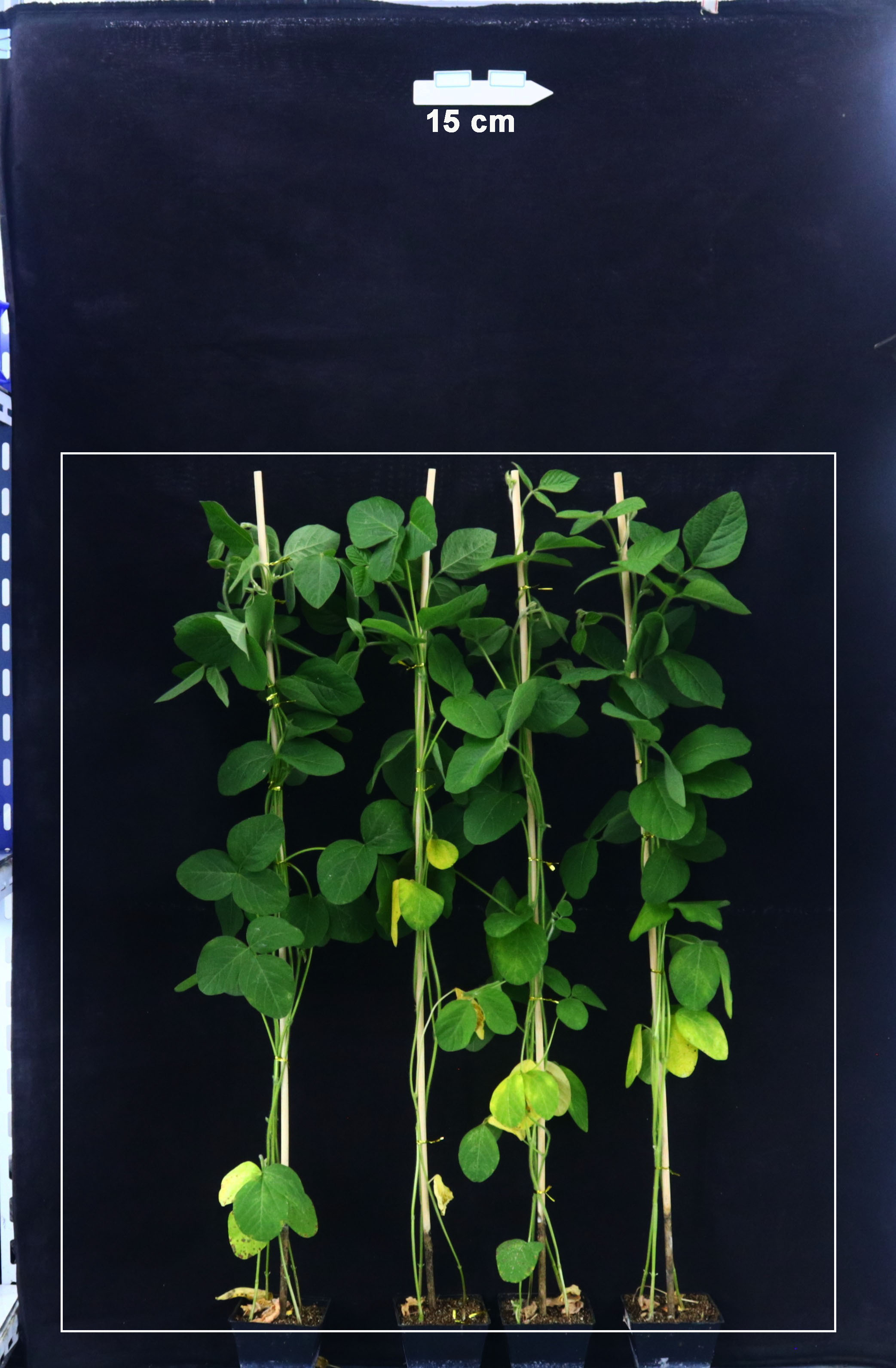

Supplement: Supplementary file 8 — Source data Fig. 6 [file 44318_2024_357_MOESM8_ESM.zip › Figure 6/Fig.6C/Control.jpg]

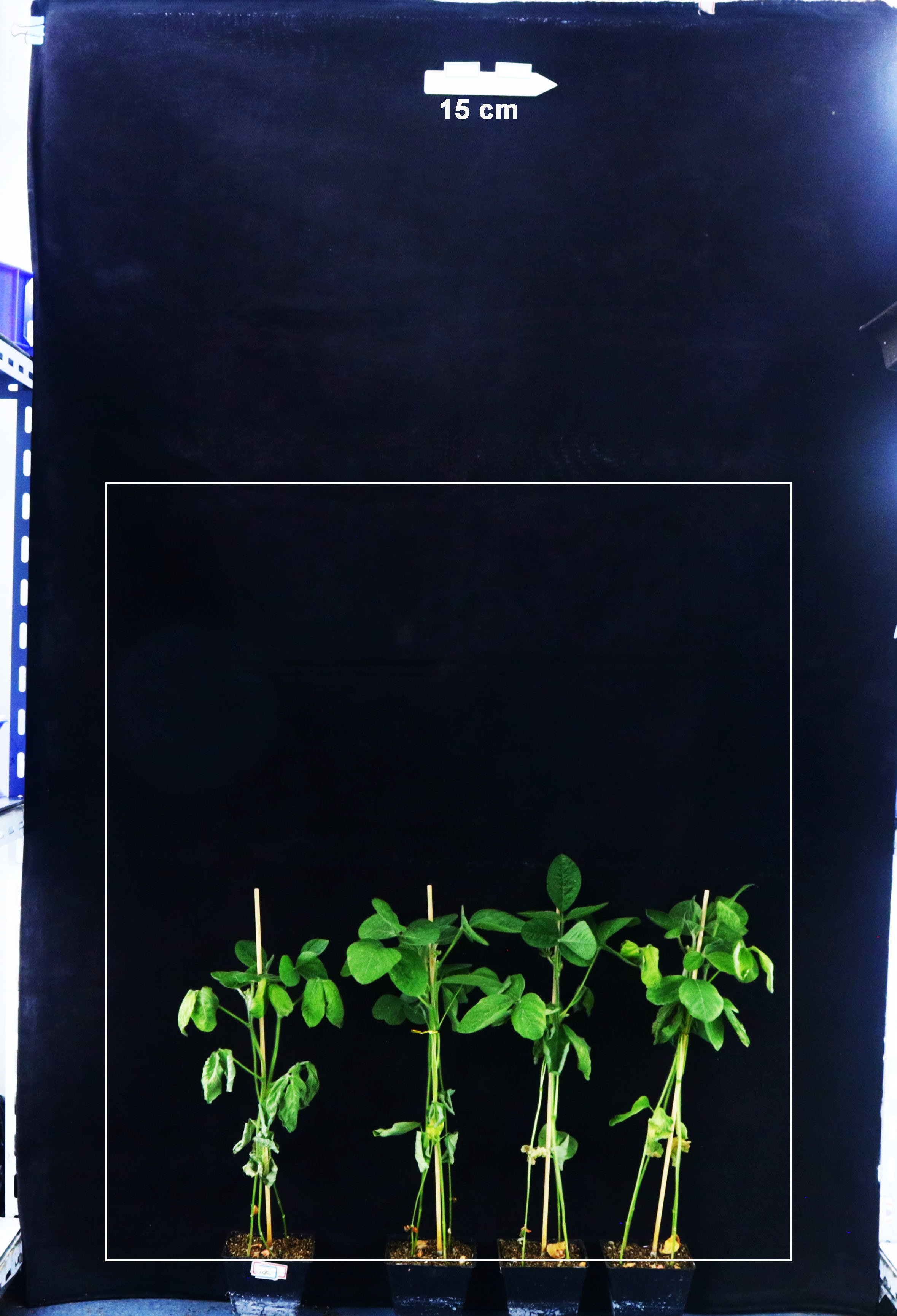

Supplement: Supplementary file 8 — Source data Fig. 6 [file 44318_2024_357_MOESM8_ESM.zip › Figure 6/Fig.6C/NaCl.jpg]

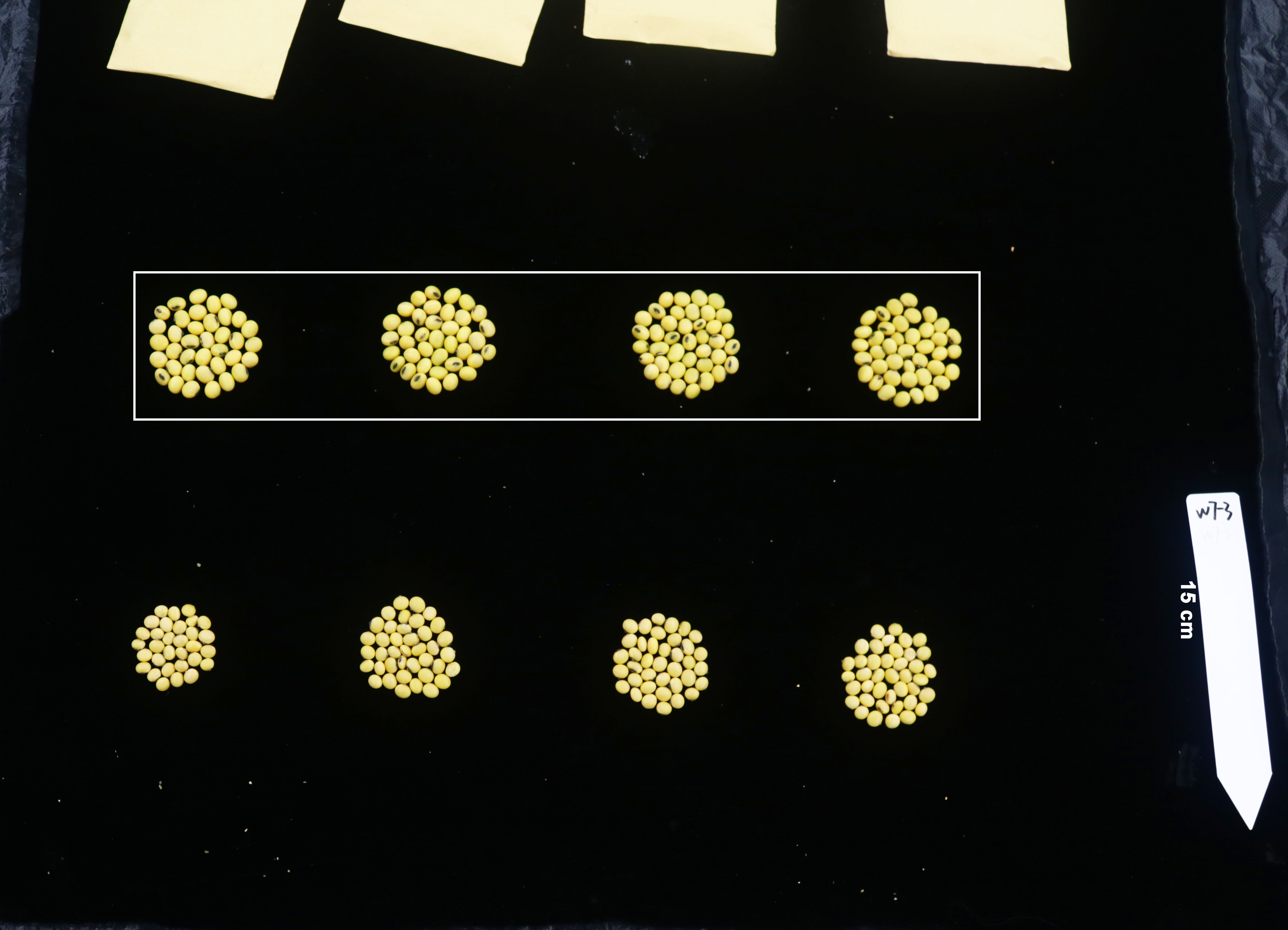

Supplement: Supplementary file 8 — Source data Fig. 6 [file 44318_2024_357_MOESM8_ESM.zip › Figure 6/Fig.6G/Wide type vs GmNPF7.5-crispr/Normai condition.jpg]

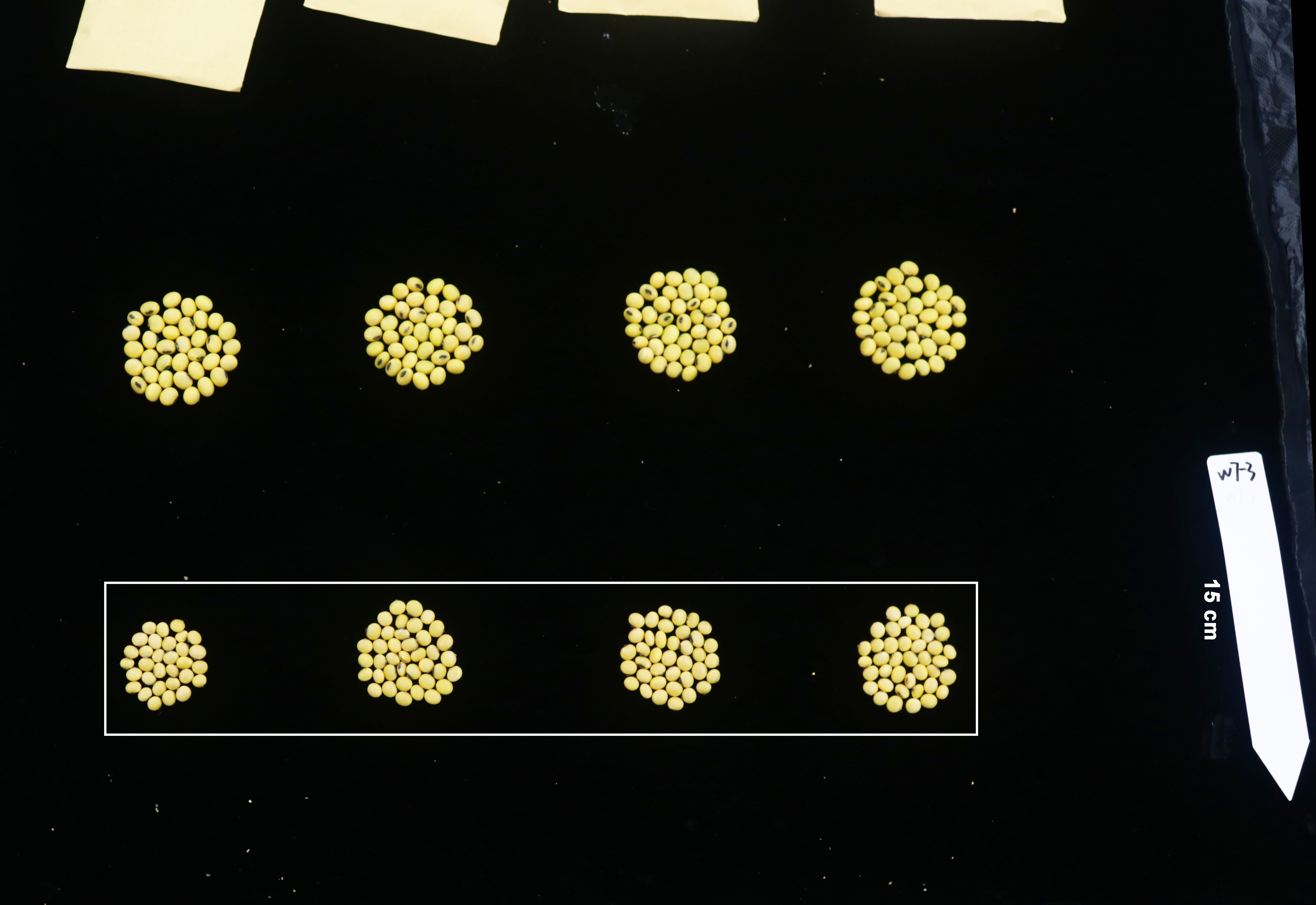

Supplement: Supplementary file 8 — Source data Fig. 6 [file 44318_2024_357_MOESM8_ESM.zip › Figure 6/Fig.6G/Wide type vs GmNPF7.5-crispr/Salinity.jpg]

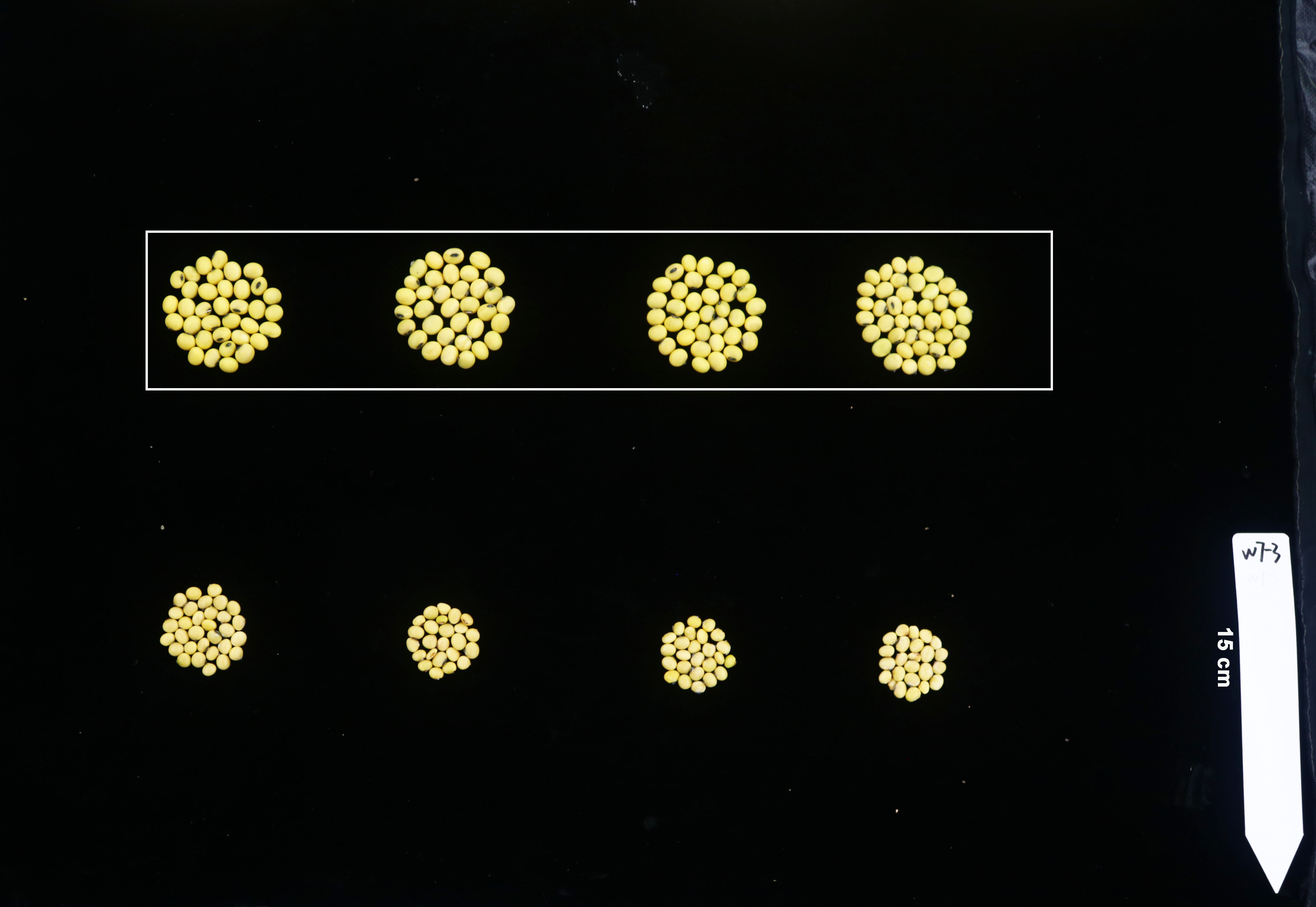

Supplement: Supplementary file 8 — Source data Fig. 6 [file 44318_2024_357_MOESM8_ESM.zip › Figure 6/Fig.6G/Wide type vs GmNPF7.5-OX/Normal condition.jpg]

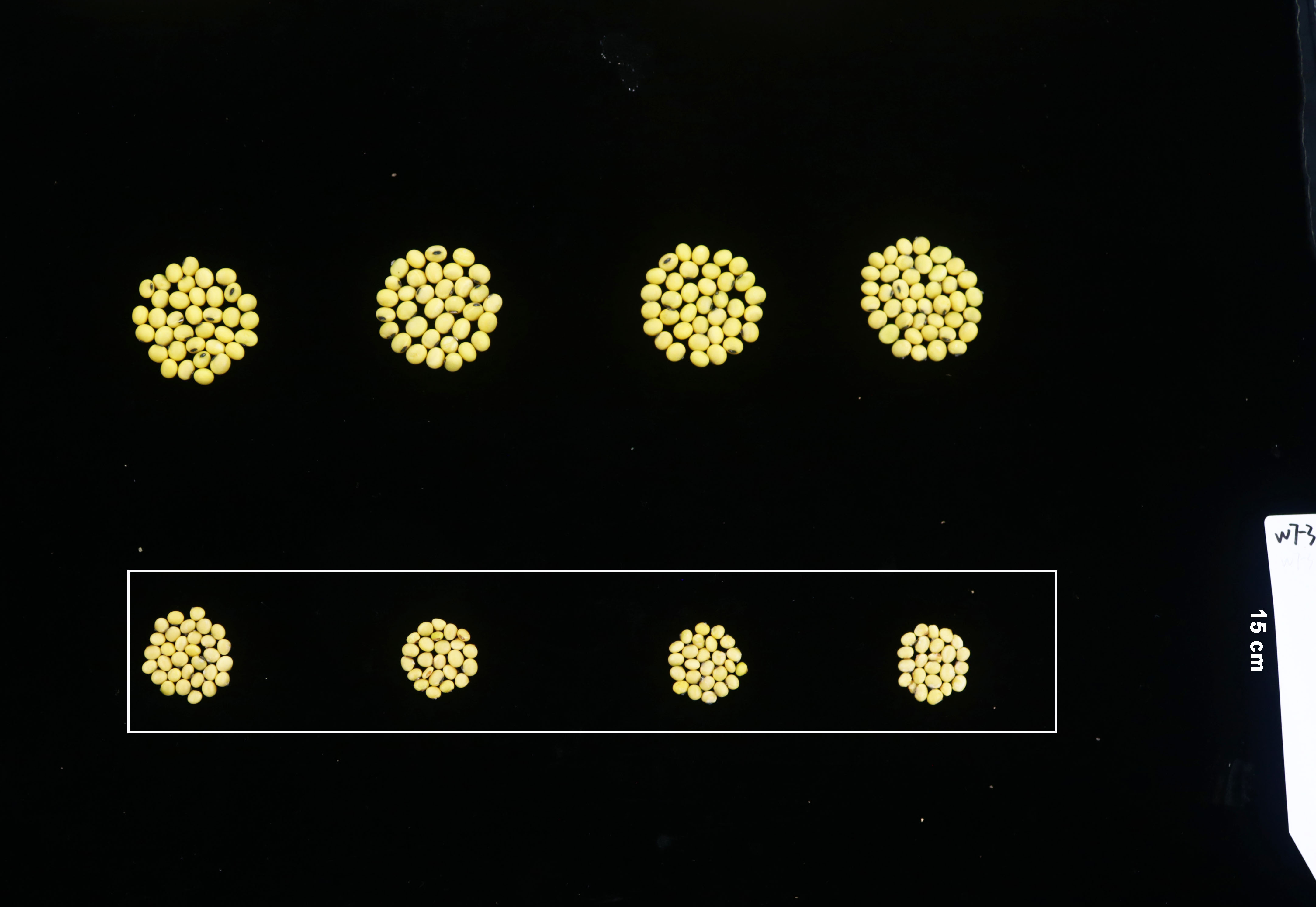

Supplement: Supplementary file 8 — Source data Fig. 6 [file 44318_2024_357_MOESM8_ESM.zip › Figure 6/Fig.6G/Wide type vs GmNPF7.5-OX/Salinity.jpg]

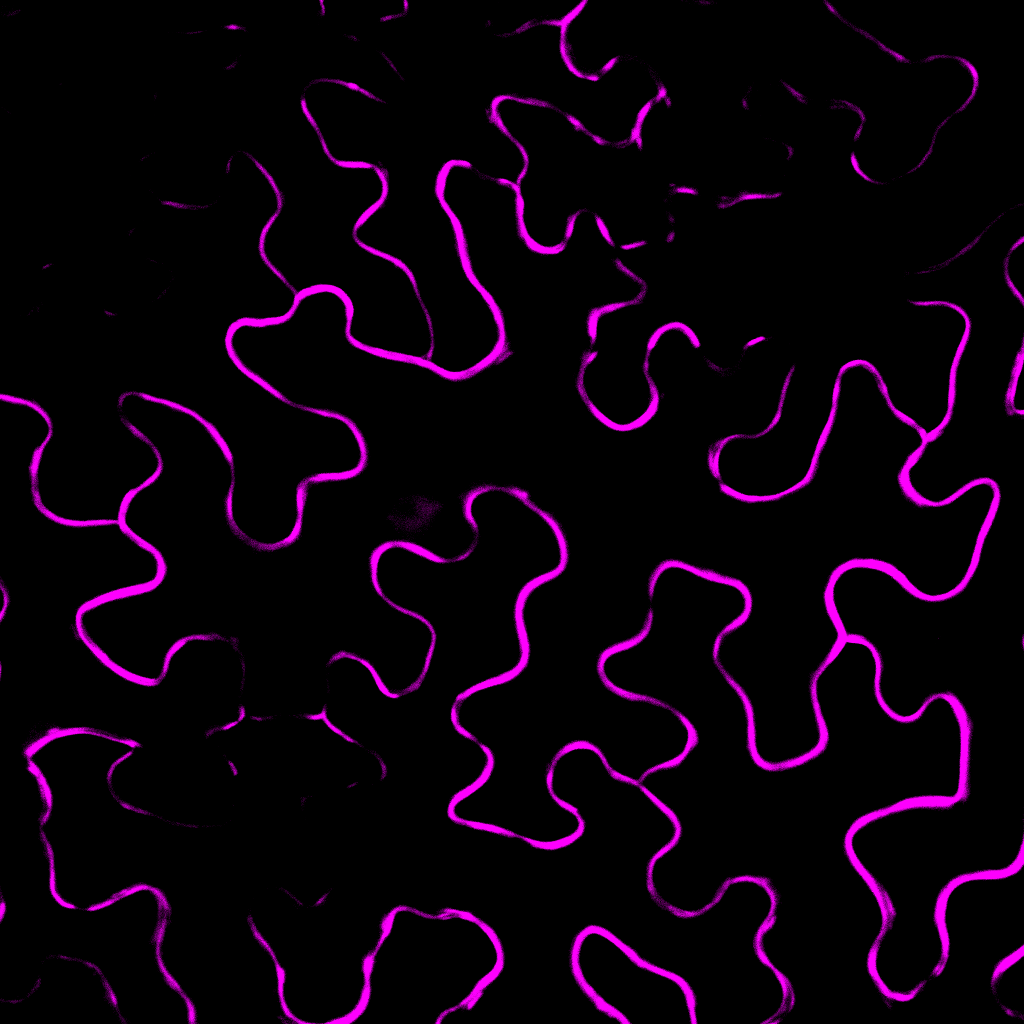

Supplement: Supplementary file 9 — EV and Appendix Figure Source Data [file 44318_2024_357_MOESM9_ESM.zip › FigEV-Appendix/Appendix Fig.S2/S2B/GFP/AtCBL1n-mCherry.tif]

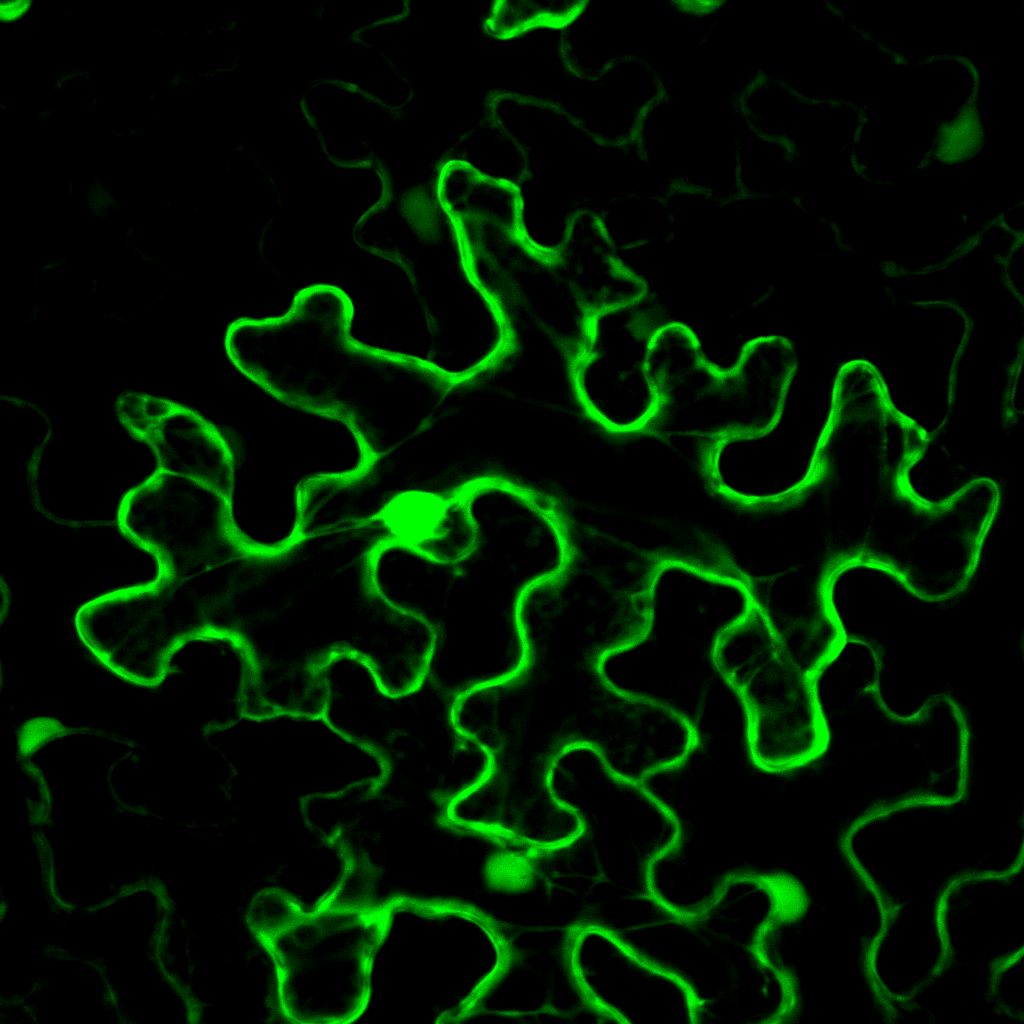

Supplement: Supplementary file 9 — EV and Appendix Figure Source Data [file 44318_2024_357_MOESM9_ESM.zip › FigEV-Appendix/Appendix Fig.S2/S2B/GFP/GFP.tif]

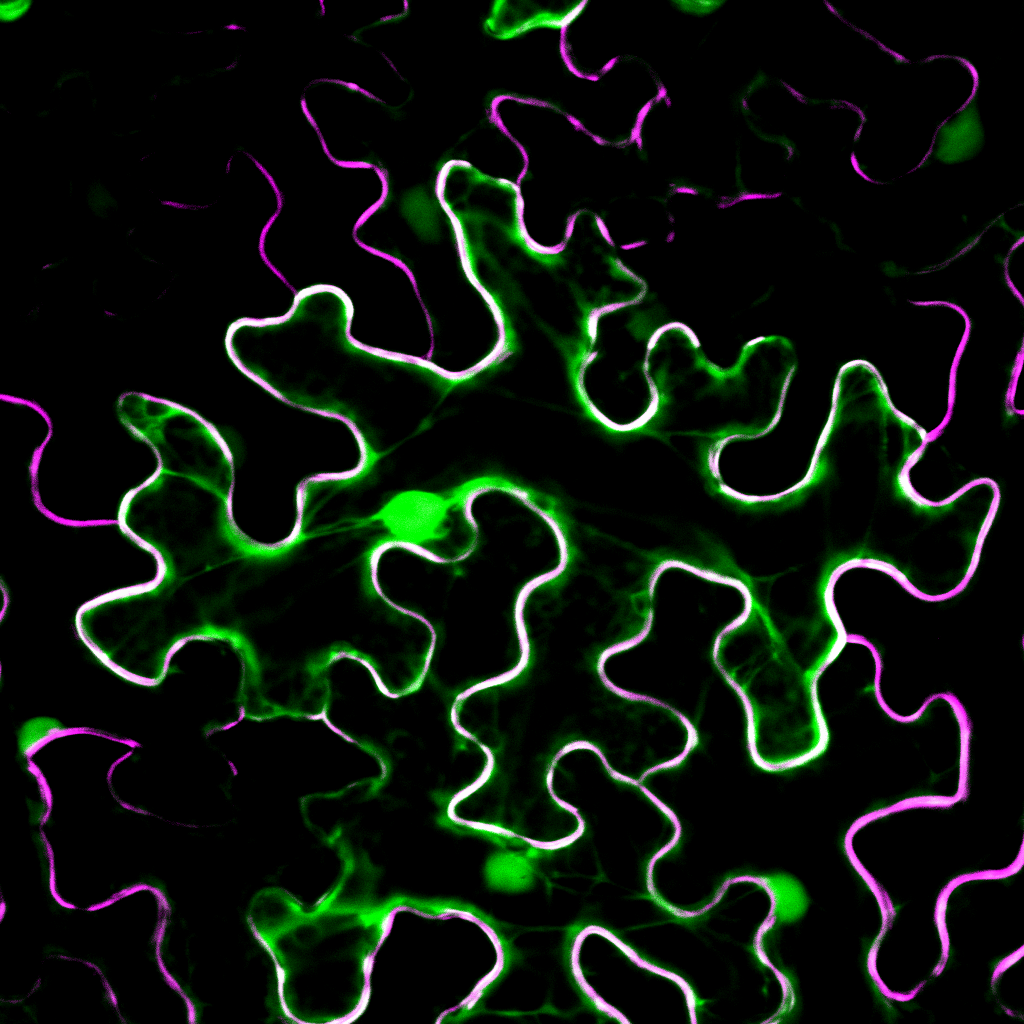

Supplement: Supplementary file 9 — EV and Appendix Figure Source Data [file 44318_2024_357_MOESM9_ESM.zip › FigEV-Appendix/Appendix Fig.S2/S2B/GFP/Merge.tif]

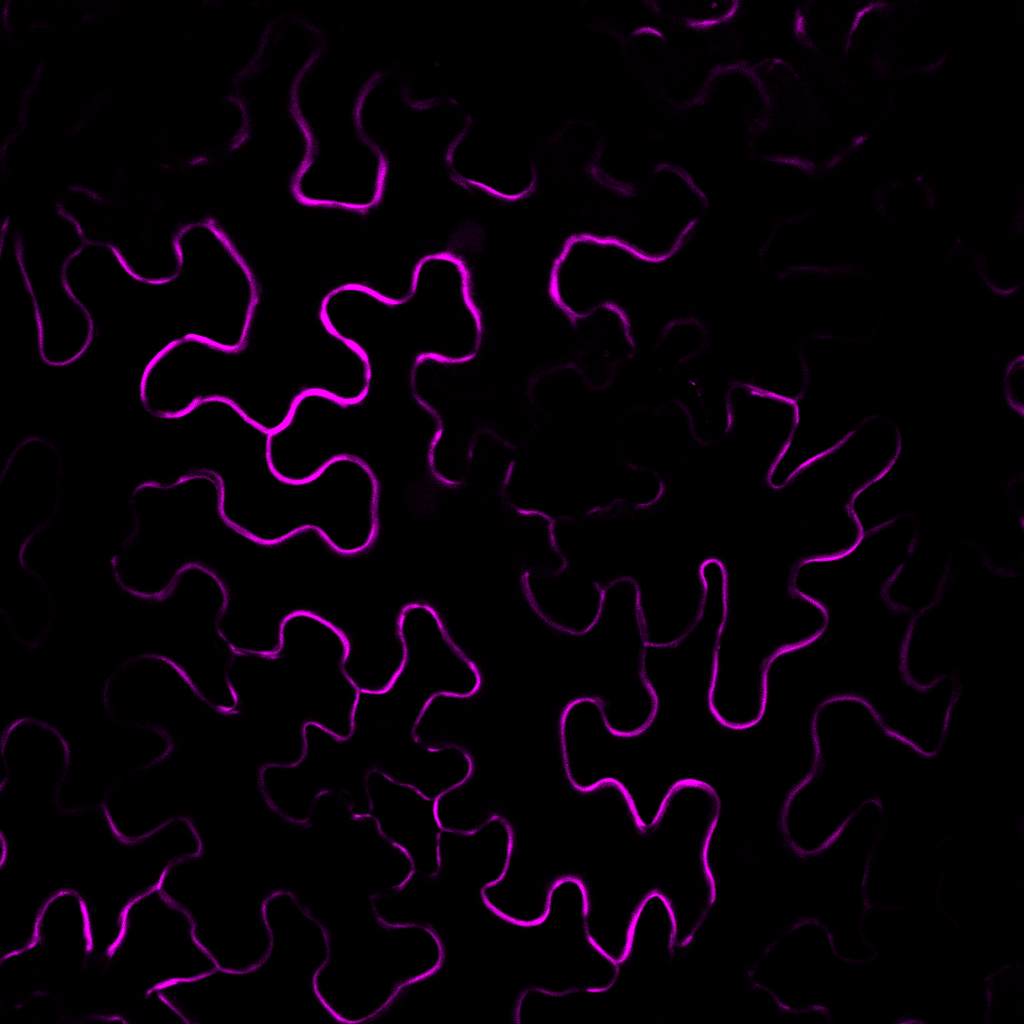

Supplement: Supplementary file 9 — EV and Appendix Figure Source Data [file 44318_2024_357_MOESM9_ESM.zip › FigEV-Appendix/Appendix Fig.S2/S2B/GmPI4Kγ4-GFP/AtCBL1n-mCherry.tif]

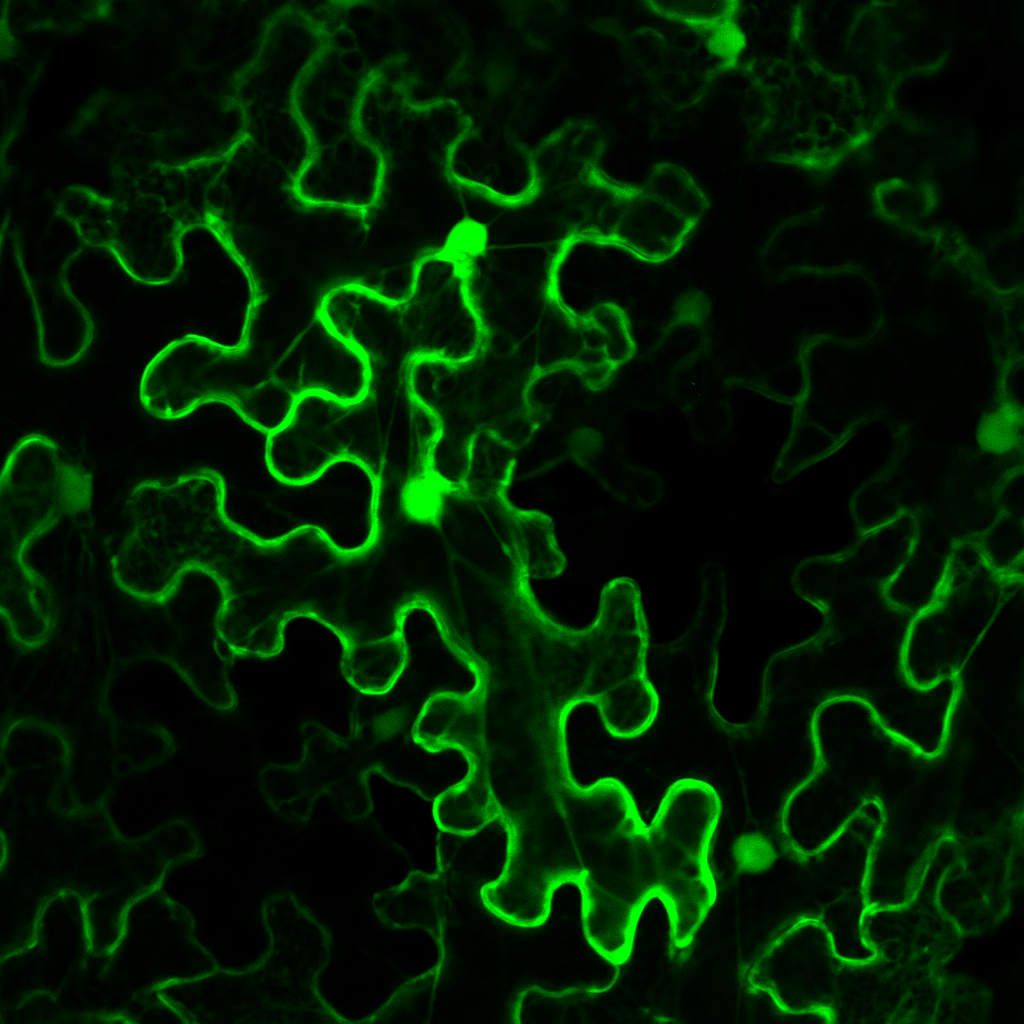

Supplement: Supplementary file 9 — EV and Appendix Figure Source Data [file 44318_2024_357_MOESM9_ESM.zip › FigEV-Appendix/Appendix Fig.S2/S2B/GmPI4Kγ4-GFP/GmPI4Kγ4-GFP.tif]

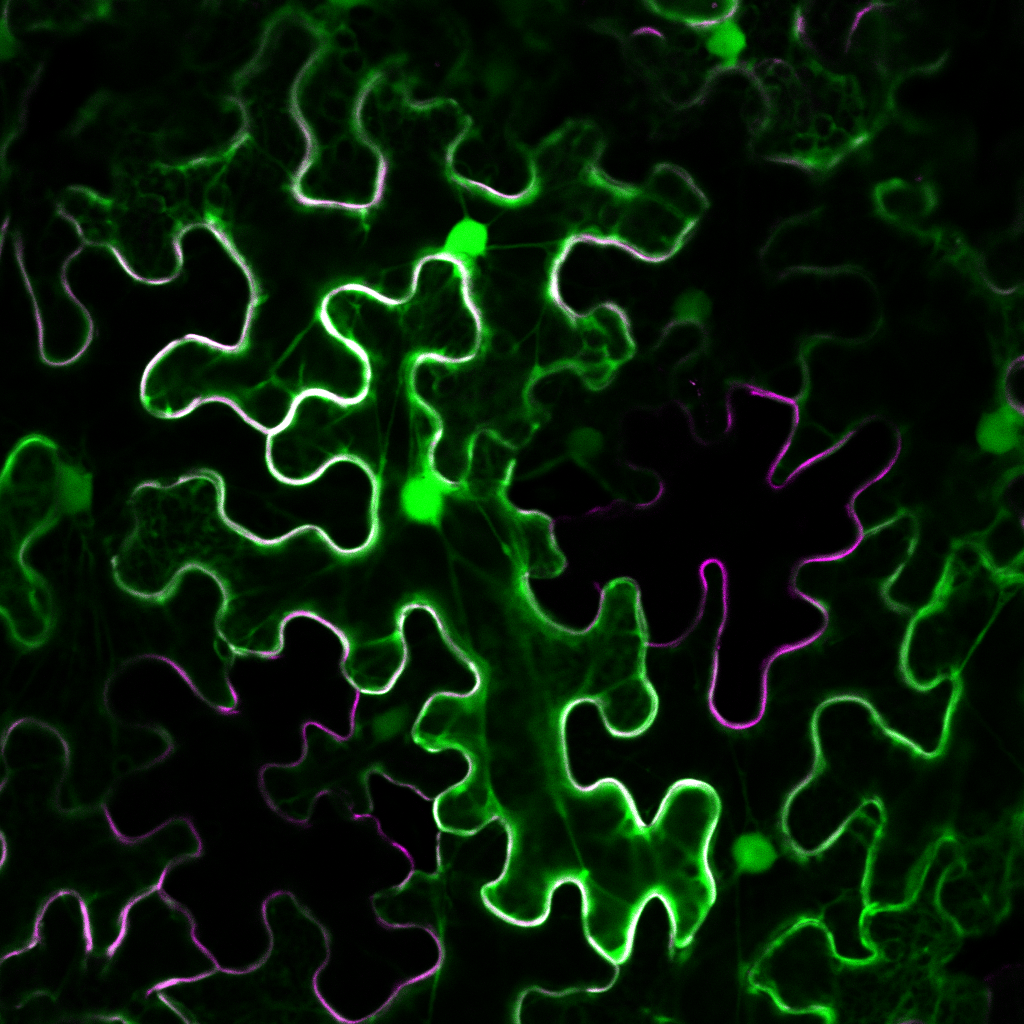

Supplement: Supplementary file 9 — EV and Appendix Figure Source Data [file 44318_2024_357_MOESM9_ESM.zip › FigEV-Appendix/Appendix Fig.S2/S2B/GmPI4Kγ4-GFP/Merge.tif]

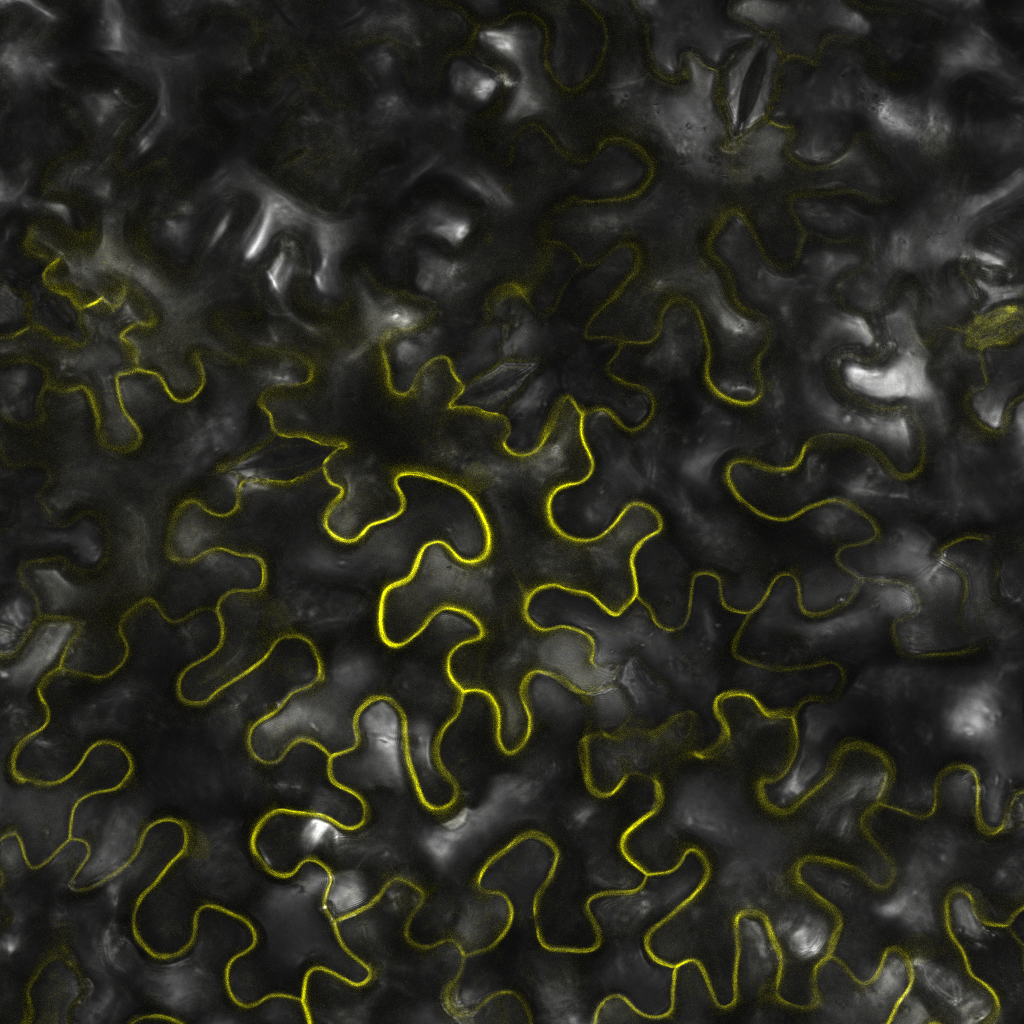

Supplement: Supplementary file 9 — EV and Appendix Figure Source Data [file 44318_2024_357_MOESM9_ESM.zip › FigEV-Appendix/Appendix Fig.S3/S3A/GmNPF7.5-YFPC+GmPI4Kγ4-YFPN/Overlay.tif]

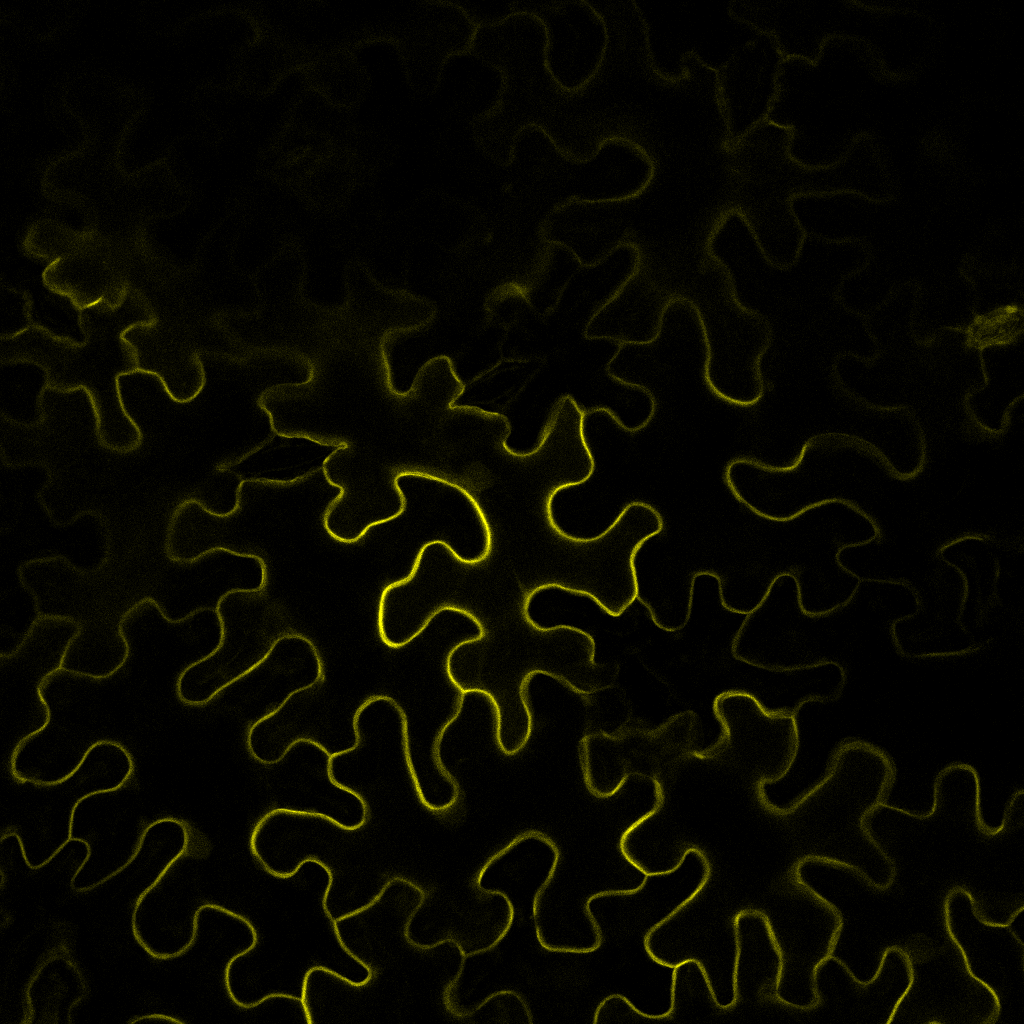

Supplement: Supplementary file 9 — EV and Appendix Figure Source Data [file 44318_2024_357_MOESM9_ESM.zip › FigEV-Appendix/Appendix Fig.S3/S3A/GmNPF7.5-YFPC+GmPI4Kγ4-YFPN/YFP.tif]

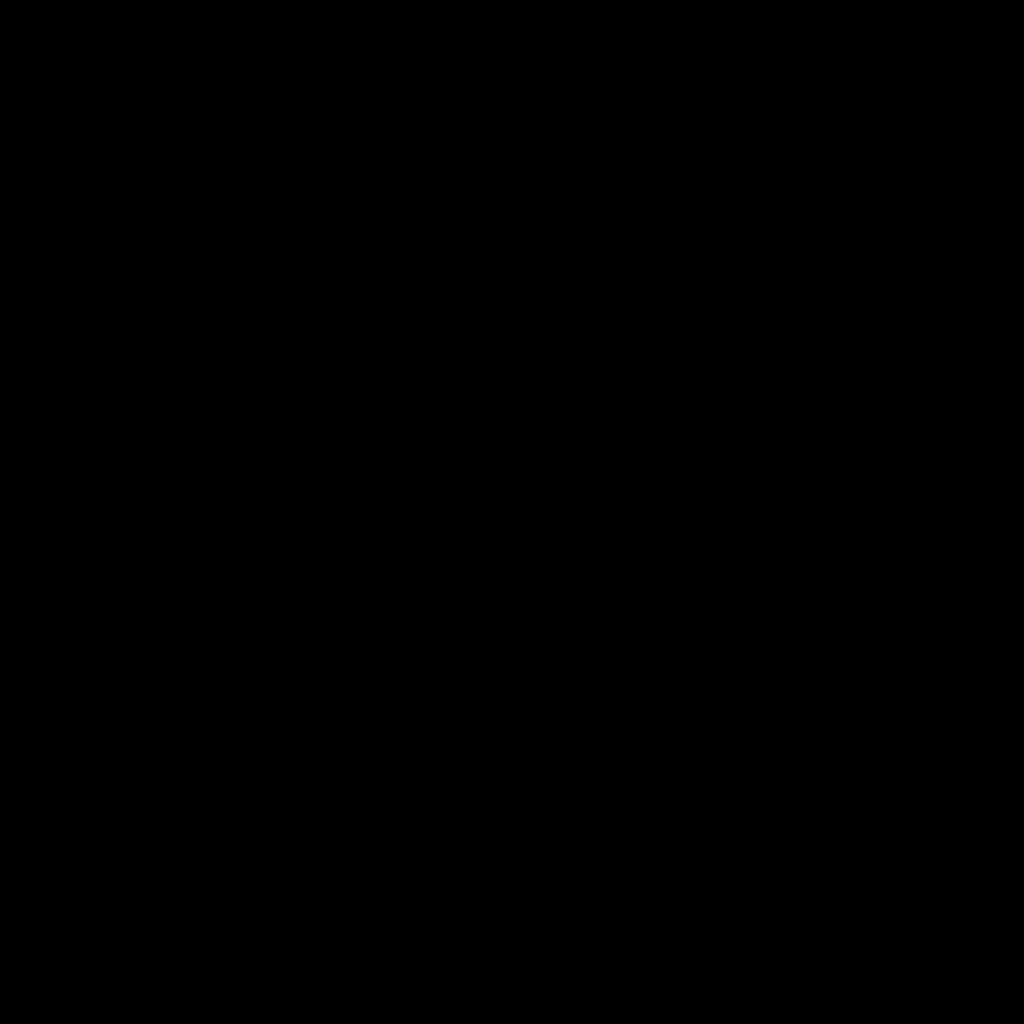

Supplement: Supplementary file 9 — EV and Appendix Figure Source Data [file 44318_2024_357_MOESM9_ESM.zip › FigEV-Appendix/Appendix Fig.S3/S3A/GmNPF7.5-YFPC+YFPN/Overlay.tif]

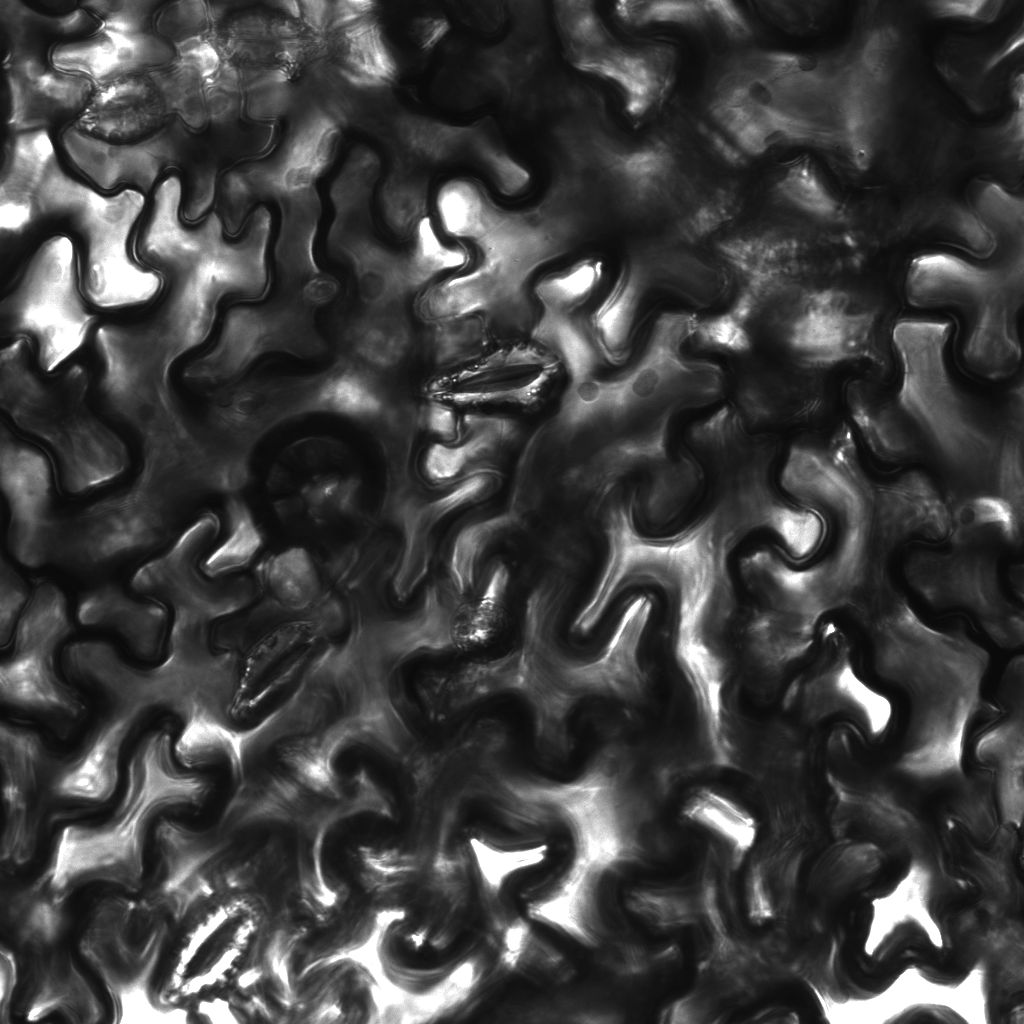

Supplement: Supplementary file 9 — EV and Appendix Figure Source Data [file 44318_2024_357_MOESM9_ESM.zip › FigEV-Appendix/Appendix Fig.S3/S3A/GmNPF7.5-YFPC+YFPN/YFP.tif]

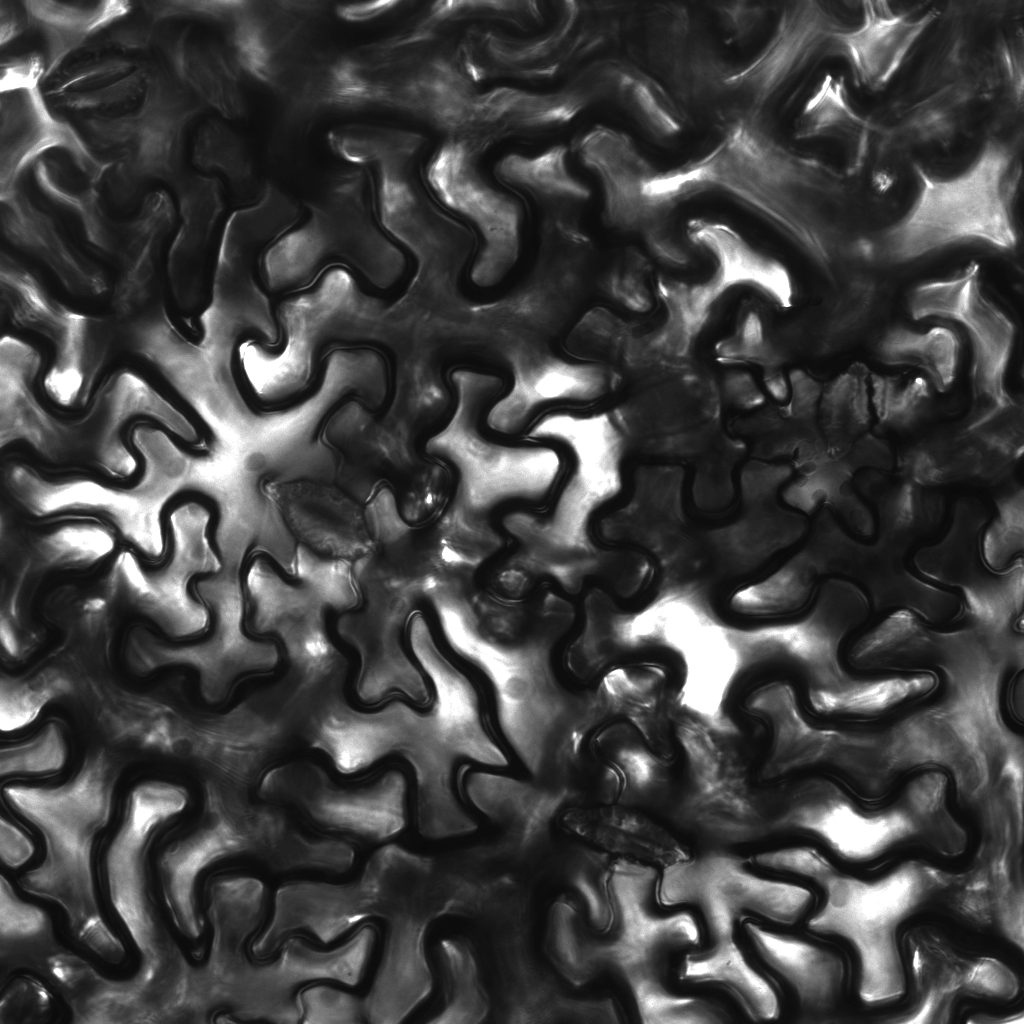

Supplement: Supplementary file 9 — EV and Appendix Figure Source Data [file 44318_2024_357_MOESM9_ESM.zip › FigEV-Appendix/Appendix Fig.S3/S3A/YFPC+GmPI4Kγ4-YFPN/Overlay.tif]

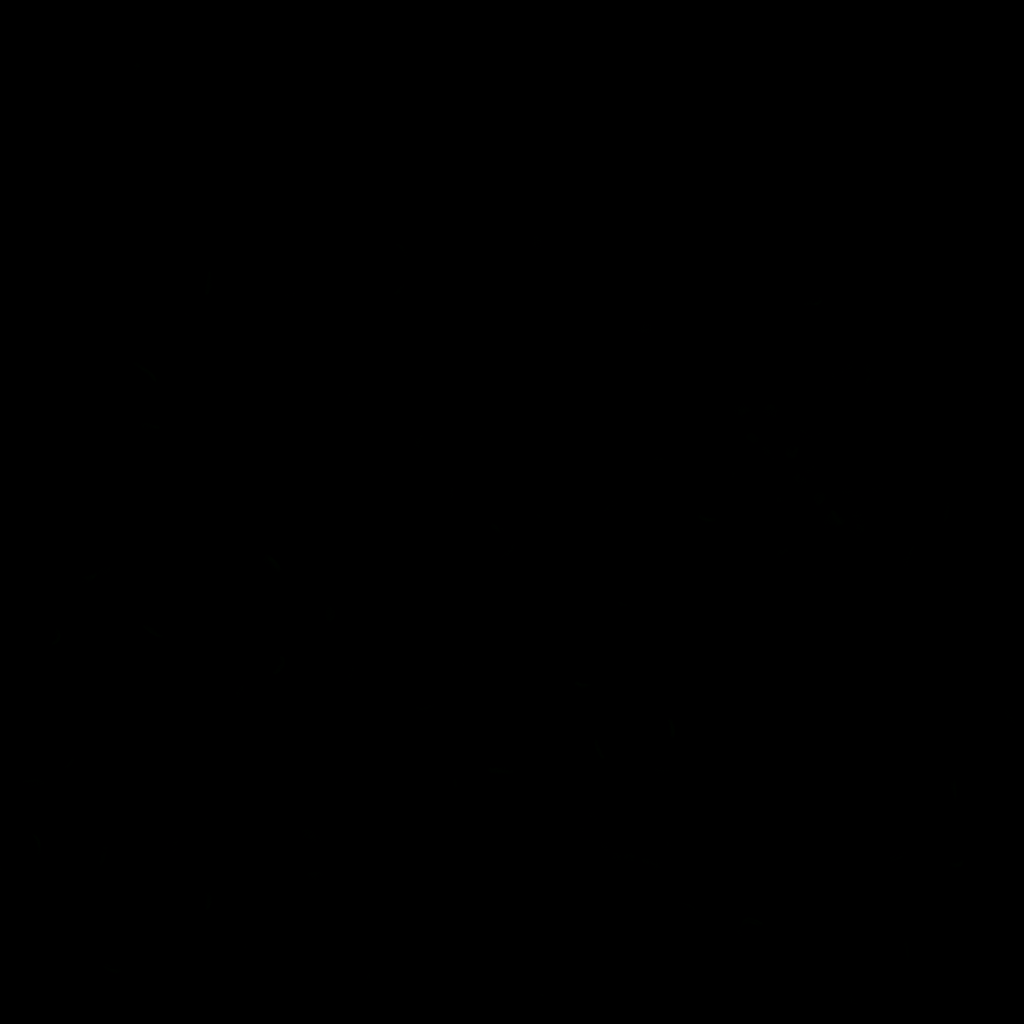

Supplement: Supplementary file 9 — EV and Appendix Figure Source Data [file 44318_2024_357_MOESM9_ESM.zip › FigEV-Appendix/Appendix Fig.S3/S3A/YFPC+GmPI4Kγ4-YFPN/YFP.tif]

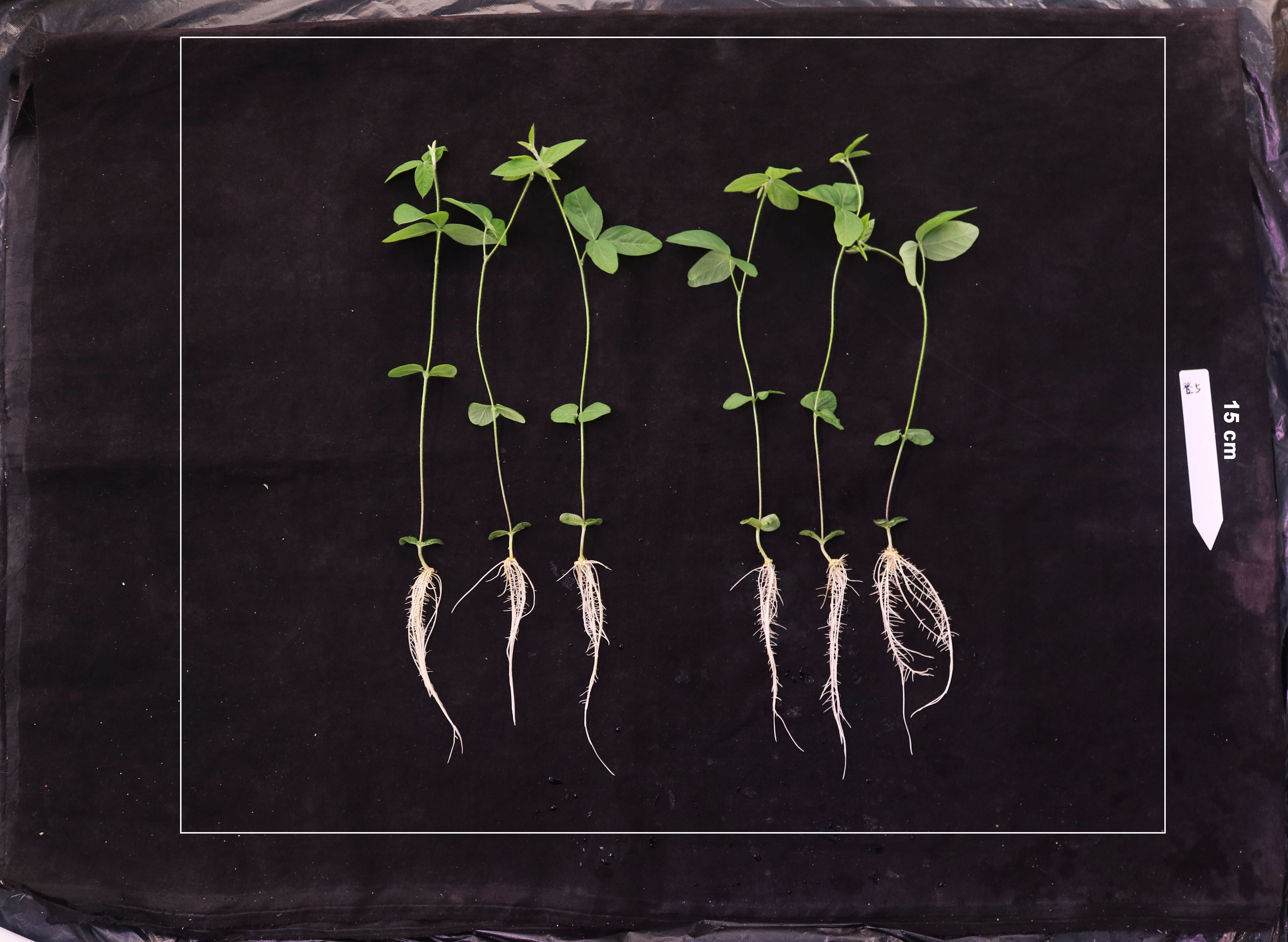

Supplement: Supplementary file 9 — EV and Appendix Figure Source Data [file 44318_2024_357_MOESM9_ESM.zip › FigEV-Appendix/Appendix Fig.S8/S8A/Control.jpg]

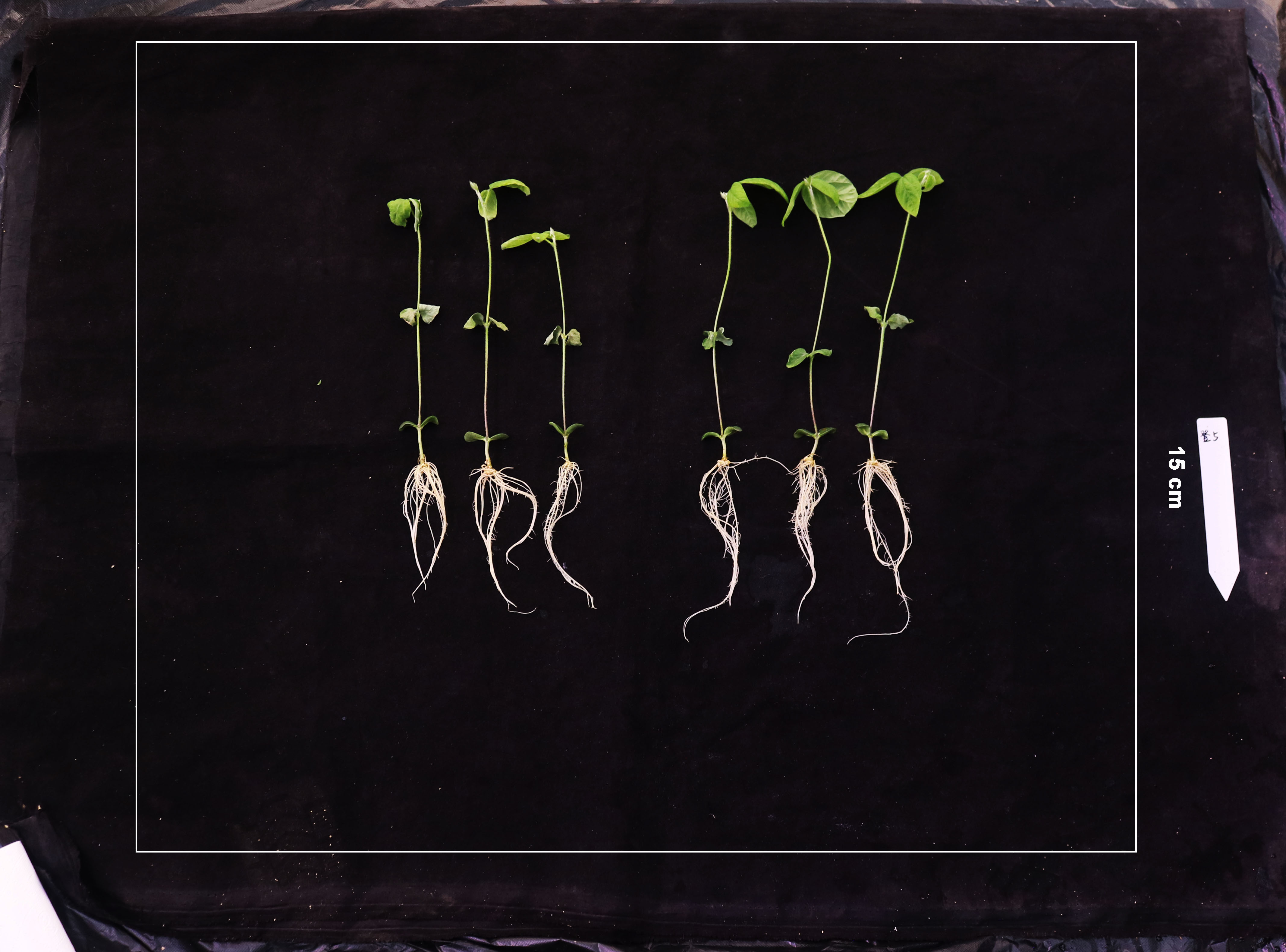

Supplement: Supplementary file 9 — EV and Appendix Figure Source Data [file 44318_2024_357_MOESM9_ESM.zip › FigEV-Appendix/Appendix Fig.S8/S8A/NaCl.jpg]

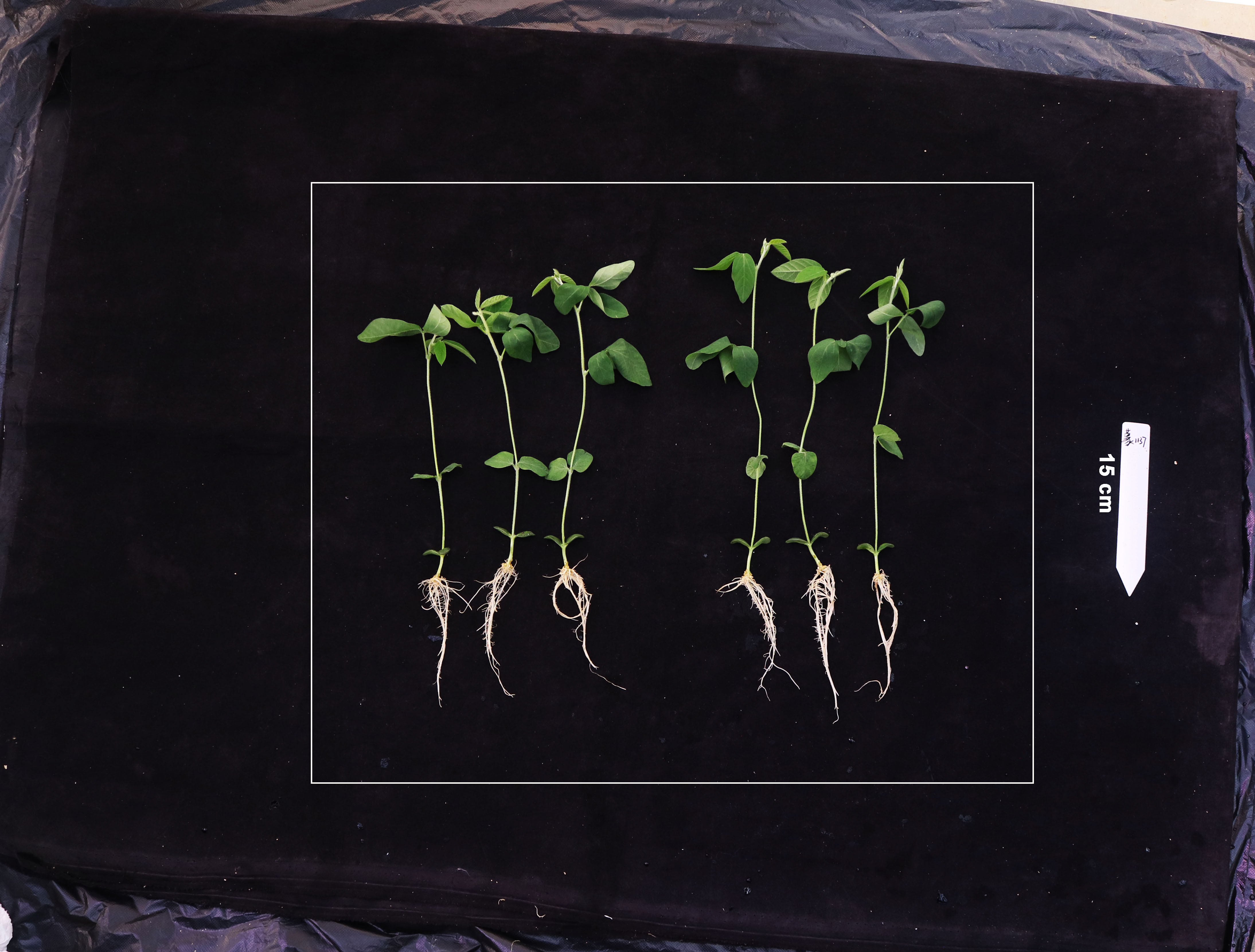

Supplement: Supplementary file 9 — EV and Appendix Figure Source Data [file 44318_2024_357_MOESM9_ESM.zip › FigEV-Appendix/Appendix Fig.S8/S8B/Control.jpg]

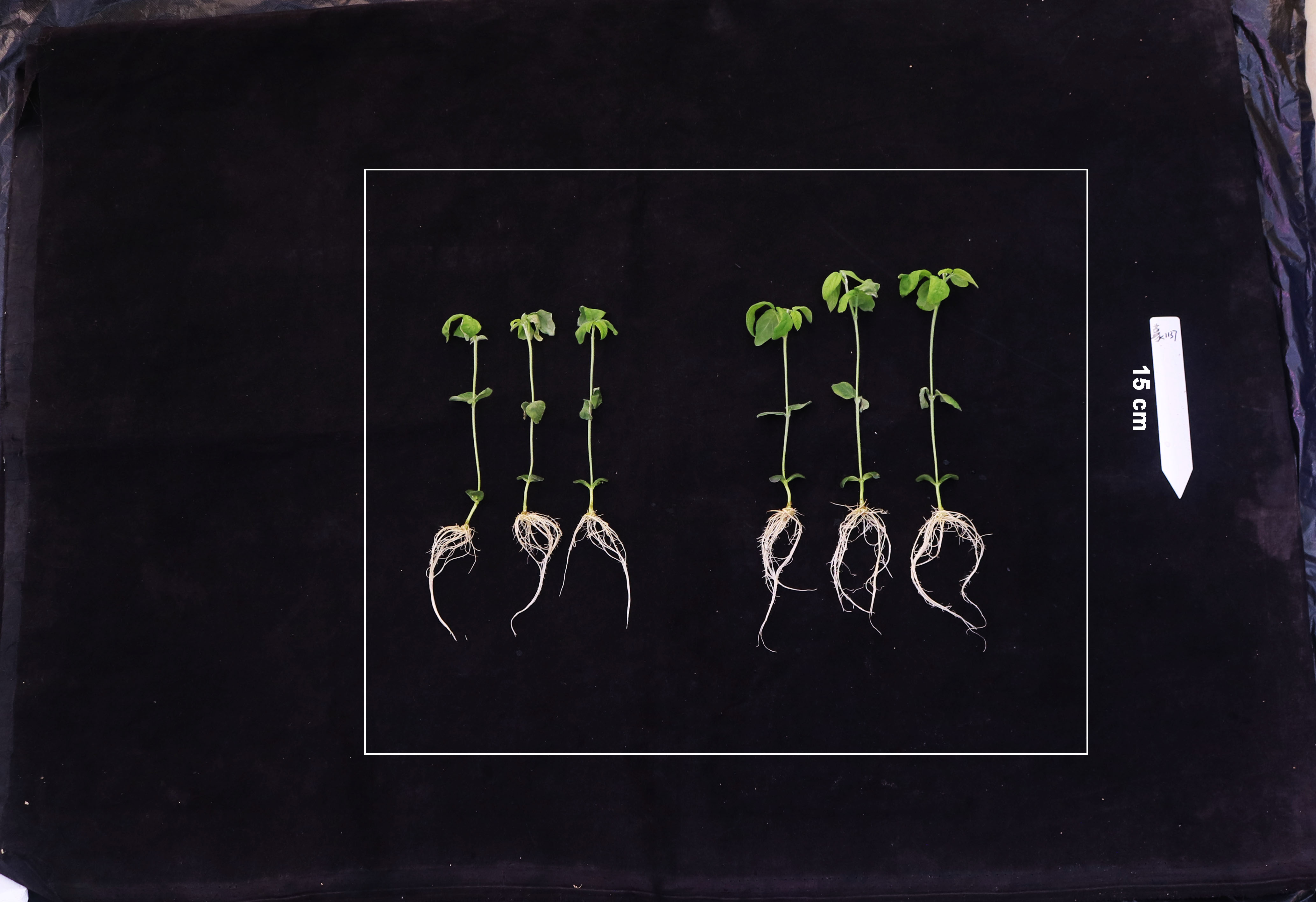

Supplement: Supplementary file 9 — EV and Appendix Figure Source Data [file 44318_2024_357_MOESM9_ESM.zip › FigEV-Appendix/Appendix Fig.S8/S8B/NaCl.jpg]

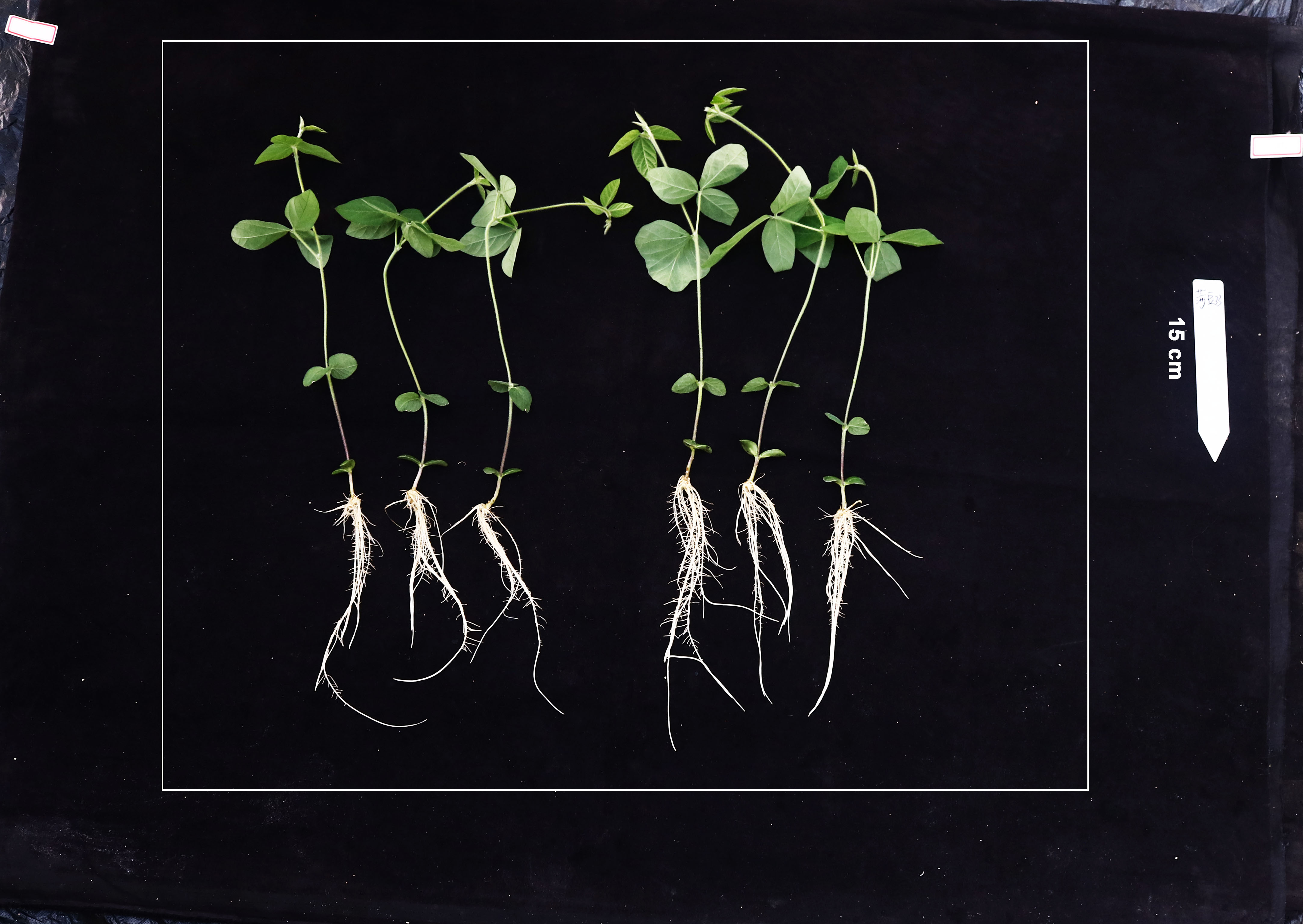

Supplement: Supplementary file 9 — EV and Appendix Figure Source Data [file 44318_2024_357_MOESM9_ESM.zip › FigEV-Appendix/Appendix Fig.S8/S8C/Control.jpg]

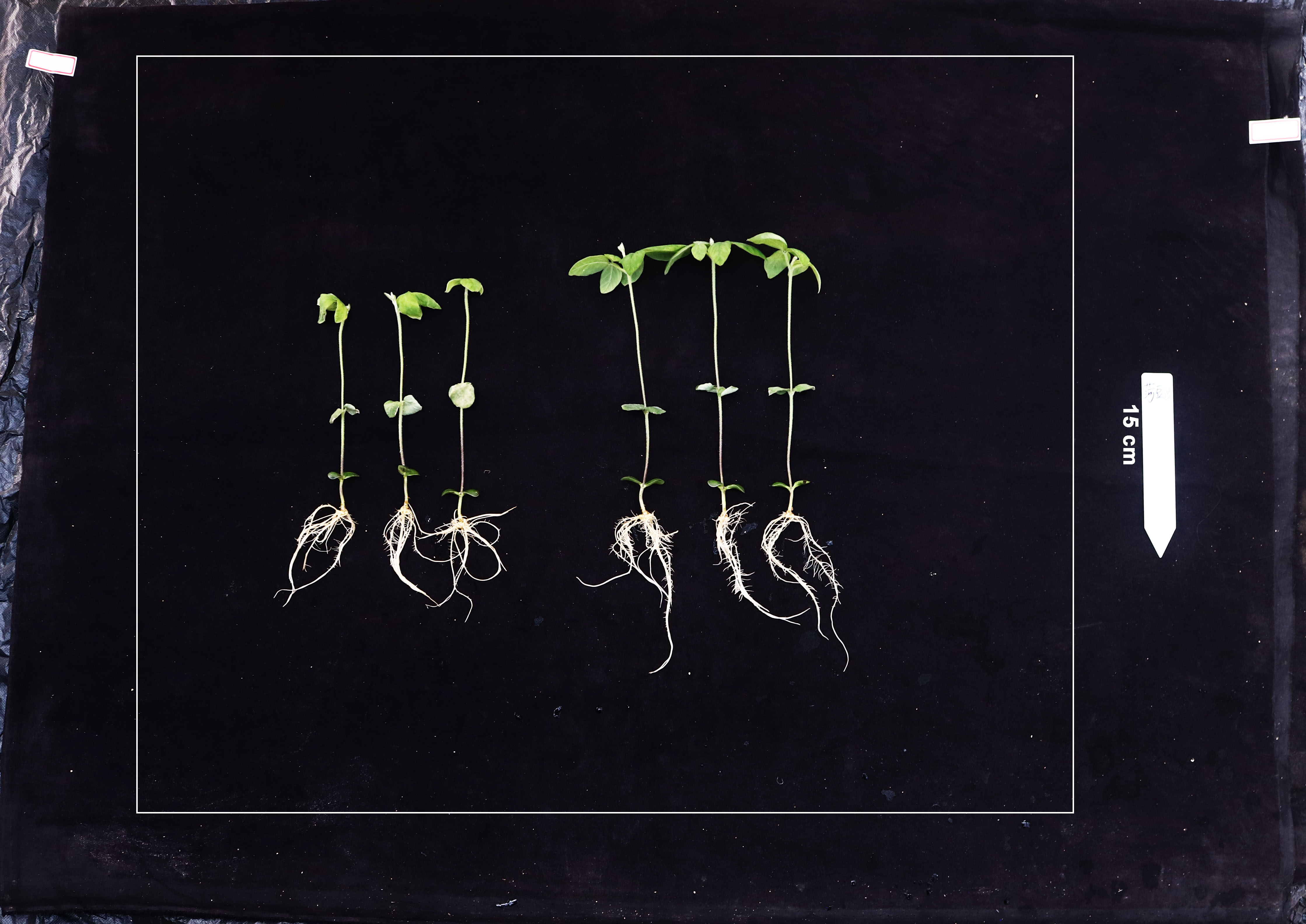

Supplement: Supplementary file 9 — EV and Appendix Figure Source Data [file 44318_2024_357_MOESM9_ESM.zip › FigEV-Appendix/Appendix Fig.S8/S8C/NaCl.jpg]

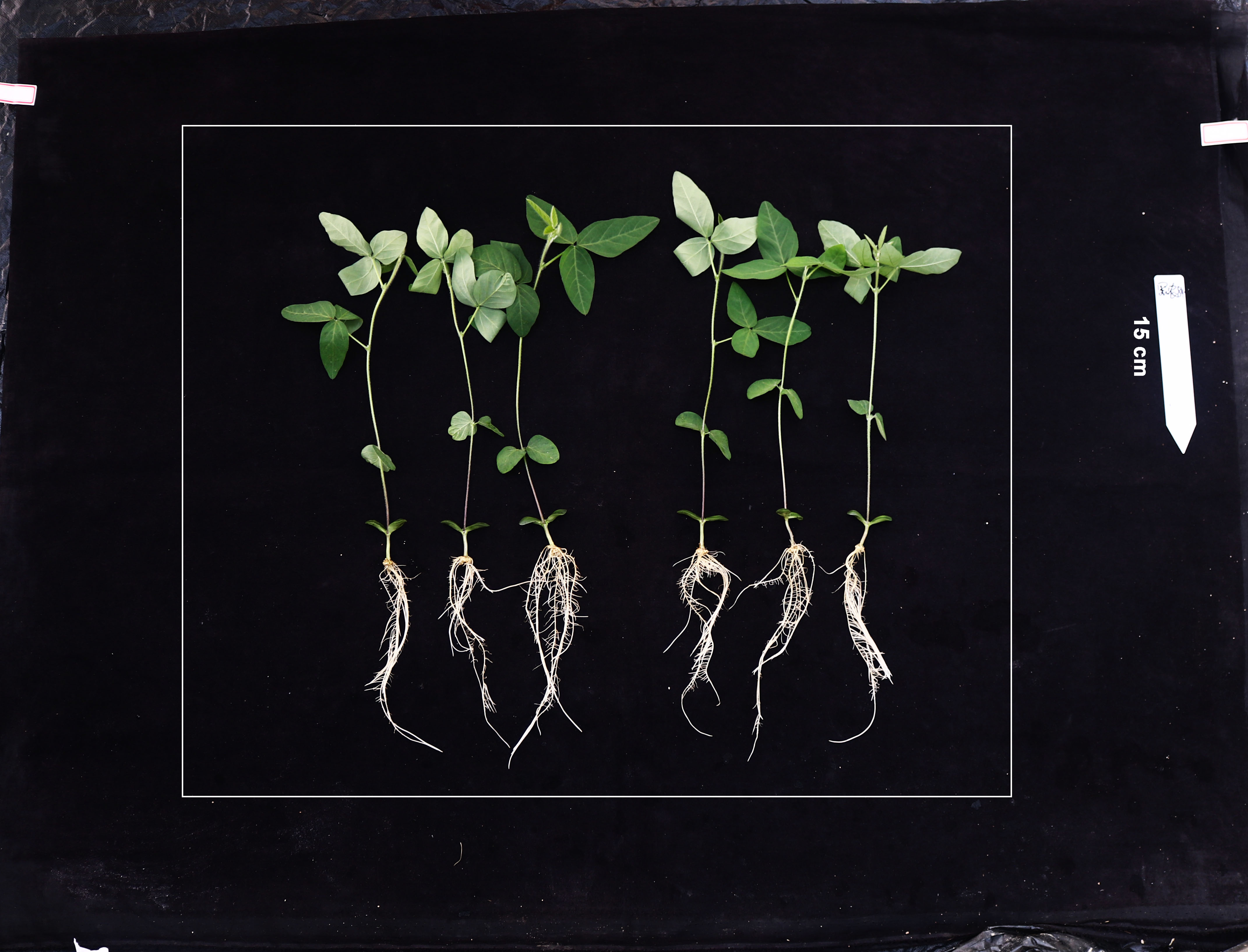

Supplement: Supplementary file 9 — EV and Appendix Figure Source Data [file 44318_2024_357_MOESM9_ESM.zip › FigEV-Appendix/Appendix Fig.S8/S8D/Control.jpg]

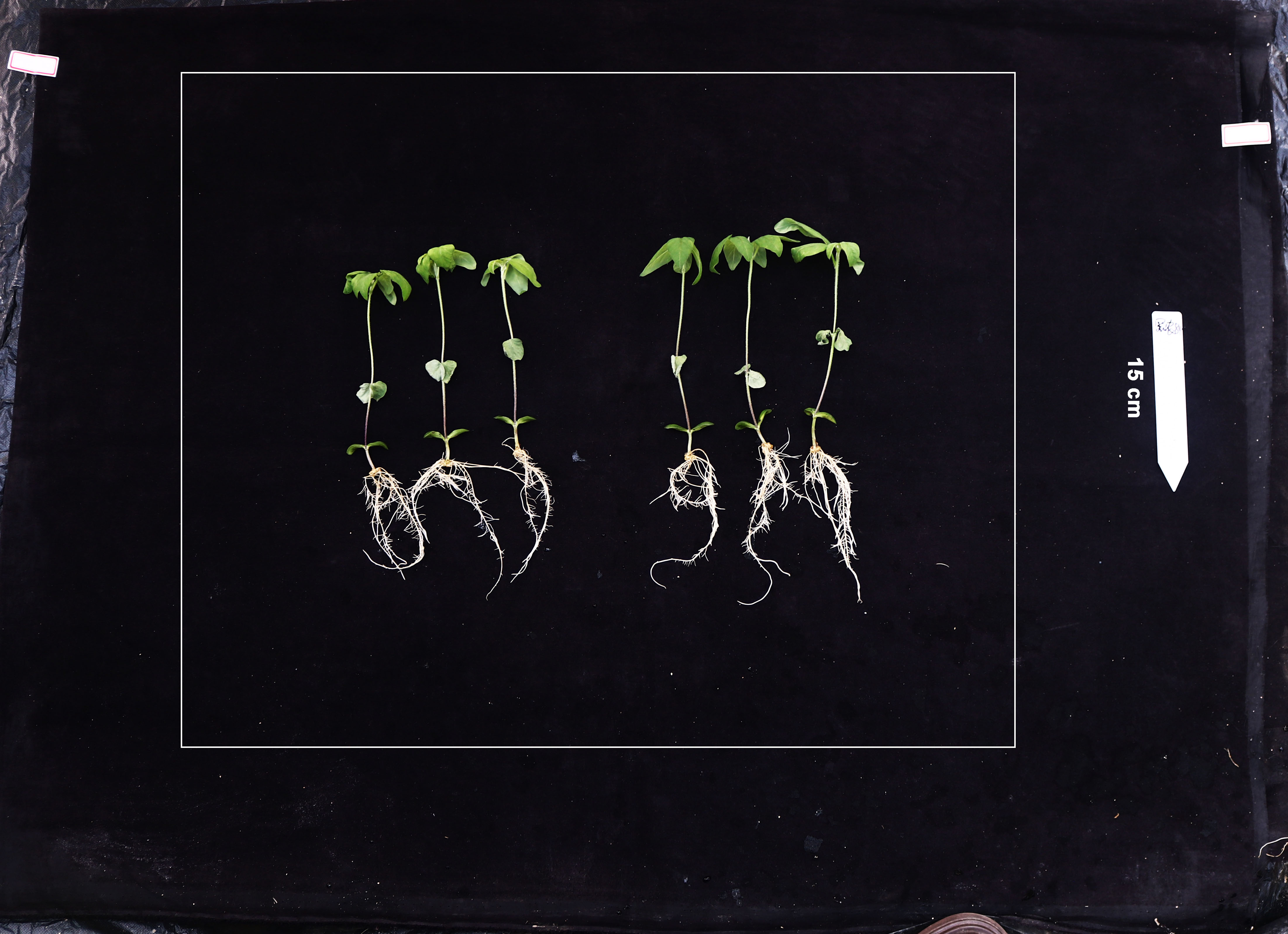

Supplement: Supplementary file 9 — EV and Appendix Figure Source Data [file 44318_2024_357_MOESM9_ESM.zip › FigEV-Appendix/Appendix Fig.S8/S8D/NaCl.jpg]

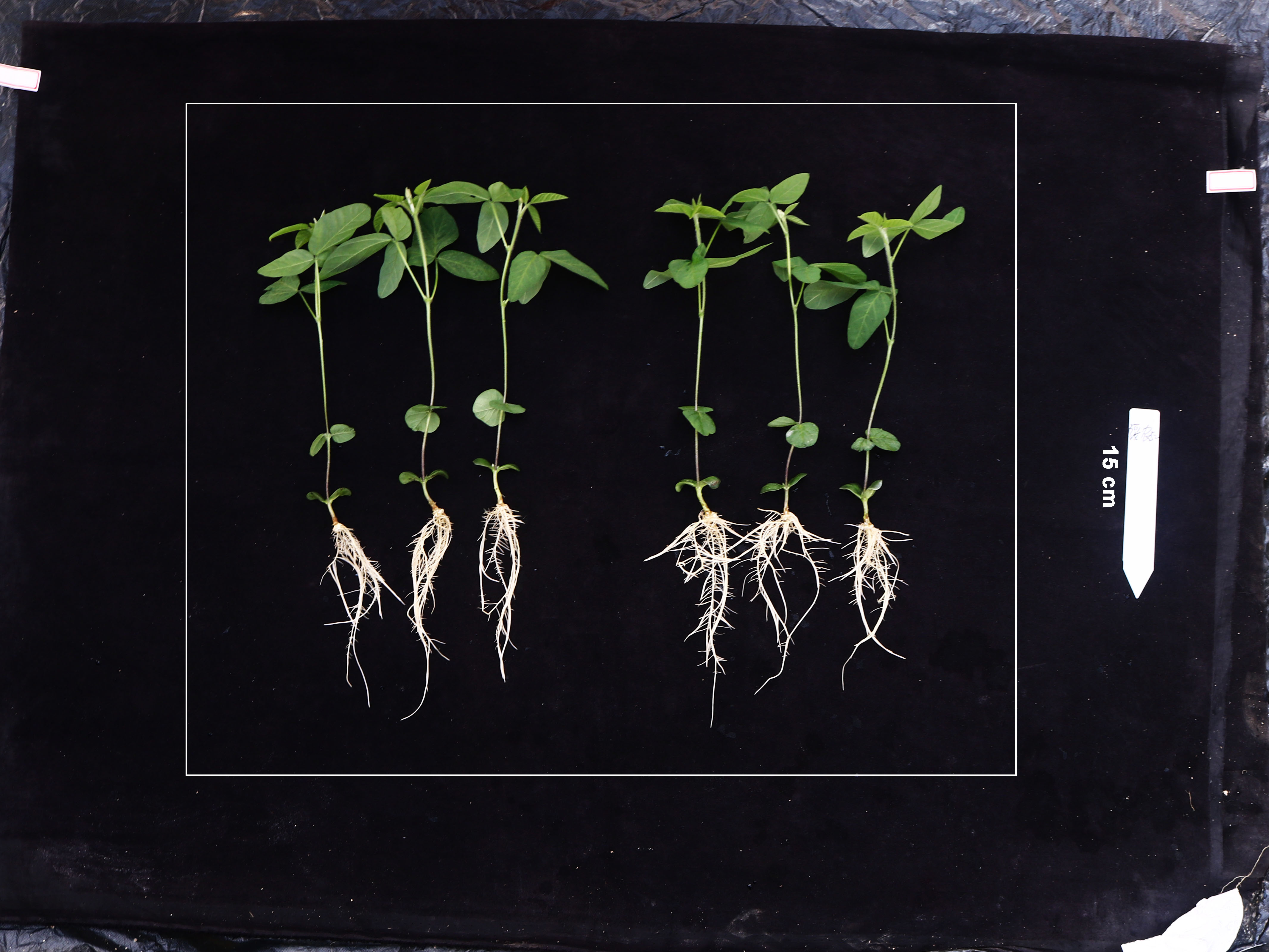

Supplement: Supplementary file 9 — EV and Appendix Figure Source Data [file 44318_2024_357_MOESM9_ESM.zip › FigEV-Appendix/Appendix Fig.S8/S8E/Control.jpg]

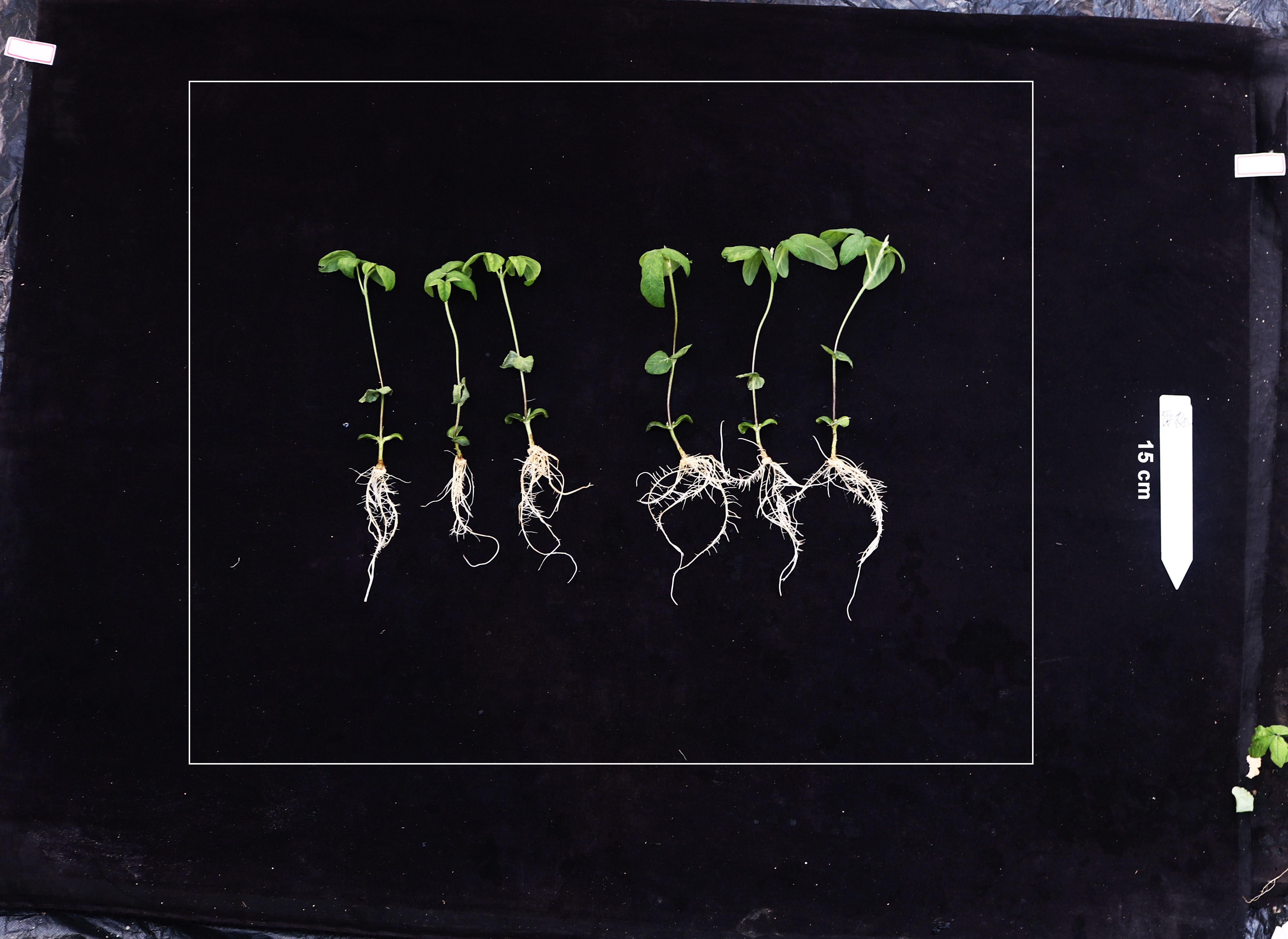

Supplement: Supplementary file 9 — EV and Appendix Figure Source Data [file 44318_2024_357_MOESM9_ESM.zip › FigEV-Appendix/Appendix Fig.S8/S8E/NaCl.jpg]

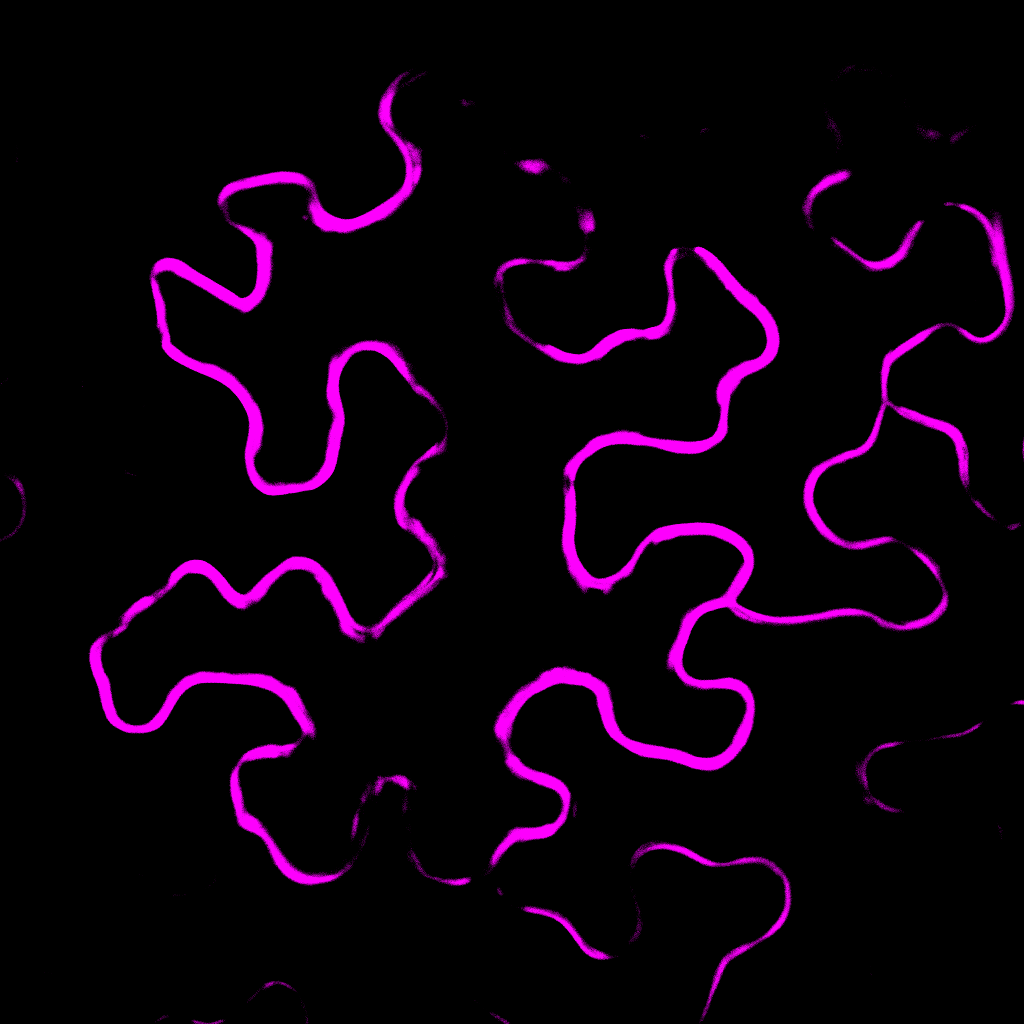

Supplement: Supplementary file 9 — EV and Appendix Figure Source Data [file 44318_2024_357_MOESM9_ESM.zip › FigEV-Appendix/FigEV3/EV3A/GFP/AtCBL1n-mCherry.tif]

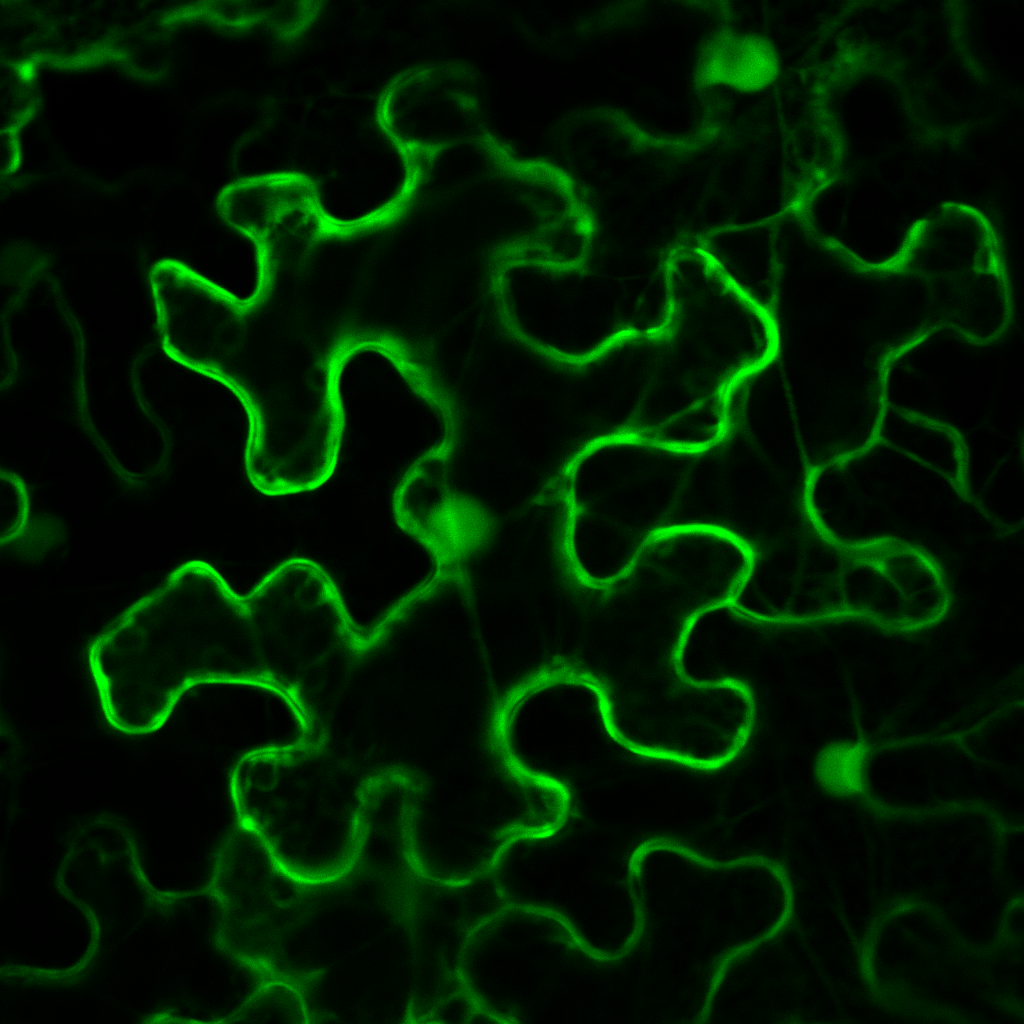

Supplement: Supplementary file 9 — EV and Appendix Figure Source Data [file 44318_2024_357_MOESM9_ESM.zip › FigEV-Appendix/FigEV3/EV3A/GFP/GFP.tif]

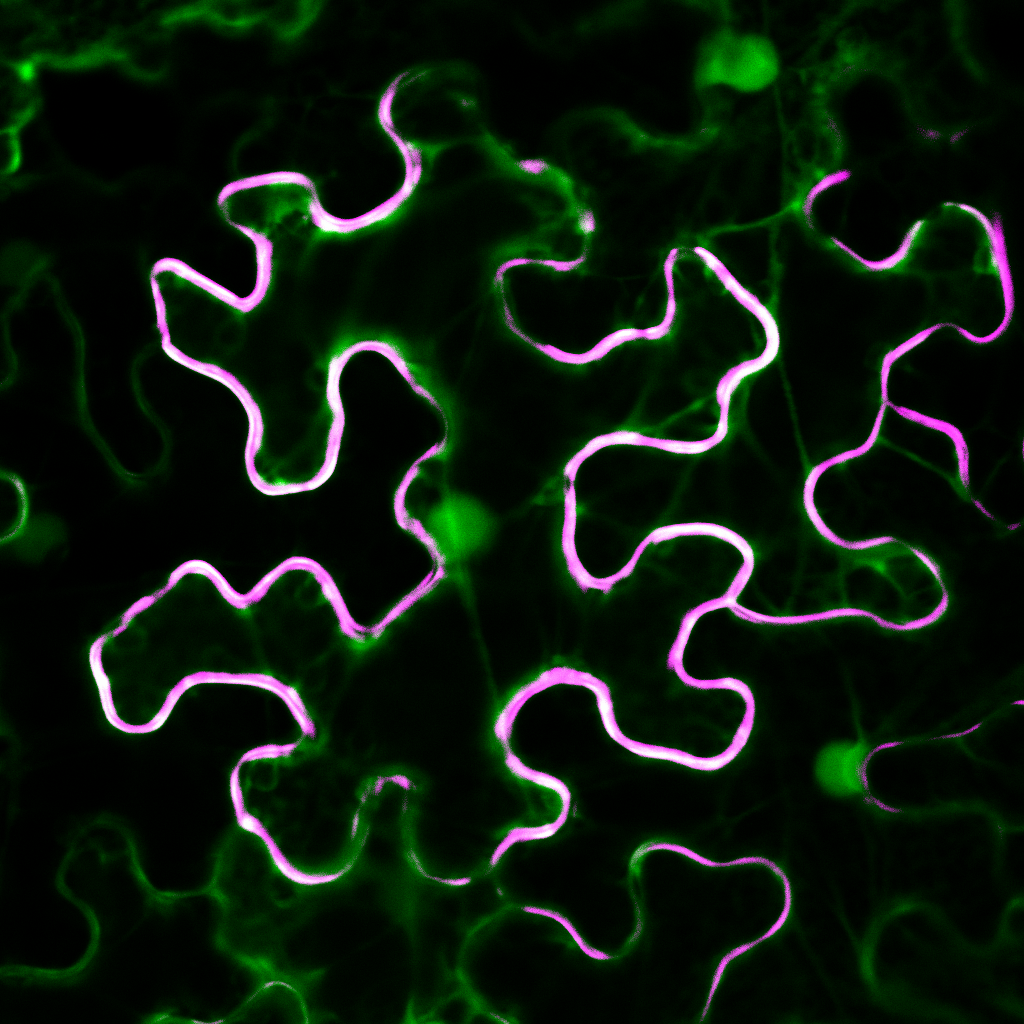

Supplement: Supplementary file 9 — EV and Appendix Figure Source Data [file 44318_2024_357_MOESM9_ESM.zip › FigEV-Appendix/FigEV3/EV3A/GFP/Merge.tif]

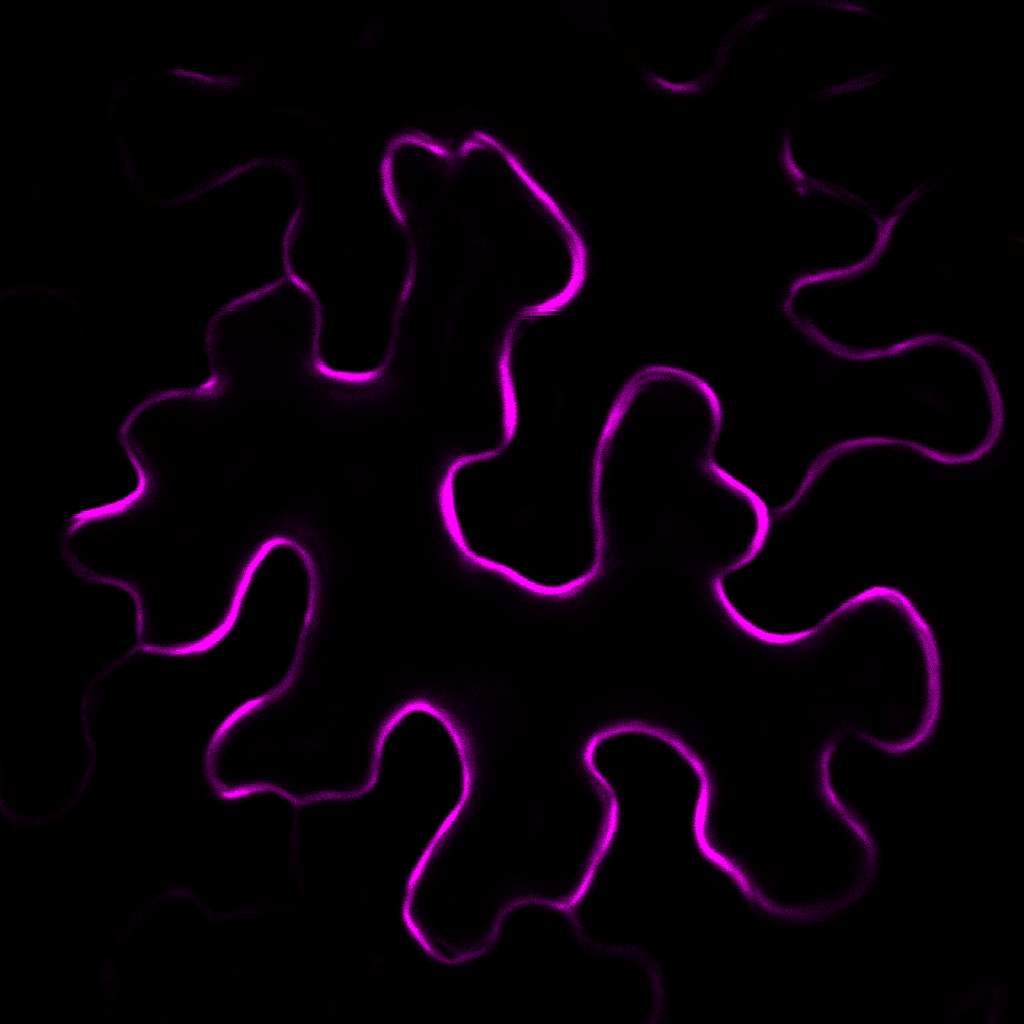

Supplement: Supplementary file 9 — EV and Appendix Figure Source Data [file 44318_2024_357_MOESM9_ESM.zip › FigEV-Appendix/FigEV3/EV3A/GmNPF7.5-GFP/AtCBL1n-mCherry.tif]

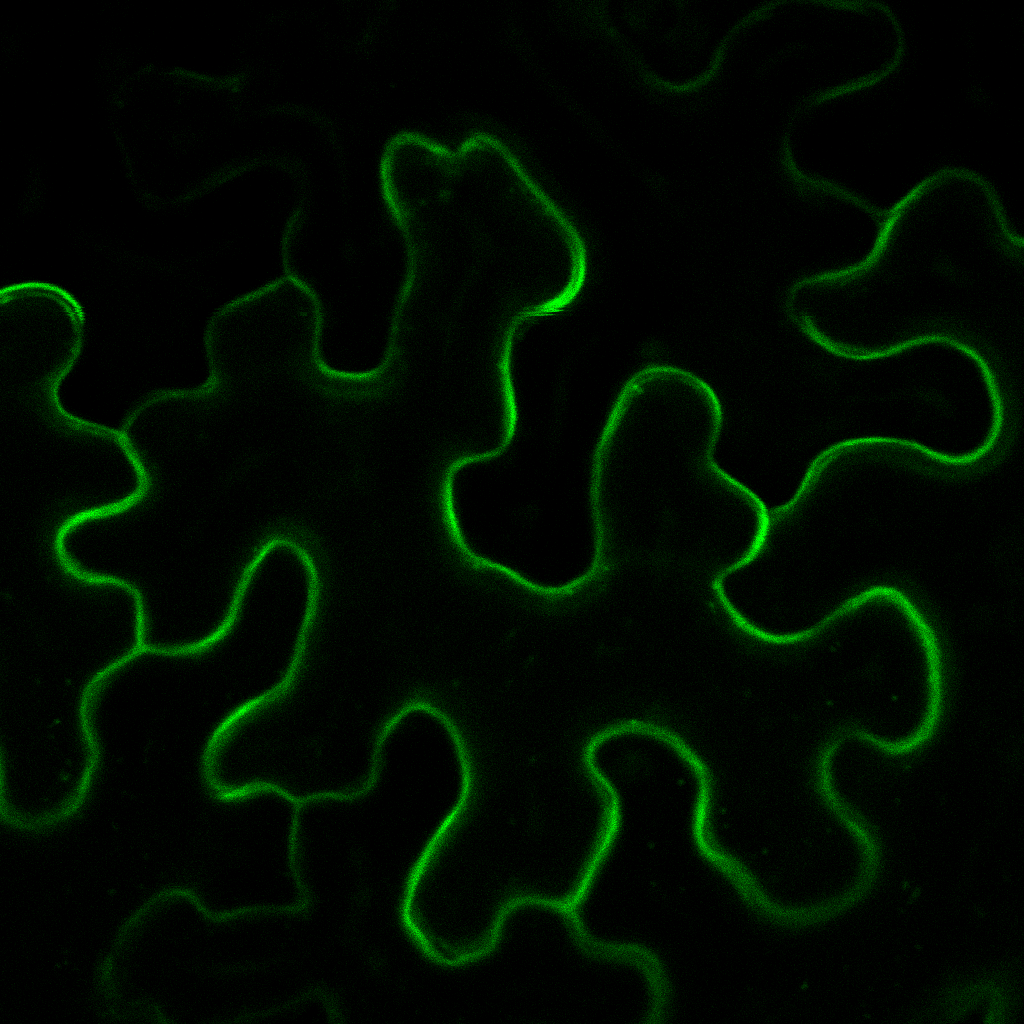

Supplement: Supplementary file 9 — EV and Appendix Figure Source Data [file 44318_2024_357_MOESM9_ESM.zip › FigEV-Appendix/FigEV3/EV3A/GmNPF7.5-GFP/GmNPF7.5-GFP.tif]

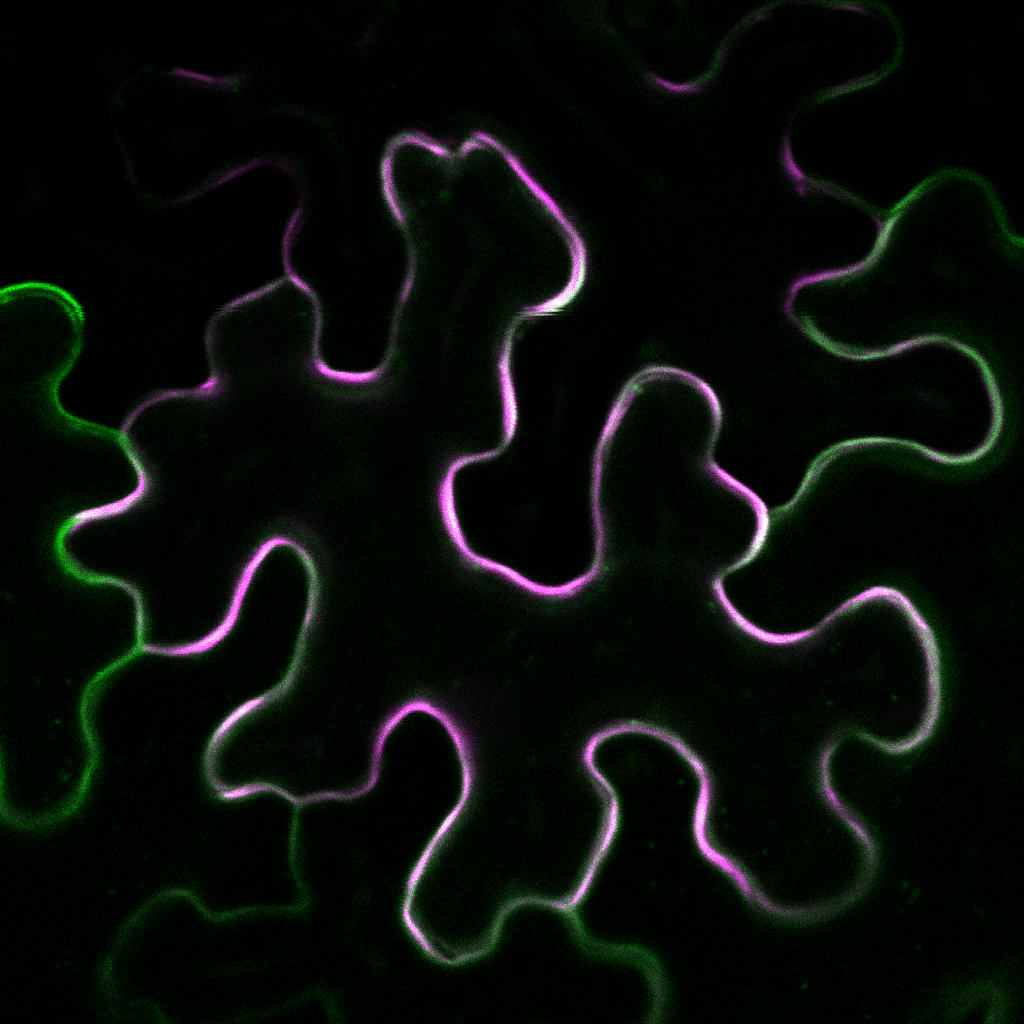

Supplement: Supplementary file 9 — EV and Appendix Figure Source Data [file 44318_2024_357_MOESM9_ESM.zip › FigEV-Appendix/FigEV3/EV3A/GmNPF7.5-GFP/Merge.tif]

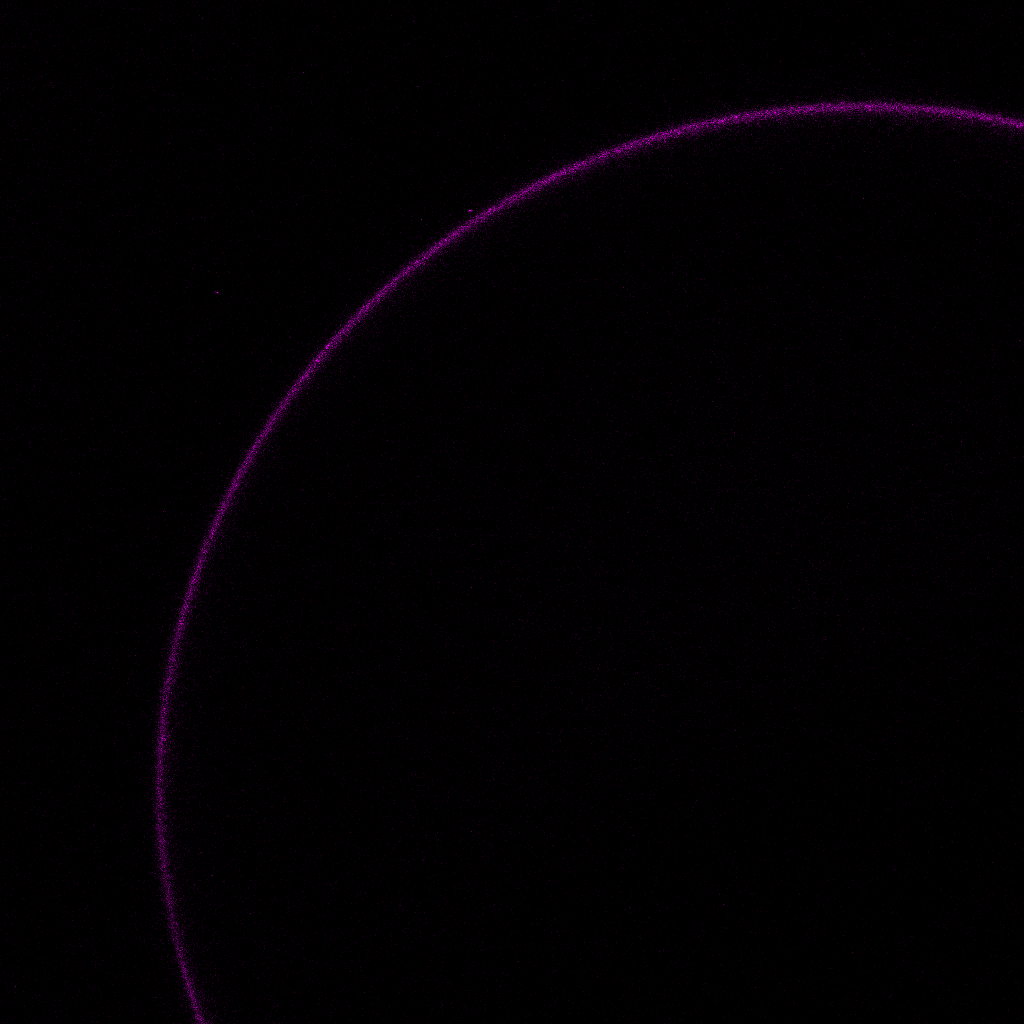

Supplement: Supplementary file 9 — EV and Appendix Figure Source Data [file 44318_2024_357_MOESM9_ESM.zip › FigEV-Appendix/FigEV3/EV3B/GmNPF7.5-GFP/FM 4-64.tif]

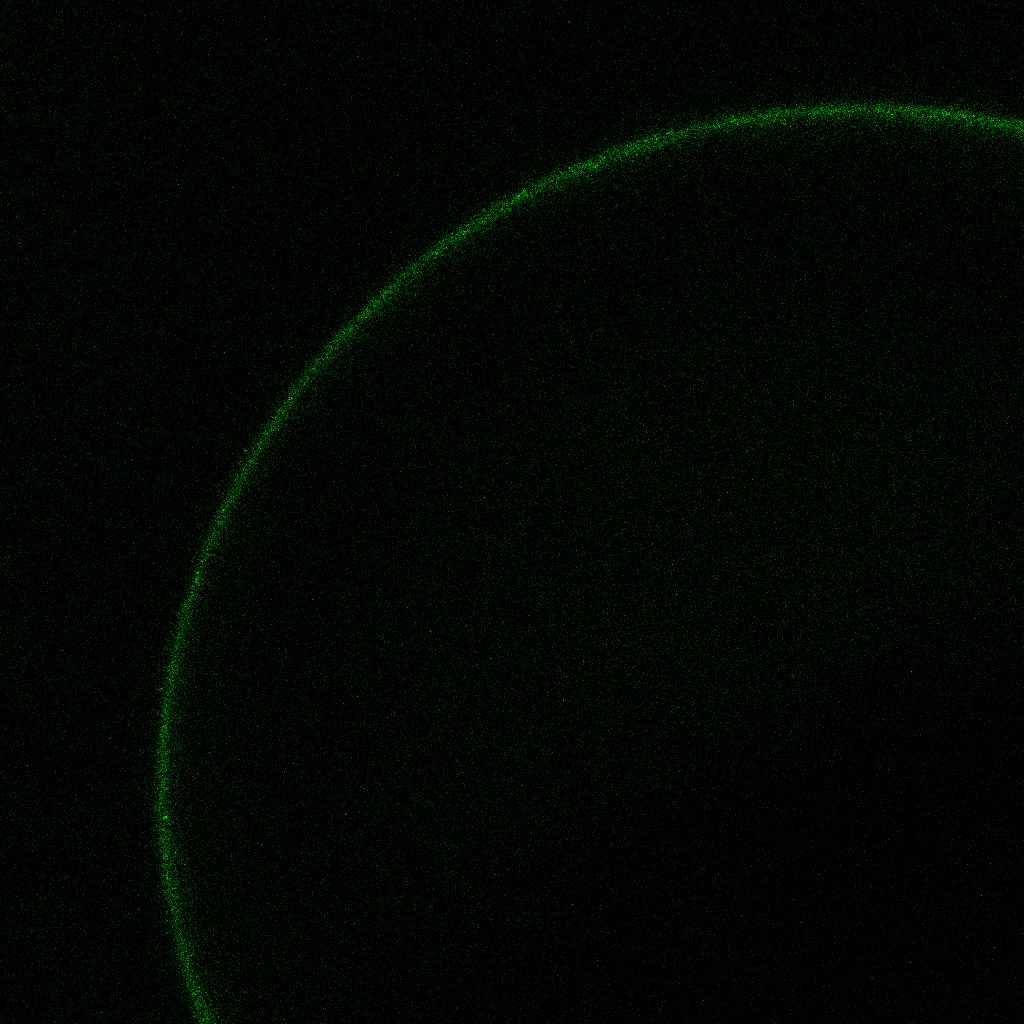

Supplement: Supplementary file 9 — EV and Appendix Figure Source Data [file 44318_2024_357_MOESM9_ESM.zip › FigEV-Appendix/FigEV3/EV3B/GmNPF7.5-GFP/GmNPF7.5GFP.tif]

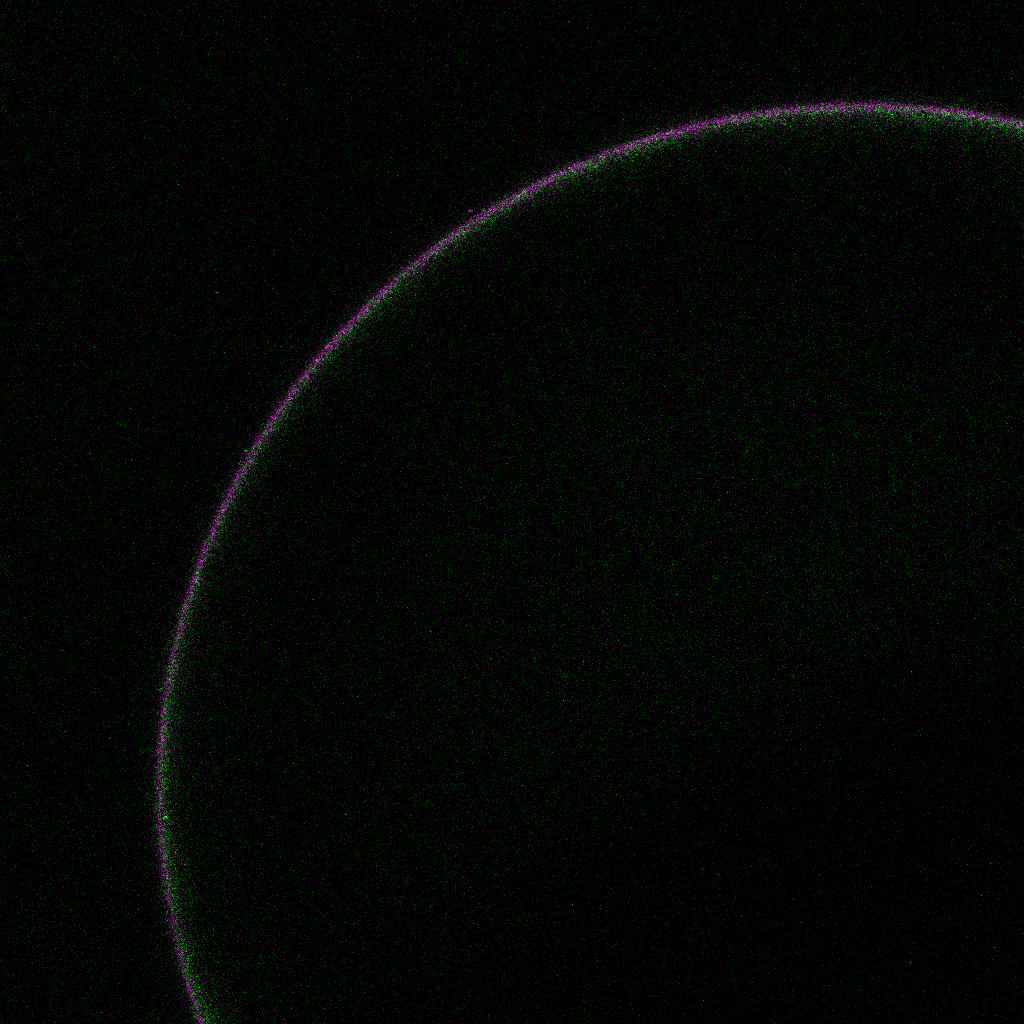

Supplement: Supplementary file 9 — EV and Appendix Figure Source Data [file 44318_2024_357_MOESM9_ESM.zip › FigEV-Appendix/FigEV3/EV3B/GmNPF7.5-GFP/Merge.tif]

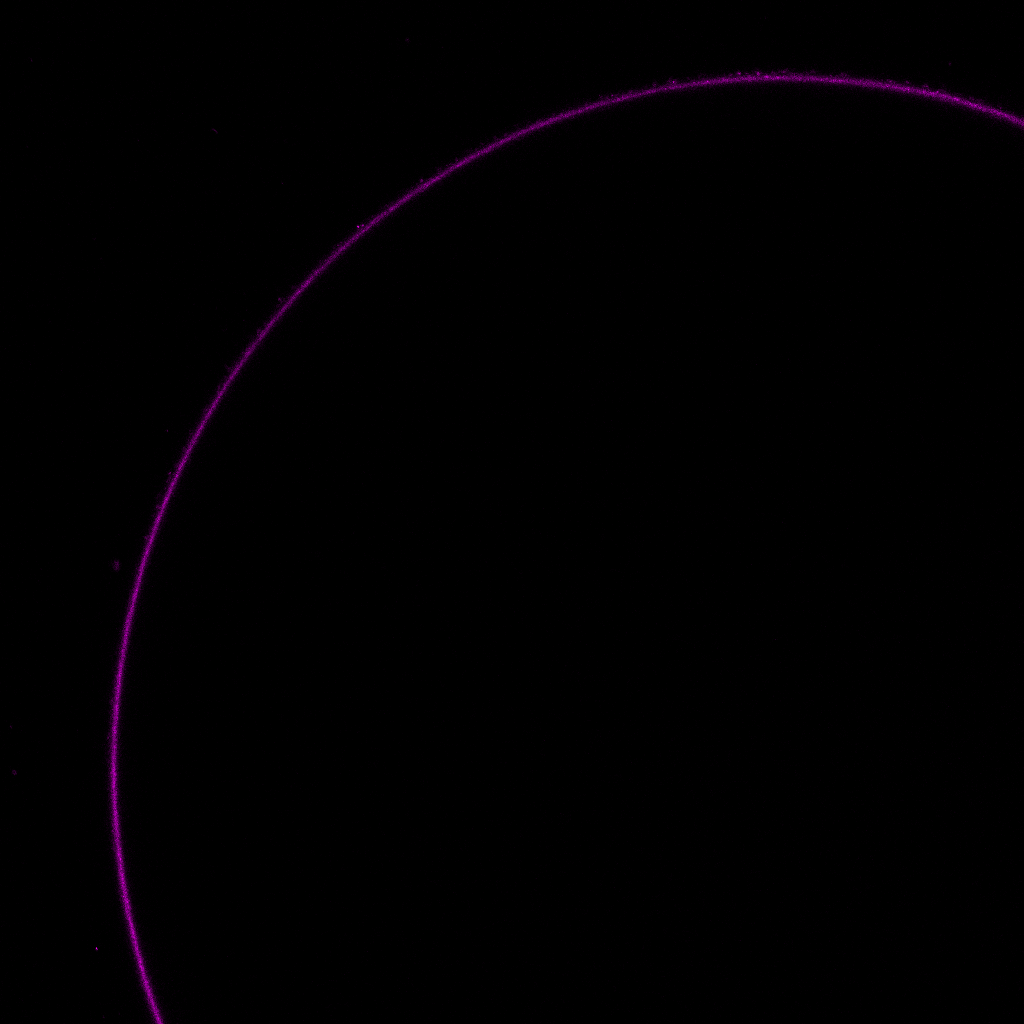

Supplement: Supplementary file 9 — EV and Appendix Figure Source Data [file 44318_2024_357_MOESM9_ESM.zip › FigEV-Appendix/FigEV3/EV3B/Water/FM 4-64.tif]

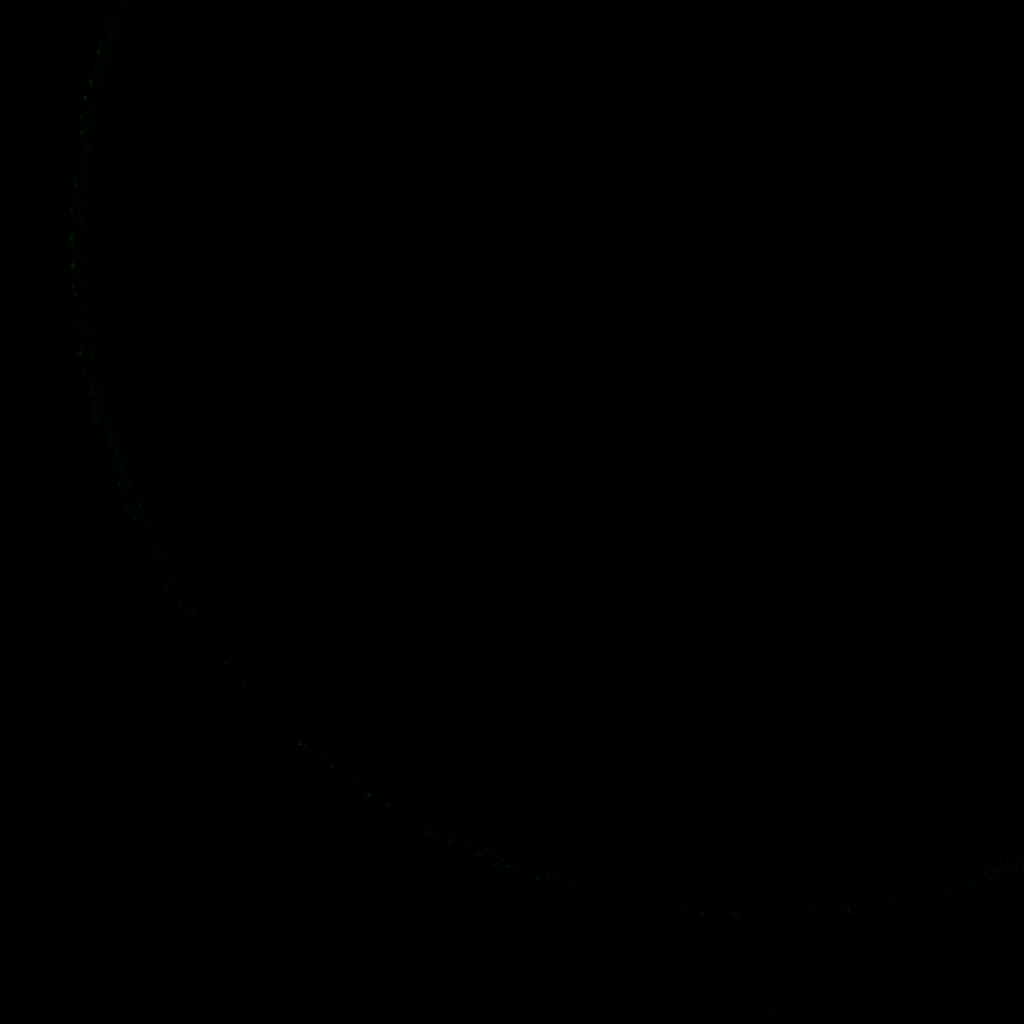

Supplement: Supplementary file 9 — EV and Appendix Figure Source Data [file 44318_2024_357_MOESM9_ESM.zip › FigEV-Appendix/FigEV3/EV3B/Water/GFP.tif]

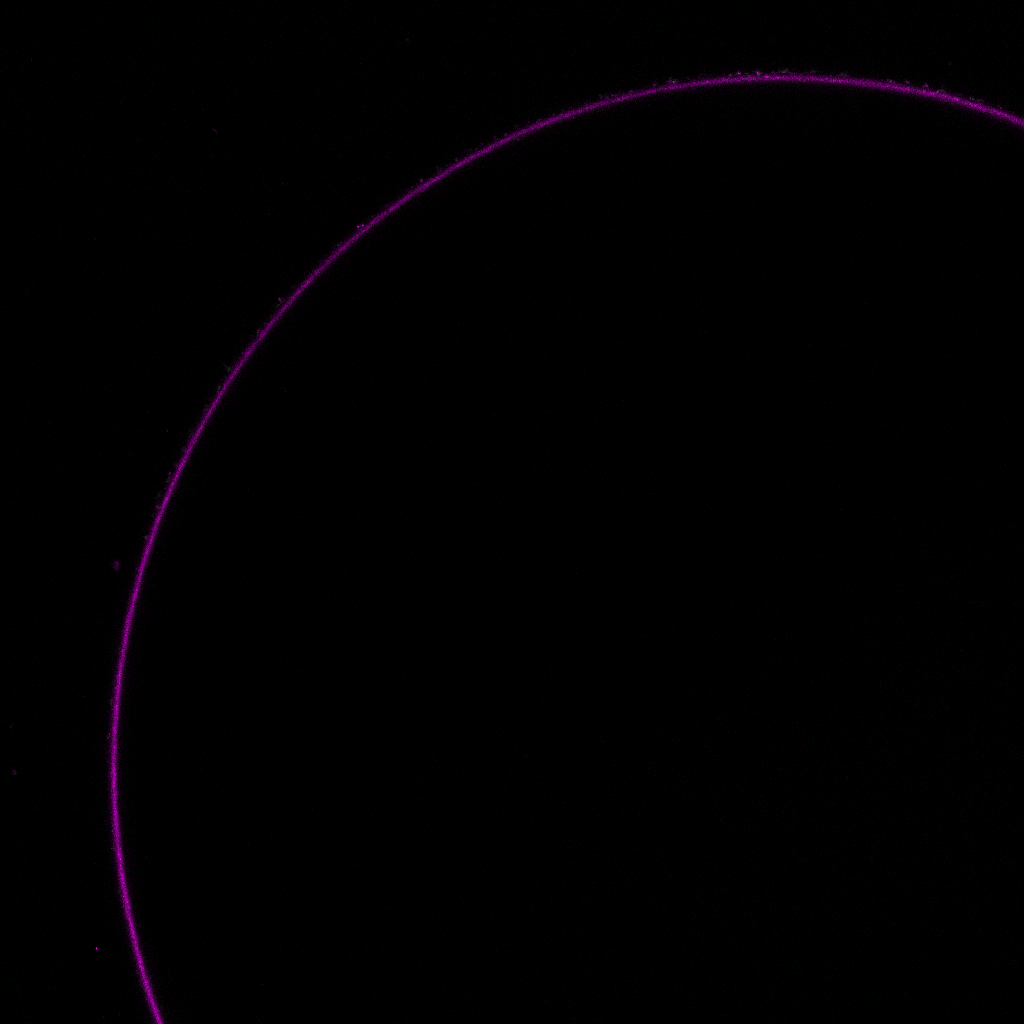

Supplement: Supplementary file 9 — EV and Appendix Figure Source Data [file 44318_2024_357_MOESM9_ESM.zip › FigEV-Appendix/FigEV3/EV3B/Water/Merge.tif]
