# Supplementary material for: A phosphorylation-regulated NPF transporter determines salt tolerance by mediating chloride uptake in soybean plants
Source: EMBO J. 2025 Jan 3;44(3):923–46. doi: 10.1038/s44318-024-00357-1 (PMC11790925; doi:10.1038/s44318-024-00357-1)
Supplement: Supplementary file 10 — Expanded View Figures [file 44318_2024_357_MOESM10_ESM.pdf]

Expanded View Figures

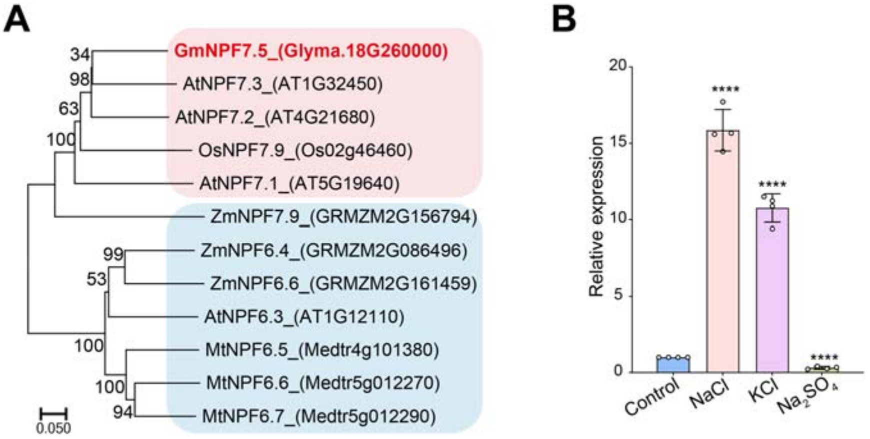

Figure EV1. Phylogenetic tree and transcript levels of *GmNPF7.5* in soybean plants under indicated conditions.

(**A**) Phylogenic analysis of NPF6 and NPF7 from *Arabidopsis*, rice, maize, and soybean. The phylogenetic tree was constructed using MEGA7. (**B**) Transcript levels of *GmNPF7.5* in soybean plants under the indicated conditions. *GmELF* was used as an internal control. Data are means ± standard error (SEM). Significance was determined using a two-sided Student's *t* test (****$P < 0.0001$), $n = 4$ (4 biological replicates). *P* values < 0.0001 (NaCl vs Control), <0.0001 (KCl vs Control), <0.0001 (Na_2SO_4 vs Control). Source data are available online for this figure.

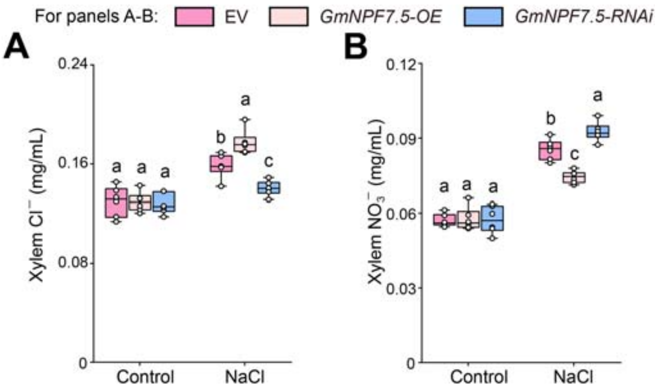

Figure EV2. Comparison of Cl^- and NO_3^- content levels in xylem sap of transgenic soybean lines with hairy roots harboring the indicated constructs.

(**A**, **B**) Cl^- concentration (**A**), NO_3^- concentration (**B**) in xylem sap of *GmNPF7.5* overexpression (*GmNPF7.5*-OE) or knockdown (*GmNPF7.5*-RNAi) hairy root transgenic soybean lines. Data in (**A**, **B**) are means ± SEM ($n = 6$ independent biological replicates). Significance was determined using one-way ANOVA, followed by Tukey's test. Different letters indicate significant differences ($P < 0.05$). (**A**) Control: *P* values = 0.9992 (EV vs *GmNPF7.5*-OE), 0.9411 (EV vs *GmNPF7.5*-RNAi), 0.9535 (*GmNPF7.5*-OE vs *GmNPF7.5*-RNAi). (The following is the same order). NaCl: *P* values = 0.0034, 0.0051, <0.0001. (**B**) Control: *P* values = 0.9729, 0.9886, 0.9965. NaCl: *P* values = 0.0001, 0.0116, <0.0001. For box plots: the whiskers represent maximum and minimum values, and boxes represent he upper quartile, median, and lower quartile, dots represent data points. Source data are available online for this figure.

 © The Author(s)

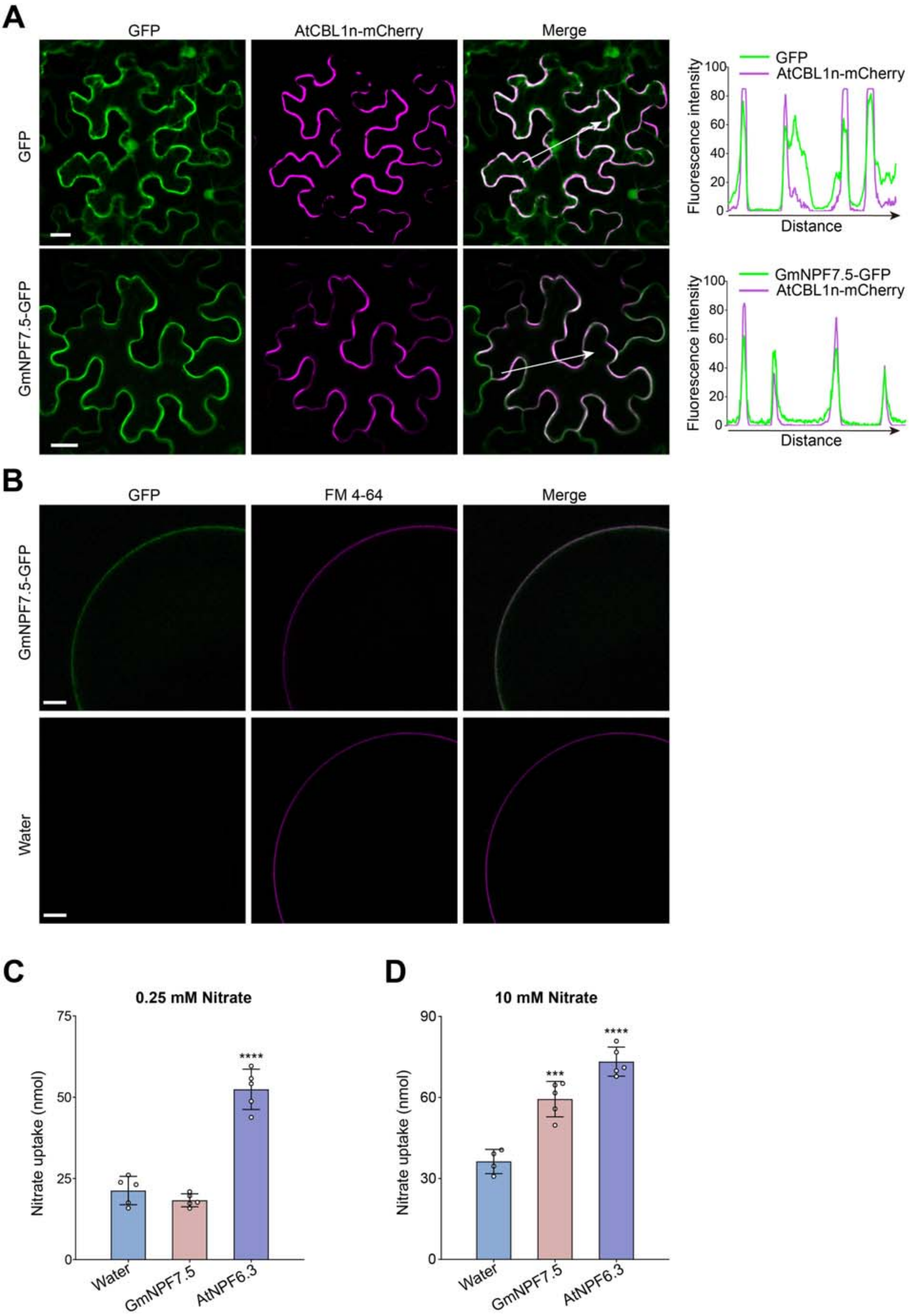
A
GFP
AtCBL1n-mCherry
Merge
GFP
GmNPF7.5-GFP
GFP
AtCBL1n-mCherry
Fluorescence intensity
Distance
GmNPF7.5-GFP
AtCBL1n-mCherry
B
GFP
FM 4-64
Merge
GmNPF7.5-GFP
Water
C
0.25 mM Nitrate
Nitrate uptake (nmol)
Water
GmNPF7.5
AtNPF6.3
D
10 mM Nitrate
Nitrate uptake (nmol)
Water
GmNPF7.5
AtNPF6.3

Figure EV3. Localization of GmNPF7.5 and NO_3^- uptake assay results.

(**A**) Subcellular localization of GmNPF7.5–GFP in *N. benthamiana* leaves, shown by confocal images (left) and fluorescence intensity (right, arrows). Scale bars, 20 μm. (**B**) GmNPF7.5–GFP was localized to the plasma membrane in *Xenopus* oocytes. Scale bars, 100 μm. (**C**) High-affinity (0.25 mM) and (**D**) low-affinity (10 mM) NO_3^- uptake assay using oocytes expressing GmNPF7.5 and AtNPF6.3 (positive control). Data are means ± standard error (SEM). Significance was determined using a two-sided Student's *t* test (****P* < 0.001, *****P* < 0.0001; *n* = 4–5, each replicate contained 2 oocytes). (**C**), *P* values = 0.1993 (GmNPF7.5 vs Water), < 0.0001 (AtNPF6.3 vs Water). (**D**) *P* values = 0.0006 (GmNPF7.5 vs Water), <0.0001 (AtNPF6.3 vs Water). Source data are available online for this figure.

 © The Author(s)

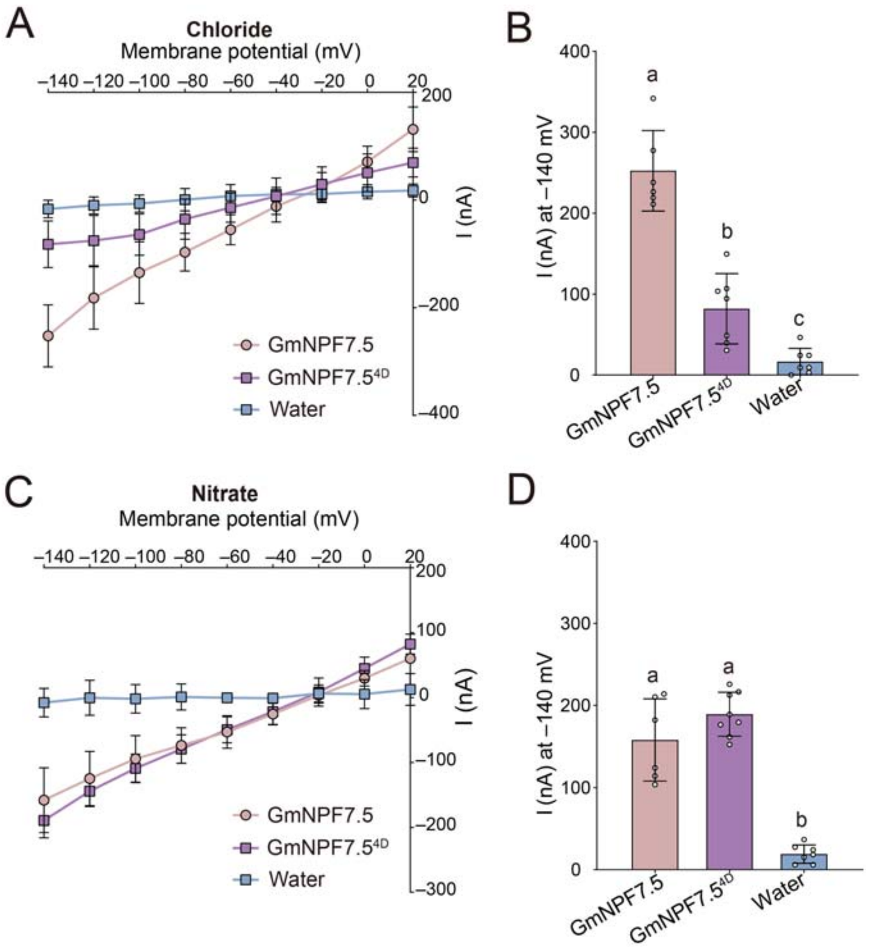

Figure EV4. Effects of phosphomimetic GmNPF7.5 in oocytes.

(**A**) I–V relationship for *Xenopus* oocytes expressing GmNPF7.5, GmNPF7.5^{4D}, and water in basal solution containing 10 mM Cl^- at pH 5.5. (**B**) Cl^--elicited currents at –140 mV recorded from *Xenopus* oocytes in (**A**). (**C**) I–V relationship for *Xenopus* oocytes expressing GmNPF7.5, GmNPF7.5^{4D}, and water in basal solution containing 10 mM NO_3^- at pH 5.5. (**D**) NO_3^--elicited currents at –140 mV recorded from *Xenopus* oocytes in (**C**). Data in (**B**) and (**D**) are means ± SEM ($n = 6–8$ single oocytes). Significance was determined using one-way ANOVA, followed by Tukey's test. Different letters indicate significant differences ($P < 0.05$). (**C**), *P* values < 0.0001 (GmNPF7.5 vs GmNPF7.5^{4D}), <0.0001 (GmNPF7.5 vs Water), 0.0147 (GmNPF7.5^{4D} vs Water). (**D**), *P* values = 0.193 (GmNPF7.5 vs GmNPF7.5^{4D}), <0.0001 (GmNPF7.5 vs Water), <0.0001 (GmNPF7.5^{4D} vs Water). Source data are available online for this figure.

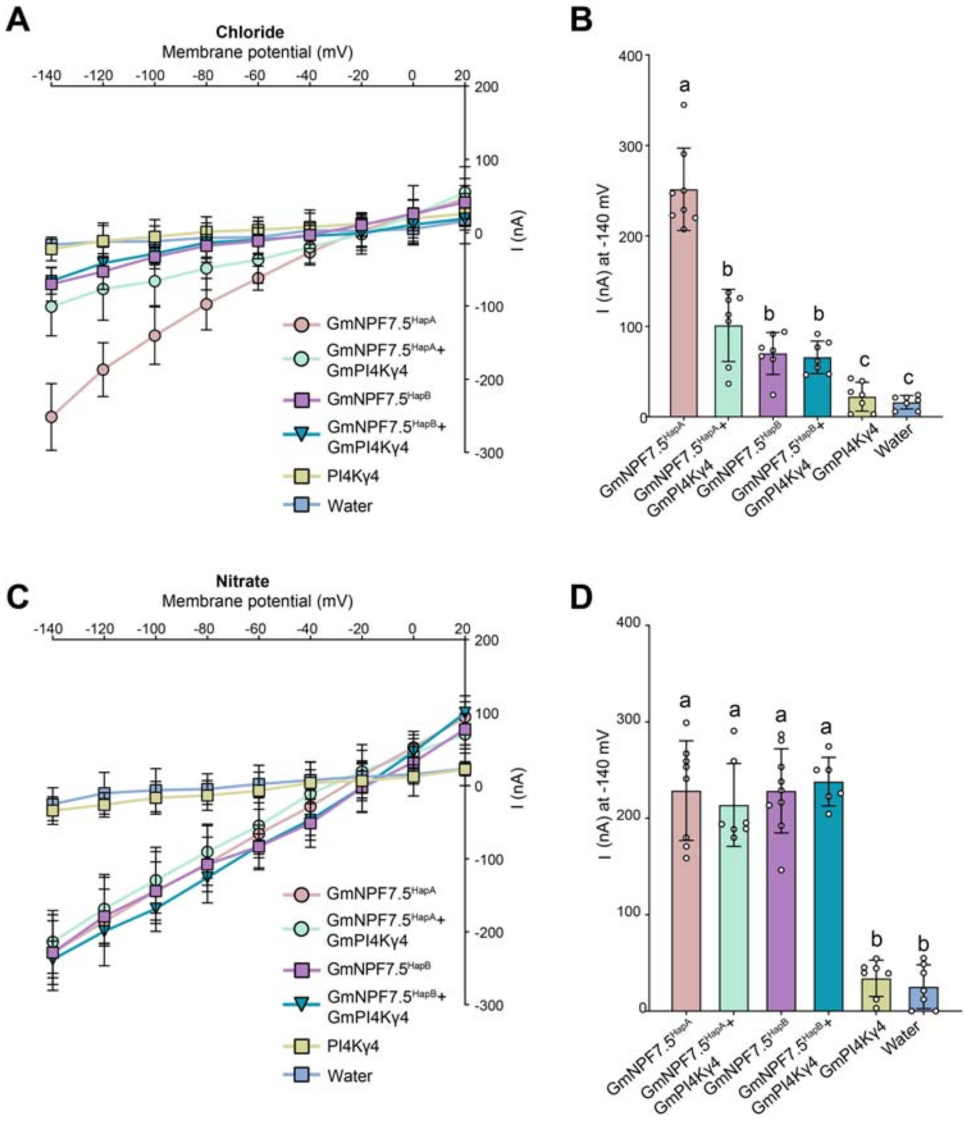

Figure EV5. Effects of GmPI4Kγ4 on the transport activity of two GmNPF7.5 haplotypes in oocytes.

(**A**, **C**) I-V relationship for *Xenopus* oocytes expressing GmNPF7.5^{HapA}, GmNPF7.5^{HapB}, GmPI4Kγ4, GmNPF7.5^{HapA} + GmPI4Kγ4 or GmNPF7.5^{HapB} + GmPI4Kγ4 in basal solution with addition of 10 mM Cl^- (**A**) or 10 mM NO_3^- (**C**) at pH 5.5 ($n = 6$–9). (**B, D**) Cl^--elicited or NO_3^--elicited currents at –140 mV recorded from *Xenopus* oocytes in (**A**) or (**C**). Data in (**B**) and (**D**) are means ± SEM ($n = 6$–9 single oocytes). Significance was determined using one-way ANOVA, followed by Tukey's test. Different letters indicate significant differences ($P < 0.05$). (**B**) P values < 0.0001 (GmNPF7.5^{HapA} vs GmNPF7.5^{HapA} + GmPI4Kγ4), <0.0001 (GmNPF7.5^{HapA} vs GmNPF7.5^{HapB}), <0.0001 (GmNPF7.5^{HapA} vs GmNPF7.5^{HapB} + GmPI4Kγ4), <0.0001 (GmNPF7.5^{HapA} vs GmPI4Kγ4), < 0.0001 (GmNPF7.5^{HapA} vs Water), 0.363 (GmNPF7.5^{HapA} + GmPI4Kγ4 vs GmNPF7.5^{HapB}), 0.2314 (GmNPF7.5^{HapA} + GmPI4Kγ4 vs GmNPF7.5^{HapB} + GmPI4Kγ4), 0.0001 (GmNPF7.5^{HapA} + GmPI4Kγ4 vs GmPI4Kγ4), <0.0001 (GmNPF7.5^{HapA} + GmPI4Kγ4 vs Water), 0.9998 (GmNPF7.5^{HapB} vs GmNPF7.5^{HapB} + GmPI4Kγ4), 0.0405 (GmNPF7.5^{HapB} vs GmPI4Kγ4), 0.015 (GmNPF7.5^{HapB} vs Water), 0.0767 (GmNPF7.5^{HapB} + GmPI4Kγ4 vs GmPI4Kγ4), 0.0302 (GmNPF7.5^{HapB} + GmPI4Kγ4 vs Water), 0.9987 (GmPI4Kγ4 vs Water). (The following is the same order). (**D**), P values = 0.9721, >0.9999, 0.9972, <0.0001, <0.0001, 0.9701, 0.854, <0.0001, <0.0001, 0.9966, <0.0001, <0.0001, <0.0001, <0.0001, 0.9978. Source data are available online for this figure.

 © The Author(s)
